# Network Pharmacology, Molecular Docking, and Molecular Dynamics Simulation to Explore Predicted Molecular Associations Between Fraxini Cortex Constituents and Intestinal Inflammation in Laying Hens

**DOI:** 10.3390/vetsci13090874

**Published:** 2026-08-27

**Authors:** Bochi Zhang, Liying Du, Kai Zhang, Rui Zhao, Chunlei Yang, Xianyi Song

**Affiliations:** College of Animal Science, Shanxi Agricultural University, Taiyuan 030032, China; bochi_zhang@sxau.edu.cn (B.Z.); duliying@sxau.edu.cn (L.D.); zhangkai2255@126.com (K.Z.); zhaorui@sxau.edu.cn (R.Z.); wkyhlycl@126.com (C.Y.)

**Keywords:** Fraxini Cortex, intestinal inflammation, laying hens, network pharmacology, molecular dynamics simulation

## Abstract

Intestinal inflammation can disrupt epithelial integrity, immune homeostasis, and nutrient utilization in laying hens. This entirely in silico study investigated the molecular associations between reported Fraxini Cortex constituents and intestinal inflammation by integrating network pharmacology, chicken-specific bioinformatics, molecular docking, molecular dynamics (MD) simulation, and MM/GBSA analysis. After chemical standardization and SwissADME screening, 38 candidate constituents were retained, yielding 528 nonredundant predicted human targets. Intersection with 1452 intestinal inflammation-related genes identified 170 shared human targets, which were subsequently mapped to 133 nonredundant *Gallus gallus* homologues. A chicken-specific protein–protein interaction network contained 117 non-isolated nodes and 683 interactions, and combined cytoHubba and MCODE analyses identified 13 core candidate targets, including BCL2, SRC, HIF1A, MMP9, ESR1, EGFR, ALB, TLR4, STAT1, PTGS2, SIRT1, JUN, and PPARG. GO and KEGG enrichment associated these targets with receptor and kinase signaling, innate immune recognition, epithelial adhesion and survival, extracellular-matrix remodeling, lipid-mediator metabolism, apoptosis, autophagy, and related processes. Molecular docking of ten candidate compounds against six prioritized targets generated 60 ligand–target combinations, with AutoDock Vina scores ranging from −7.930 to −3.412 kcal/mol. Caffeic acid–MMP9 and scopoletin–ESR1 were further evaluated using three independent 100 ns MD simulations, which showed broadly reproducible conformational behavior across replicates. MM/GBSA calculations yielded mean endpoint binding-energy estimates of −19.92 ± 0.30 kcal/mol for caffeic acid–MMP9 and −21.53 ± 0.32 kcal/mol for scopoletin–ESR1. These findings computationally prioritize caffeic acid, scopoletin, and a group of chicken homologous targets and pathways potentially associated with intestinal inflammation in laying hens and provide testable hypotheses for subsequent chicken-specific experimental validation.

## Simple Summary

Intestinal inflammation can impair gut health, nutrient utilization, and immune balance in laying hens. Fraxini Cortex is a traditional herbal medicine containing multiple reported bioactive compounds, but its possible molecular relationships with intestinal inflammation in poultry remain unclear. In this entirely in silico study, reported Fraxini Cortex constituents were screened and linked with intestinal inflammation-related targets, followed by human-to-*Gallus gallus* orthologue mapping, chicken-specific protein interaction and pathway analyses, molecular docking, molecular dynamics simulations, and MM/GBSA calculations. Thirty-eight candidate constituents and 133 chicken homologous targets were retained, and 13 core candidate targets were further prioritized. Caffeic acid and scopoletin showed prominent positions in the computational analyses, while the predicted targets were mainly associated with immune recognition, receptor and kinase signaling, epithelial integrity, cell survival, tissue remodeling, and lipid-mediator-related processes. Replicated molecular dynamics simulations and binding-energy calculations further characterized the predicted caffeic acid–MMP9 and scopoletin–ESR1 complexes. These findings identify candidate compounds, targets, and pathways that may be relevant to intestinal inflammation in laying hens and provide testable hypotheses for future chicken-specific experimental studies.

## 1. Introduction

The intestine of laying hens is a major site for nutrient digestion and absorption and also functions as an epithelial and immunological barrier. Disturbance of intestinal homeostasis can impair nutrient utilization and compromise productive performance in poultry [1,2,3]. In commercial production, intestinal inflammation may be initiated or aggravated by interacting microbial, nutritional, environmental, and management-related stressors, including enteric pathogens and microbiota disruption, coccidial challenge, poorly digestible dietary components and abrupt feed changes, mycotoxin exposure, heat stress, and other intensive-production pressures [1,4,5]. These challenges can alter microbial ecology, disrupt epithelial tight junctions, and sustain mucosal immune activation. In a chicken model of low-grade chronic intestinal inflammation, a diet rich in non-starch polysaccharides produced segment-specific histological and cytokine/chemokine responses, whereas heat stress aggravated TLR4-NF-κB-associated intestinal inflammation during bacterial challenge [4,5]. Such avian findings provide relevant biological context for laying-hen intestinal health, although responses observed in broilers or other chicken models should not be treated as direct evidence in laying hens. Against the background of reduced routine antibiotic use, these concerns have increased interest in phytogenic strategies for supporting poultry intestinal health [6,7].

Fraxini Cortex (Qinpi), the dried bark of several Fraxinus species, is a traditional botanical drug with a chemically diverse composition [8]. Analytical studies of Cortex Fraxini have directly reported coumarins, secoiridoids, and polyphenolic constituents, including esculin, esculetin, fraxin, fraxetin, scopoletin, and caffeic acid, while also demonstrating variation among botanical species and sample origins [9,10]. These compounds should therefore be regarded as reported constituents rather than as batch-confirmed constituents of the material considered in the present study. Most mechanistic evidence concerning Fraxini Cortex constituents has been generated in mammalian cells or rodent disease models. Esculin/esculetin and scopoletin have been investigated for anti-inflammatory and related pharmacological activities [11,12,13], and caffeic acid has been reported to modulate intestinal inflammatory processes, colitis-associated injury, oxidative stress, and gut microbial composition in mammalian models [14,15,16]. Such findings provide a cross-species mechanistic background but cannot be directly extrapolated to poultry. Poultry-specific evidence remains limited. A recent chicken study evaluated a chemically profiled two-herb formulation containing Fraxini Cortex and Andrographis herba in an Escherichia coli-induced diarrhea model [17]. Because Fraxini Cortex was administered as part of a combination formula rather than as an isolated botanical preparation, those findings cannot establish the efficacy, composition, or active constituents of Fraxini Cortex alone.

The inflammatory biology of the avian intestine also requires species-conscious interpretation. In chickens, TLR4-NF-κB-associated signaling can participate in intestinal inflammatory responses under combined environmental and bacterial stress [5]. Adaptive immune regulation is likewise relevant: jejunal Th17/Treg imbalance has been associated with intestinal immune status and reduced growth performance in chickens, with higher expression of Th17-associated factors such as Stat3, RORγt, IL-6, IL-17A, and IL-21 in lower-body-weight birds [18]. However, IL-17 should not be interpreted as uniformly pathogenic in poultry. In a necrotic-enteritis model, experimental overexpression of avian IL-17 increased intestinal antimicrobial-peptide expression and was associated with lower lesion severity, indicating context-dependent roles in mucosal defense [19]. PI3K-related signaling has also been linked to tight-junction regulation and NF-κB-associated processes in mammalian intestinal inflammation [20], but the conservation, direction, and functional importance of such relationships in the chicken intestine cannot be assumed from mammalian data alone. These differences support the use of chicken-specific interaction and annotation resources when translating molecular hypotheses to poultry.

Network pharmacology provides a framework for organizing multi-component and multi-target hypotheses for complex botanical medicines [21]. However, many commonly used compound-target prediction and disease-gene resources are predominantly human-oriented; direct interpretation of these associations as chicken biology would therefore introduce a substantial cross-species assumption. Accordingly, human-derived target associations should be mapped to *Gallus gallus* orthologues before chicken-specific protein–protein interaction and functional enrichment analyses are performed. In the present entirely in silico study, literature- and database-derived Fraxini Cortex constituents were integrated with target prediction, human-to-*Gallus gallus* orthologue mapping, chicken-specific STRING network analysis and functional enrichment, molecular docking, and molecular dynamics simulation [22,23,24]. No physical Fraxini Cortex batches, live birds, avian cells, intestinal organoids, experimentally challenged animals, or newly collected biological samples were used. The objective was therefore to computationally prioritize reported Fraxini Cortex constituents, candidate chicken homologous targets, and molecular associations related to intestinal inflammation and to generate testable hypotheses for subsequent chemical and chicken-specific experimental validation, rather than to demonstrate therapeutic efficacy or an established mechanism in laying hens.

## 2. Materials and Methods

### 2.1. Research Design and Reporting Scope

This study was designed as an entirely in silico investigation integrating database mining, network pharmacology, bioinformatics enrichment, AlphaFold-based protein structures where required, molecular docking, and molecular dynamics simulation. No live laying hens or other animals were enrolled, treated, experimentally challenged, sampled, or euthanized, and no newly collected animal tissues or biological specimens were used. Accordingly, all compound–target associations, core targets, enriched pathways, docking poses, binding energies, and dynamic stability parameters reported in this study are computational predictions or simulation outputs. They are intended to generate mechanistic hypotheses and prioritize candidate compounds and targets for subsequent validation. These results must not be interpreted as direct evidence that Fraxini Cortex produces a therapeutic effect in laying hens, regulates the expression or activity of the predicted targets in vivo, or exhibits direct anti-inflammatory or antimicrobial activity. Because no live animals or newly collected animal samples were used, institutional animal ethics approval was not applicable. Experimental confirmation in chicken cells, intestinal organoids, ex vivo tissues, laying-hen models, or biological samples is required before biological or therapeutic conclusions can be established.

### 2.2. Collection and Screening of Fraxini Cortex Candidate Constituents

Reported constituents of Fraxini Cortex were collected from TCMSP, BATMAN-TCM, and HERB and cross-checked using PubMed, ITCMDB, and PubChem. Database and literature retrieval was completed on 9 August 2026 using “Fraxini Cortex”, “Cortex Fraxini”, “Qinpi”, and “Fraxinus bark” as principal search terms. Chemical identities were standardized using PubChem CIDs and canonical SMILES, and duplicate or synonymous records were merged. Records lacking a valid PubChem CID or unambiguous chemical structure were excluded. The remaining compounds were evaluated using SwissADME (access date 9 August 2026). Candidates were retained when gastrointestinal absorption was predicted as High and at least two of the five drug-likeness filters—Lipinski, Ghose, Veber, Egan, and Muegge—showed zero violations. Prior anti-inflammatory or intestinal-protective activity was not used as an inclusion criterion. The complete screening process and compound-level information are provided in Supplementary S1.

### 2.3. Target Prediction and Collection of Intestinal Inflammation-Related Targets

Potential targets of the screened Fraxini Cortex constituents were predicted using SwissTargetPrediction. Only predicted targets with a probability score > 0.1 were retained, after which target names were standardized according to UniProt gene nomenclature and duplicate records were removed. Intestinal inflammation-related genes were retrieved from GeneCards and OMIM using the predefined phenotype-level search term “Intestinal Inflammation”. For GeneCards, records with a relevance score > 65 were retained, whereas eligible OMIM-associated genes were collected and merged with the GeneCards dataset followed by deduplication. Because these target-prediction and disease-gene resources are predominantly human-oriented, the resulting compound-related and disease-related target sets were treated as human gene sets at this stage. Their intersection was subsequently identified to obtain shared human targets, which were then subjected to human-to-*Gallus gallus* ortholog mapping before chicken-specific network and enrichment analyses.

### 2.4. Human-to-Gallus gallus Ortholog Mapping

To improve species relevance, the shared human targets were converted to chicken homologous genes before subsequent network analyses. Human gene symbols were mapped to *Gallus gallus* using the biomaRt package (version 2.62.1) in R. Human and chicken Ensembl gene annotations were queried, and the corresponding *Gallus gallus* gene symbols were extracted. Mapping was rerun against Ensembl release 116; 138/170 targets had at least one chicken orthology record, 32 were unmapped, and 133 representative high-confidence orthologues were retained (120 one-to-one, 6 one-to-many, and 7 many-to-many). When multiple high-confidence candidates were available, one representative was selected deterministically by exact human/chicken gene-symbol match, then preference for an assigned Gallus symbol, and finally ascending Gallus Ensembl gene ID. Gene nomenclature was standardized before downstream analysis. Only the successfully mapped *Gallus gallus* targets were used for STRING-based PPI construction, GO/KEGG enrichment, network-topology analysis, and structural prioritization. The retained chicken target set and available mapping information are provided in Supplementary Table S2 to document the cross-species conversion procedure.

### 2.5. PPI Network Construction and Core Target Screening

The successfully mapped *Gallus gallus* targets were submitted to STRING for protein–protein interaction (PPI) analysis, with the organism set to *Gallus gallus* and the minimum interaction score set to 0.400. The resulting interaction network was imported into Cytoscape (version 3.10.3), and isolated nodes and duplicate edges were removed. Network topology was evaluated using four cytoHubba algorithms: Degree, maximal clique centrality (MCC), Betweenness, and Closeness. The top 20 nodes ranked by each algorithm were retained, and their intersection was defined as the multi-algorithm candidate set. MCODE was subsequently applied to identify highly connected network modules using Degree Cutoff = 2, Node Score Cutoff = 0.2, K-Core = 2, and Max Depth = 100. The final core-target set was defined as the intersection between the multi-algorithm cytoHubba candidate set and the targets identified within the MCODE core modules. Complete PPI topology, the four Top20 rankings, MCODE module information, and final core-target screening results are provided in Supplementary Table S2. Degree was used only as a descriptive connectivity metric within this prespecified multistep prioritization scheme and was not interpreted as evidence of biological importance, therapeutic relevance, or target modulation.

### 2.6. GO Functional and KEGG Pathway Enrichment Analyses

The 133 mapped *Gallus gallus* targets were subjected to Gene Ontology (GO) and Kyoto Encyclopedia of Genes and Genomes (KEGG) enrichment analyses using R version 4.2.1 and the clusterProfiler package version 4.6.2. Chicken gene symbols were converted to the corresponding Entrez Gene identifiers before enrichment analysis. GO analysis included biological process (BP), cellular component (CC), and molecular function (MF), whereas KEGG enrichment was performed using the *Gallus gallus* organism code “gga”. The effective annotated backgrounds comprised 11,696 genes for BP, 11,619 for CC, 12,431 for MF, and 6489 for KEGG analysis. Enrichment significance was evaluated using multiple-testing-adjusted *p* values, with adjusted *p* < 0.05 considered statistically significant. For visualization, significantly enriched terms were ranked objectively according to adjusted *p* value, GeneRatio, and enriched gene count rather than being manually selected according to presumed biological relevance. GO and KEGG enrichment results, including term identifiers, GeneRatio, background ratio, enriched genes, *p* values, adjusted *p* values, and gene counts, were retained for Supplementary Reporting. The Benjamini–Hochberg (BH) method was used for multiple-testing correction. The minimum gene-set size was 5 and no maximum was specified. The exact historical annotation/KEGG release was not preserved and therefore was not retrospectively assigned. Complete archived GO/KEGG outputs are provided in Supplementary Table S2 and Supplementary Table S2_GO_KEGG_complete_archived.xlsx.

### 2.7. Construction of the Compound–Target–Pathway–Disease Network

The relationships among the 38 retained Fraxini Cortex candidate constituents, 133 mapped *Gallus gallus* targets, significantly enriched KEGG pathways, and intestinal inflammation were integrated using Cytoscape. To avoid subjective pathway selection, KEGG pathways were ranked according to adjusted *p* value, GeneRatio, and enriched gene count, and the top 20 statistically significant pathways were used for network construction. Nodes represented Fraxini Cortex, candidate constituents, chicken targets, KEGG pathways, and intestinal inflammation, whereas edges represented predicted compound–target or target–pathway associations. In addition, the overlap between the 13 final PPI core targets and genes enriched in each KEGG pathway was calculated to evaluate the representation of core network targets across enriched pathways. Pathways were therefore interpreted according to both enrichment statistics and core-target involvement rather than pathway name alone. Network topology was calculated in Cytoscape to characterize the connectivity among compounds, targets, and pathways.

### 2.8. Molecular Docking

Within the 13 final chicken-specific core targets, the upper quartile of the Degree distribution was Q3 = 33; targets with Degree ≥ Q3 were therefore defined a priori as the high-connectivity core tier for structural prioritization. This deterministic within-core threshold yielded six receptors—BCL2 (Degree = 41), SRC (40), HIF1A (39), MMP9 (33), ESR1 (33), and EGFR (33). The ligand panel comprised the ten candidate constituents present in the archived docking workflow. During the final audit, this archived ten-compound panel was found not to coincide exactly with the Degree > Q3 high-connectivity tier of the revised 38-compound network; therefore, no retrospective claim is made that the ten archived ligands were selected by a Degree cutoff or represented the ten biologically most important compounds. Complete topology for all 38 retained compounds and the distinction between descriptive connectivity tiers and the archived docking subset are provided in Supplementary Table S1B. Chicken-specific AlphaFold models were used for BCL2 (AF-Q00709-F1), EGFR (AF-P13387-F1), ESR1 (AF-P06212-F1), HIF1A (AF-Q9YIB9-F1), and MMP9 (AF-A0A8V0YXL9-F1), whereas SRC was represented by the experimentally determined *Gallus gallus* structure PDB 2PTK. Receptors and ligands were converted to PDBQT format, with hydrogen atoms and partial atomic charges retained in receptor files and rotatable bonds defined for ligands. Receptor-specific global docking boxes encompassing the protein structures were applied. Docking was performed using AutoDock Vina v1.2.7 with exhaustiveness = 50, random seed = 1000, and four CPU threads. The lowest Vina score for each ligand–target pair was retained for comparison. Target-specific receptor information, grid centers, box dimensions, and docking parameters are provided in Supplementary Table S2. To assess computational reproducibility without retrospectively assigning undocumented settings to the archived run, all 60 ligand–receptor combinations were additionally rerun as a separate sensitivity/reproducibility analysis while holding the exact archived *Gallus gallus* receptor PDBQT inputs and receptor-specific global search boxes fixed. Ligand microspecies were standardized at pH 7.4 and assigned RDKit Gasteiger partial charges; caffeic acid was represented as monoanionic caffeate and the remaining nine ligands as neutral parent species. No additional ligand energy minimization was introduced. The rerun used AutoDock Vina v1.2.7 with scoring = vina, exhaustiveness = 50, random seed = 1000, CPU = 4, num_modes = 9, energy_range = 3 kcal/mol, and grid spacing = 0.375 Å. Because the upstream receptor-preparation pH was not preserved historically, no retrospective receptor protonation state was invented; reproducibility is instead anchored to the exact receptor PDBQT input files. Complete receptor/ligand preparation audits, grid parameters, rerun scores, and reproducibility records are provided in Supplementary Table S2.

Because five docking receptors were AlphaFold-predicted models, model confidence was assessed directly from the per-residue pLDDT values stored in the AlphaFold PDB B-factor field. For each best-ranked Vina pose involving an AlphaFold receptor, predicted interaction-site residues were operationally defined as receptor heavy-atom residues lying within 4.0 Å of any ligand heavy atom. Local confidence was summarized as the mean and minimum pLDDT of these contact residues using the standard qualitative bands of very high confidence (pLDDT ≥ 90), confident (70 to <90), low (50 to <70), and very low (<50). This local pLDDT analysis evaluates structural confidence of the sampled receptor region but does not establish that a global-box docking pose represents a biologically authentic binding pocket. Predicted aligned error (PAE) files were not available for the supplied models, and no PAE-based claim was made. As an independent same-species experimental-site benchmark for SRC, the annotated imatinib-binding site of the wild-type *Gallus gallus* SRC kinase-domain structure PDB 2OIQ (X-ray diffraction, 2.07 Å) was compared with the residue contacts of all nine SRC poses generated in the standardized global-box rerun. This comparison was treated as a binding-site correspondence check rather than same-structure co-crystallized-ligand RMSD redocking. Complete whole-model and pose-local confidence data are provided in Supplementary Table S2 and the accompanying structural-audit dataset.

### 2.9. Molecular Dynamics Simulation

Two nonredundant ligand–target complexes were selected for molecular dynamics (MD) simulations by jointly considering docking performance, core-target status, and representation of distinct candidate constituents and protein targets. The caffeic acid–MMP9 complex, which showed one of the lowest Vina scores in the complete docking matrix (−7.689 kcal/mol), and the scopoletin–ESR1 complex (−6.890 kcal/mol) were selected for subsequent simulations. Three independent 100 ns MD simulations were performed for each complex using the WebMD workflow with GROMACS under identical simulation settings. Proteins were parameterized using the Amber ff14SB force field and protonated using PDB2PQR/PROPKA at pH 7.0, whereas ligands were parameterized using GAFF2 with AM1-BCC partial charges. Each system was solvated with TIP3P water using an approximately 10 Å solvent padding, neutralized, and supplemented with NaCl to an ionic strength of approximately 0.15 M. Following energy minimization, each system underwent approximately 500 ps of NVT equilibration and 1 ns of NPT equilibration with positional restraints on solute heavy atoms. Berendsen pressure coupling was used during NPT equilibration, whereas production simulations employed the V-rescale thermostat and Parrinello–Rahman barostat at 310 K and approximately 1 bar. A 2 fs integration timestep was used, bonds involving hydrogen atoms were constrained, long-range electrostatic interactions were treated using the particle mesh Ewald method, and the nonbonded interaction cutoff was approximately 1.0 nm. Trajectories were aligned using the protein backbone and analyzed for protein, complex, and ligand RMSD, Cα RMSF, radius of gyration (Rg), solvent-accessible surface area (SASA), protein–ligand hydrogen bonds, and Gibbs free-energy landscapes. Each of the three independent trajectories was analyzed separately, and the resulting dynamic profiles were compared across replicates.

### 2.10. MM/GBSA Binding Free-Energy Calculation

Binding free energies of the caffeic acid–MMP9 and scopoletin–ESR1 complexes were calculated using gmx_MMPBSA v1.6.5 for each of three independent 100 ns MD trajectories. ΔG_bind was calculated as G_complex − G_receptor − G_ligand using Amber ff14SB and GAFF2 parameters. The Generalized Born model was applied with igb = 8, mbondi3 radii (PBRadii = 4), internal/external dielectric constants of 5/78.5, 0.15 M salt, and 310 K. Frames were sampled throughout each production trajectory every 10 stored frames (approximately 1 ns intervals). Per-residue decomposition was performed using idecomp = 2 for residues within 6 Å of the ligand. Entropic contributions were not included. Each trajectory was analyzed independently, and replicate-level results were summarized across the three simulations.

### 2.11. Data Processing and Statistical Analysis

Network-topology data, enrichment outputs, molecular docking scores, and molecular dynamics (MD) results were organized and processed using Microsoft Excel and R version 4.2.1. Network parameters were treated as descriptive topological measures, whereas molecular docking scores were used for within-protocol ranking and were not subjected to inferential statistical testing. For MD analysis, three independently initialized 100 ns simulations were conducted for each selected ligand–target complex. Each trajectory was analyzed separately, and replicate-level summary values were subsequently calculated across the three independent simulations. Where appropriate, results are expressed as the mean ± standard deviation (SD) across independent MD replicates. Temporal variation among trajectory frames within a single simulation was used only to characterize within-trajectory fluctuations and was not treated as independent replication. The same replicate-based approach was applied to RMSD, RMSF, radius of gyration, SASA, hydrogen-bond analyses, and subsequent binding-energy calculations. Unless otherwise specified, all computational analyses were descriptive and no biological inferential statistics were applied because no experimental animals, biological samples, or independently sampled biological observations were included in this study.

## 3. Results

### 3.1. Screening of Fraxini Cortex Candidate Constituents and Identification of Shared Human Targets and Their Gallus gallus Homologues

After database integration and chemical-identity standardization, 91 candidate constituent records of Fraxini Cortex were obtained. Six records lacking a valid PubChem CID or an unambiguous chemical structure were excluded, leaving 85 compounds for SwissADME evaluation. Among these, 35 compounds were excluded because of predicted low gastrointestinal absorption. Of the remaining 50 compounds with predicted high gastrointestinal absorption, 12 failed to meet the prespecified drug-likeness criterion. Consequently, 38 candidate constituents were retained for subsequent analyses. SwissTargetPrediction analysis with a probability threshold > 0.1 yielded 528 nonredundant predicted human targets after gene-name standardization and deduplication.

**Figure 1:**
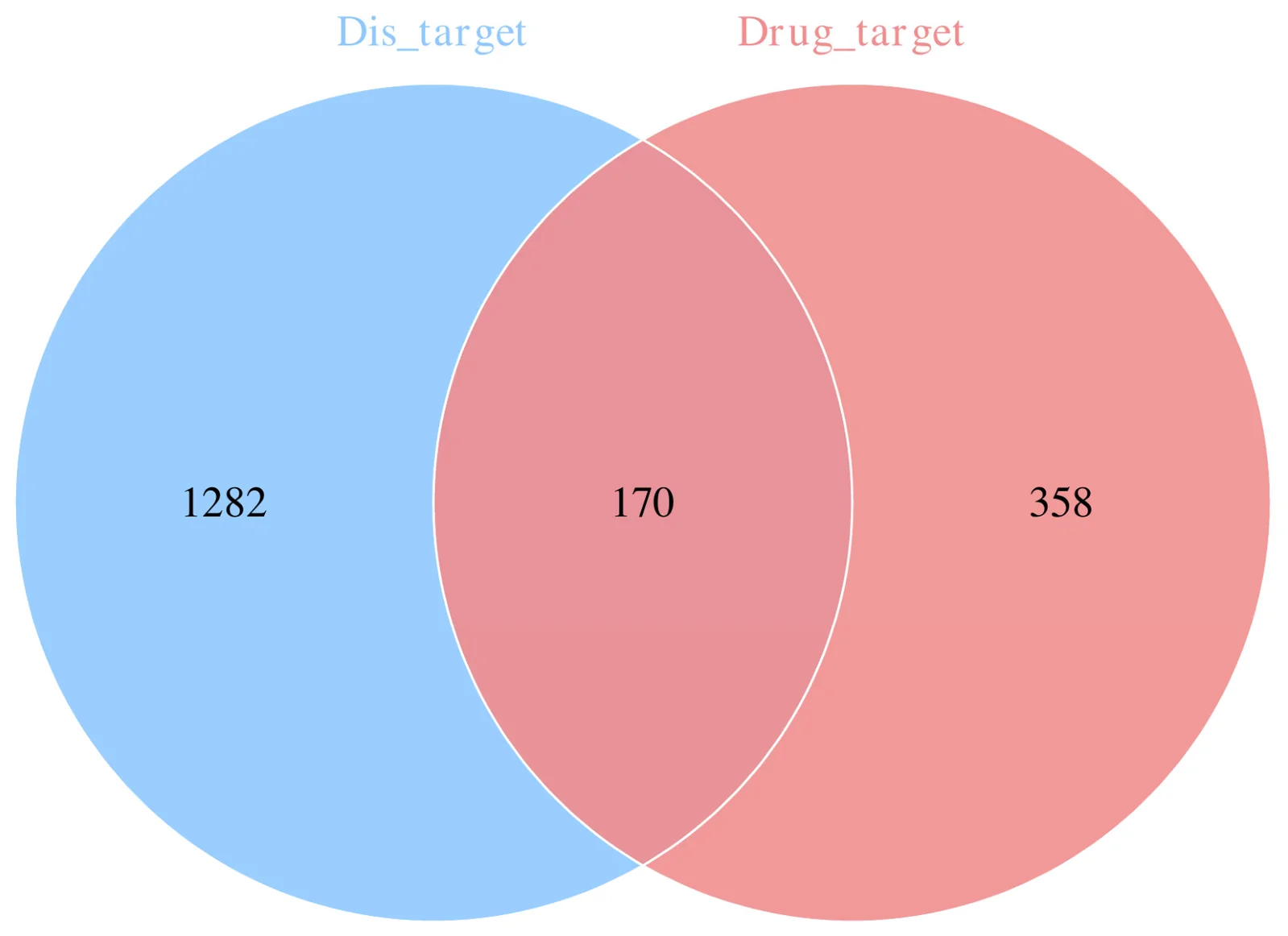
Venn diagram of predicted Fraxini Cortex-associated targets and intestinal inflammation-related targets.

GeneCards and OMIM yielded 1452 nonredundant human genes associated with intestinal inflammation. Intersection of the 528 compound-related targets with the 1452 disease-related targets identified 170 shared human targets, with 358 compound-specific targets and 1282 disease-specific targets (Figure 1). The 170 shared human targets were subsequently subjected to human-to-*Gallus gallus* orthologue mapping using biomaRt. After removal of records without a retained chicken assignment and deduplication of chicken gene symbols, 133 nonredundant *Gallus gallus* homologous targets were retained for subsequent chicken-specific network and enrichment analyses. The complete constituent-screening information and human-to-chicken mapping records are provided in Supplementary Tables S1 and S2, respectively.

### 3.2. Fraxini Cortex–Compound–Chicken Homologous Target Network Analysis

**Figure 2:**
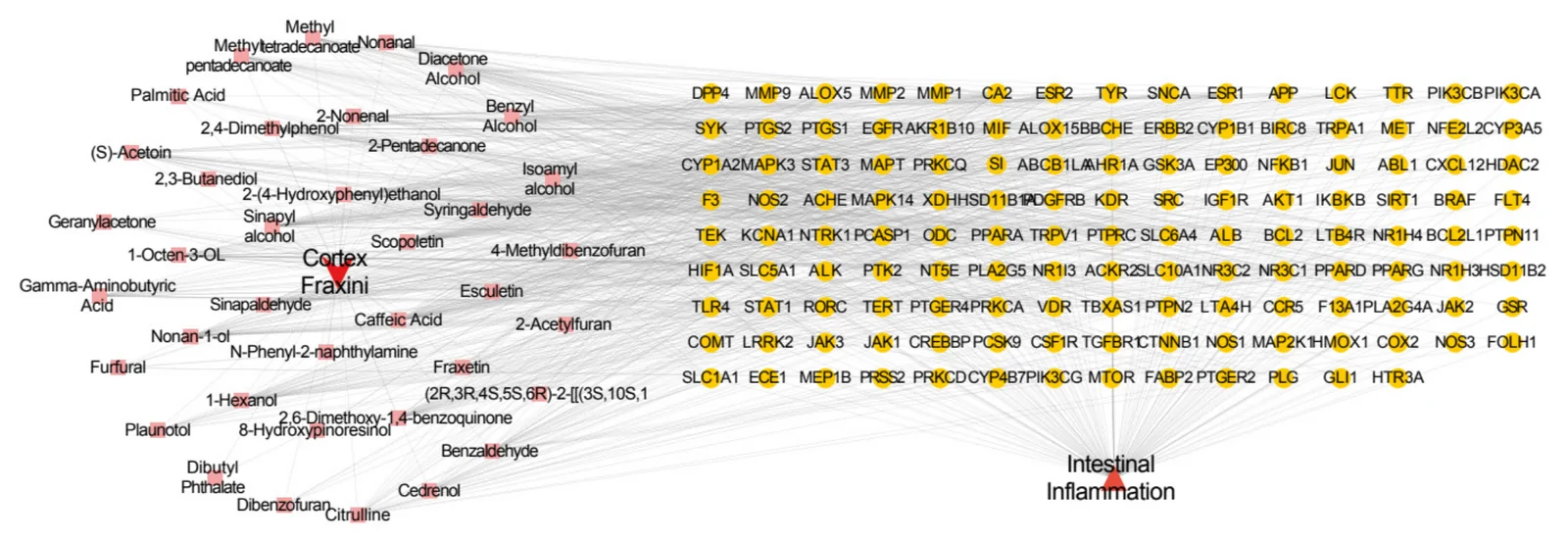
Fraxini Cortex-compound-shared target network. Nodes represent Fraxini Cortex, candidate compounds, intestinal inflammation, and mapped *Gallus gallus* shared targets; edges represent predicted compound-target associations.

**Figure 3:**
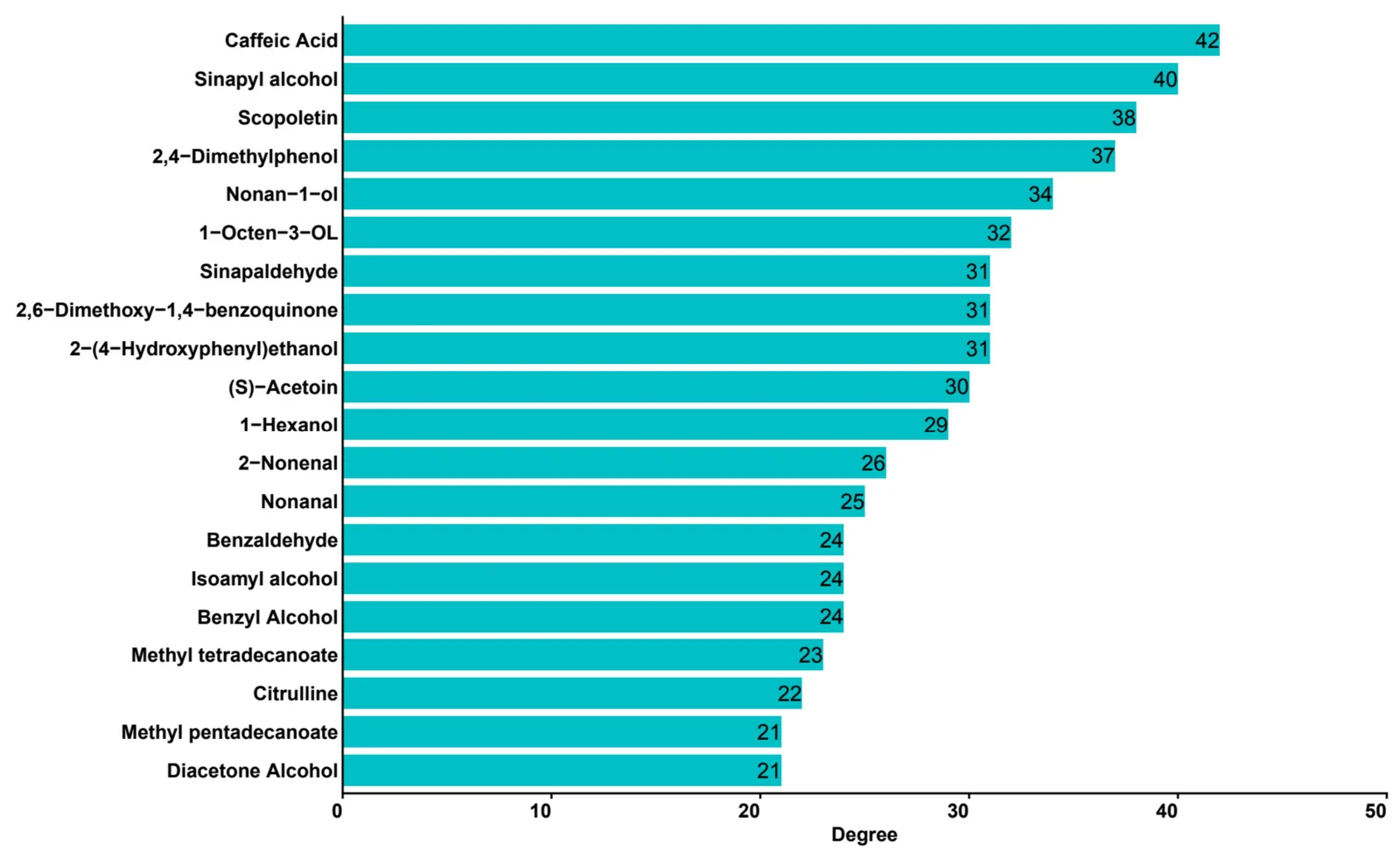
Degree ranking of candidate compounds in the Fraxini Cortex-compound-shared target network.

A Fraxini Cortex–compound–target network was constructed using the 38 retained candidate constituents and 133 mapped *Gallus gallus* homologous shared targets. The network contained 173 nodes and 871 edges, comprising one Fraxini Cortex node, one intestinal inflammation node, 38 compound nodes, and 133 chicken homologous target nodes (Figure 2). Among the compound nodes, the 12 highest Degree values were observed for caffeic acid (Degree = 42), sinapyl alcohol (40), scopoletin (38), 2,4-dimethylphenol (37), nonan-1-ol (34), 1-octen-3-ol (32), sinapaldehyde (31), 2-(4-hydroxyphenyl)ethanol (31), 2,6-dimethoxy-1,4-benzoquinone (31), (S)-acetoin (30), 1-hexanol (29), and 2-nonenal (26) (Figure 3). Among the target nodes, CA2 (Degree = 31), ESR2 (24), ACHE (24), ESR1 (22), PTGS2 (22), CYP1B1 (19), HSD11B1A (19), and ALOX5 (18) showed relatively high connectivity. Degree was interpreted as a descriptive measure of network connectivity rather than evidence of pharmacological activity or biological efficacy. Complete compound-level topological parameters are provided in Supplementary Table S1B. For transparency, the compound Degree distribution had a median of 21, Q1 = 7.75, and Q3 = 29.75; Degree > Q3 (integer Degree ≥ 30) defined a descriptive high-connectivity tier of 10 compounds. This topology tier was not retrospectively used to redefine the archived ten-compound docking panel. Specifically, 1-hexanol (Degree = 29) and 2-nonenal (26) were present in the archived docking panel, whereas 2,4-dimethylphenol (37) and 2,6-dimethoxy-1,4-benzoquinone (31) belonged to the descriptive high-connectivity tier but were not in the archived panel. Accordingly, connectivity categories are reported as network descriptors rather than as evidence of compound abundance, activity, or an original docking-selection rule.

### 3.3. Chicken-Specific PPI Network and Core-Target Screening by cytoHubba and MCODE

**Figure 4:**
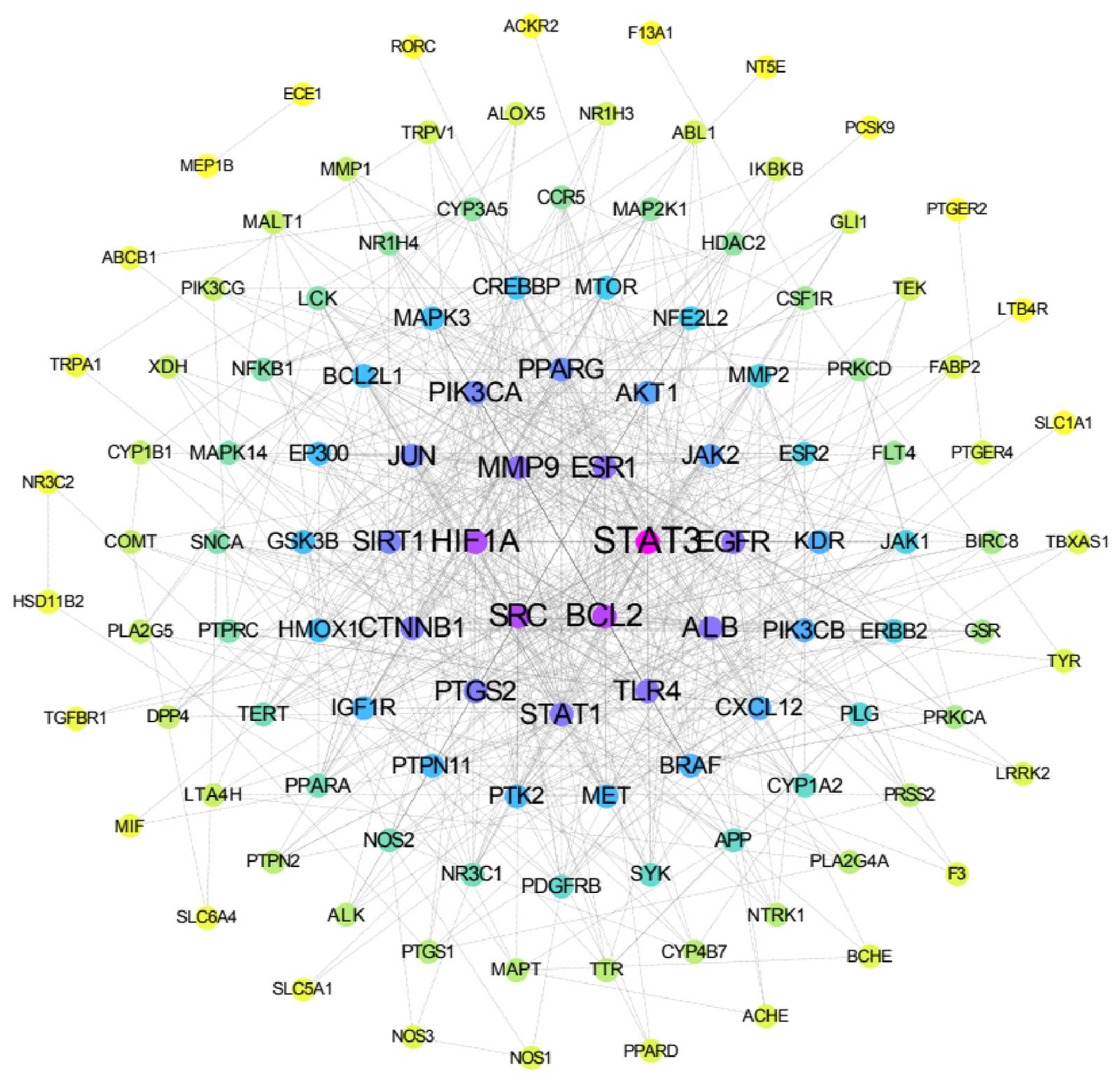
*Gallus gallus* STRING PPI network of mapped shared targets. The network contained 117 nodes and 683 interactions after removal of isolated nodes.

The 133 mapped *Gallus gallus* homologous shared targets were submitted to STRING to construct a chicken-specific protein–protein interaction (PPI) network. After removal of isolated nodes, the network contained 117 nodes and 683 interaction edges (Figure 4). The nodes with the highest Degree values included STAT3 (Degree = 53), BCL2 (41), SRC (40), HIF1A (39), MMP9 (33), ESR1 (33), EGFR (33), ALB (32), TLR4 (32), STAT1 (31), PTGS2 (30), and CTNNB1 (30).

**Figure 5:**
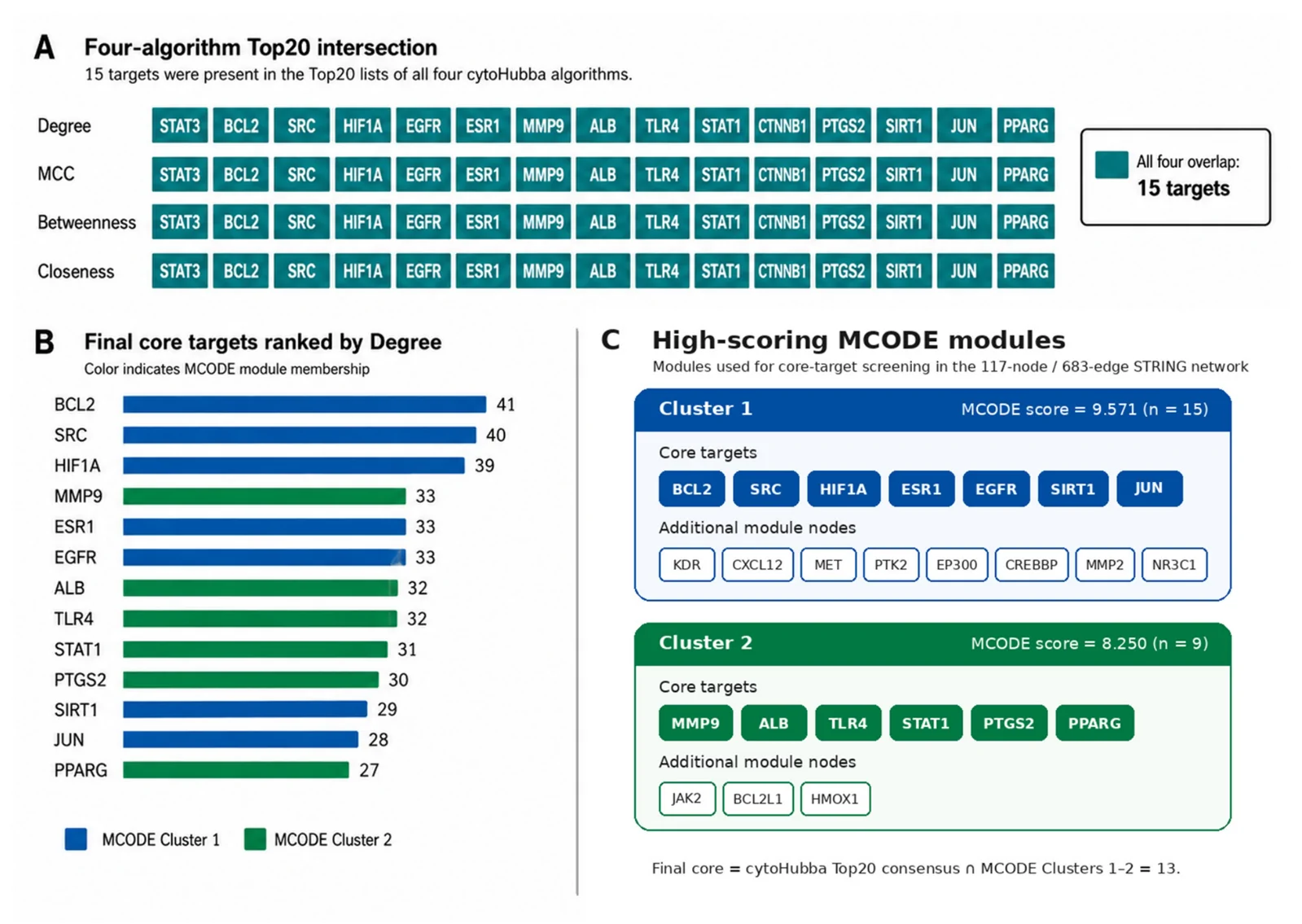
cytoHubba screening and MCODE module analysis of the chicken-specific PPI network. (**A**) Four-algorithm Top20 intersection containing 15 consensus targets. (**B**) Degree ranking of the final 13 core candidate targets, colored by MCODE module membership. (**C**) Two high-scoring MCODE modules used for core-target screening.

To further prioritize core targets, the top 20 nodes ranked by Degree, maximal clique centrality (MCC), Betweenness, and Closeness were compared. The four-algorithm intersection contained 15 consensus targets: STAT3, BCL2, SRC, HIF1A, EGFR, ESR1, MMP9, ALB, TLR4, STAT1, CTNNB1, PTGS2, SIRT1, JUN, and PPARG (Figure 5A). MCODE analysis identified two high-scoring modules comprising 24 targets in total. Cluster 1 had an MCODE score of 9.571 and contained 15 nodes, whereas Cluster 2 had an MCODE score of 8.250 and contained 9 nodes (Figure 5C).

Intersecting the 15-target cytoHubba consensus set with the two MCODE modules yielded 13 final core candidate targets: BCL2, SRC, HIF1A, MMP9, ESR1, EGFR, ALB, TLR4, STAT1, PTGS2, SIRT1, JUN, and PPARG (Figure 5B). STAT3 and CTNNB1 were included in the four-algorithm consensus set but were not retained in the two high-scoring MCODE modules. The Degree values of the final 13 targets were 41, 40, 39, 33, 33, 33, 32, 32, 31, 30, 29, 28, and 27, respectively. The upper quartile of this 13-target Degree distribution was Q3 = 33; applying the prespecified within-core criterion Degree ≥ Q3 yielded BCL2, SRC, HIF1A, MMP9, ESR1, and EGFR (*n* = 6), which were advanced to molecular docking. This threshold was used only for structural prioritization after the cytoHubba–MCODE consensus step and does not imply greater biological importance or therapeutic relevance. Complete PPI topology, cytoHubba rankings, MCODE membership, and core-target screening results are provided in Supplementary Table S2. A robustness reanalysis of the fully re-audited 117-node/685-edge STRING network recovered the same 13 core targets and six structurally prioritized receptors as the archived 683-edge network.

### 3.4. Chicken-Specific GO Functional and KEGG Pathway Enrichment Analyses

**Figure 6:**
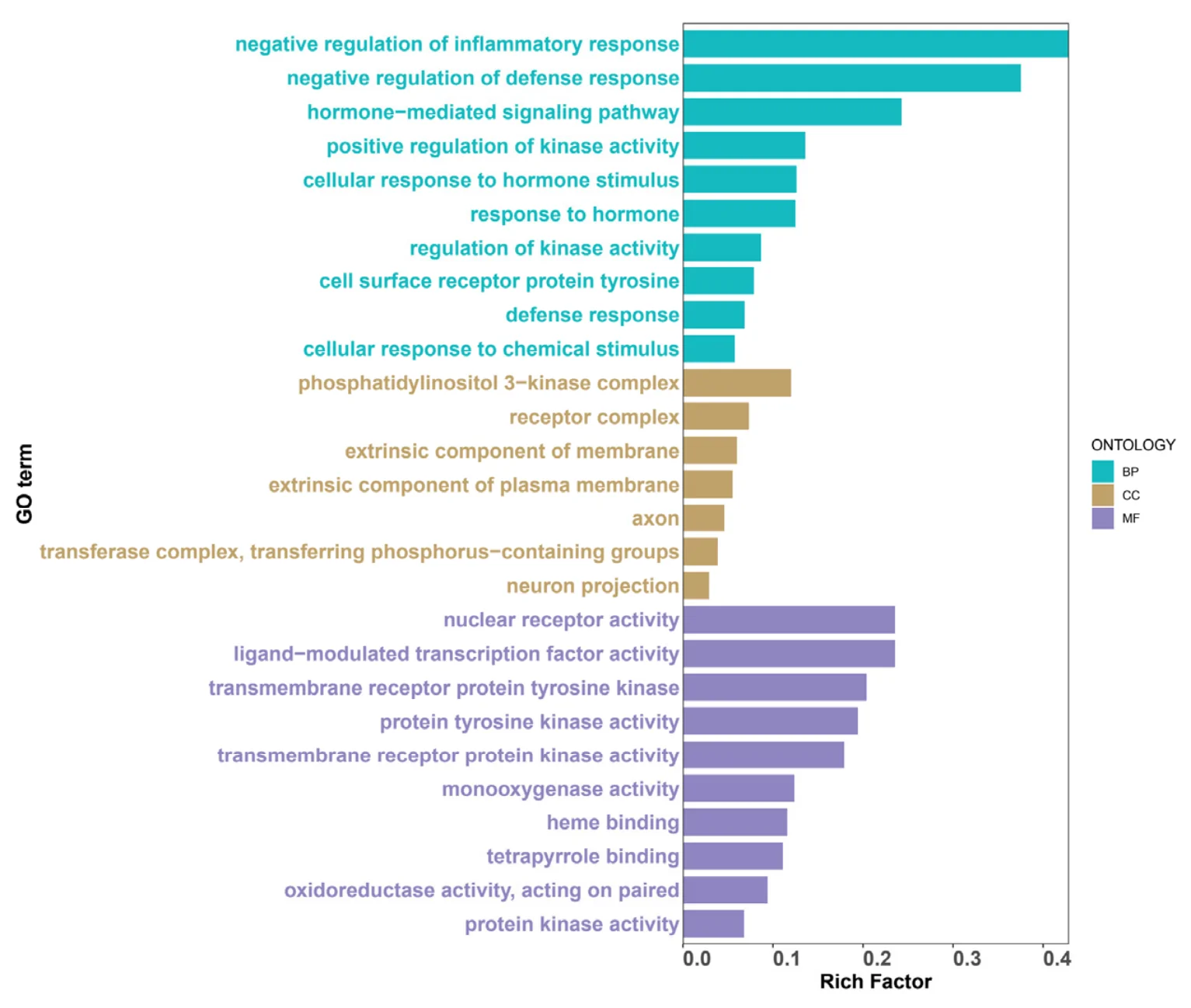
GO enrichment analysis of mapped *Gallus gallus* homologous targets.

GO enrichment analysis of the 133 mapped *Gallus gallus* homologous targets identified 228 terms with nominal *p* < 0.05. After multiple-testing adjustment, 109 terms remained significant, including 78 biological process (BP), 2 cellular component (CC), and 29 molecular function (MF) terms. Highly ranked GO terms were mainly associated with hormone responses, receptor tyrosine kinase signaling, regulation of kinase activity, defense responses, receptor complexes, and protein kinase-related molecular functions (Figure 6).

**Figure 7:**
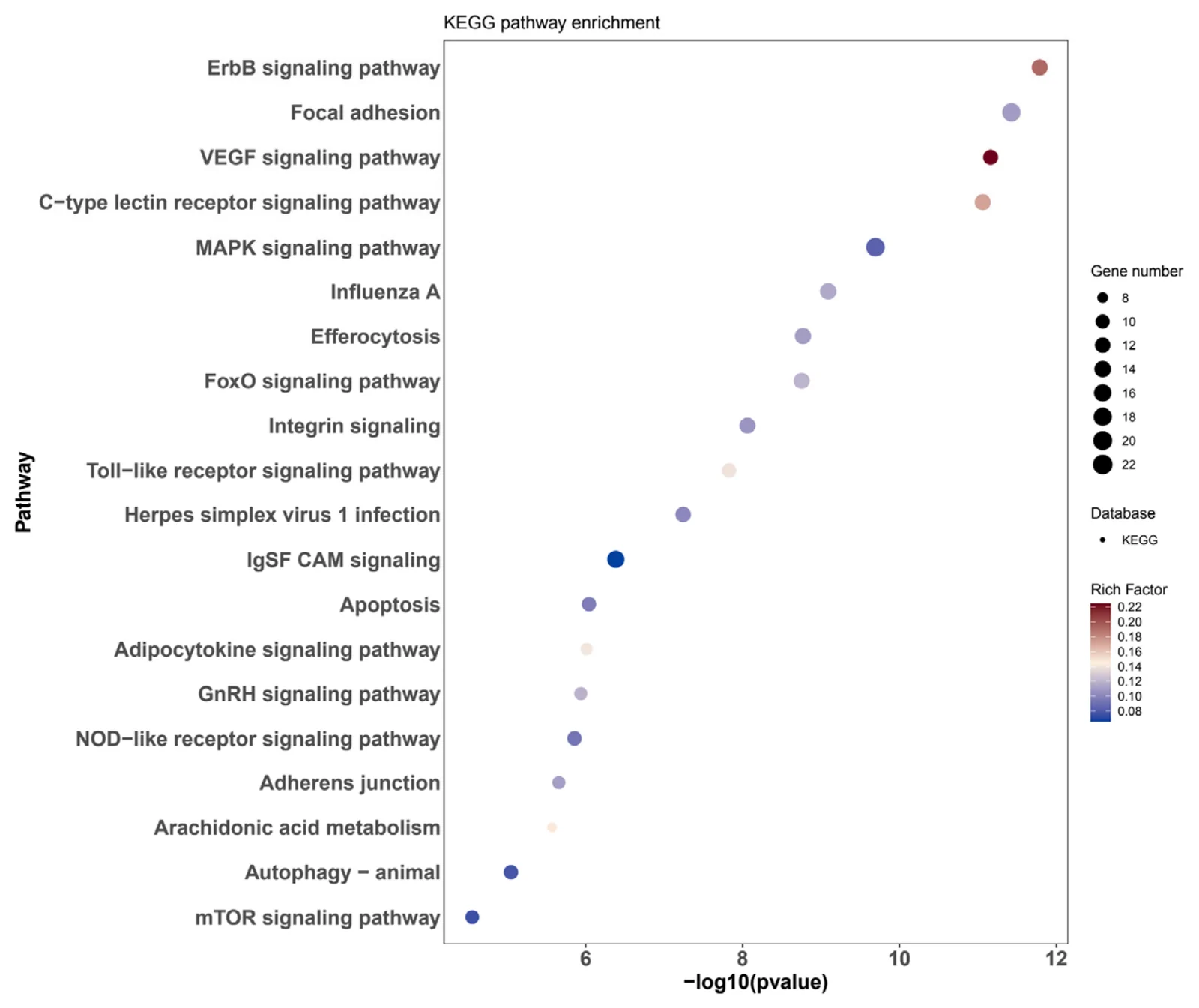
KEGG pathway enrichment analysis of mapped *Gallus gallus* homologous targets.

KEGG enrichment analysis identified 44 pathways with nominal *p* < 0.05, of which 39 remained significant after multiple-testing adjustment. Highly ranked pathways included ErbB signaling, focal adhesion, C-type lectin receptor signaling, VEGF signaling, MAPK signaling, efferocytosis, FoxO signaling, Toll-like receptor signaling, NOD-like receptor signaling, apoptosis, adherens junction, arachidonic acid metabolism, autophagy, and mTOR signaling (Figure 7). Integration with the 13 final PPI core targets showed relatively high core-target representation in the NOD-like receptor, C-type lectin receptor, efferocytosis, Toll-like receptor, ErbB, and focal adhesion pathways. Complete archived GO/KEGG outputs, including BH-nonsignificant rows, are provided in Supplementary Table S2 and the accompanying enrichment workbook.

### 3.5. Compound–Target–Pathway–Disease Network Analysis

**Figure 8:**
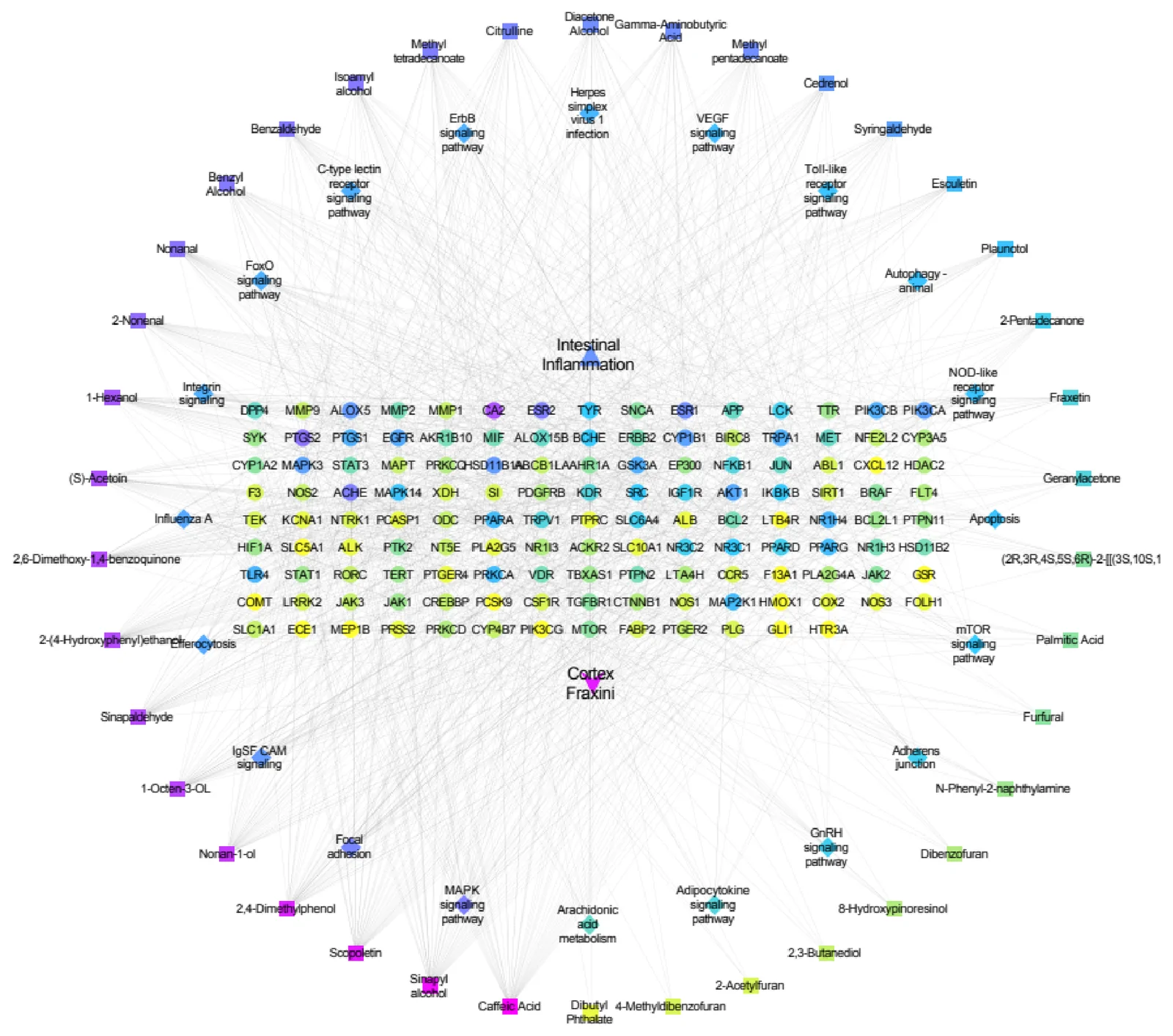
Integrated Fraxini Cortex–compound–target–pathway–disease network.

An integrated Fraxini Cortex–compound–target–pathway–disease network was constructed using the 38 retained candidate constituents, 133 mapped *Gallus gallus* homologous targets, and the top 20 adjusted-significant KEGG pathways. After removal of database-source nodes, the network contained 193 nodes and 1046 edges, including one Fraxini Cortex node, one intestinal inflammation node, 38 compound nodes, 133 target nodes, and 20 pathway nodes (Figure 8). Candidate constituents including caffeic acid, scopoletin, sinapyl alcohol, and sinapaldehyde were connected with targets such as PTGS2, TLR4, STAT1, PPARG, ESR1, SRC, EGFR, and MMP9, which were further linked to pathways including MAPK, Toll-like receptor, C-type lectin receptor, FoxO, focal adhesion, VEGF, and ErbB signaling.

### 3.6. Molecular Docking Analysis

**Figure 9:**
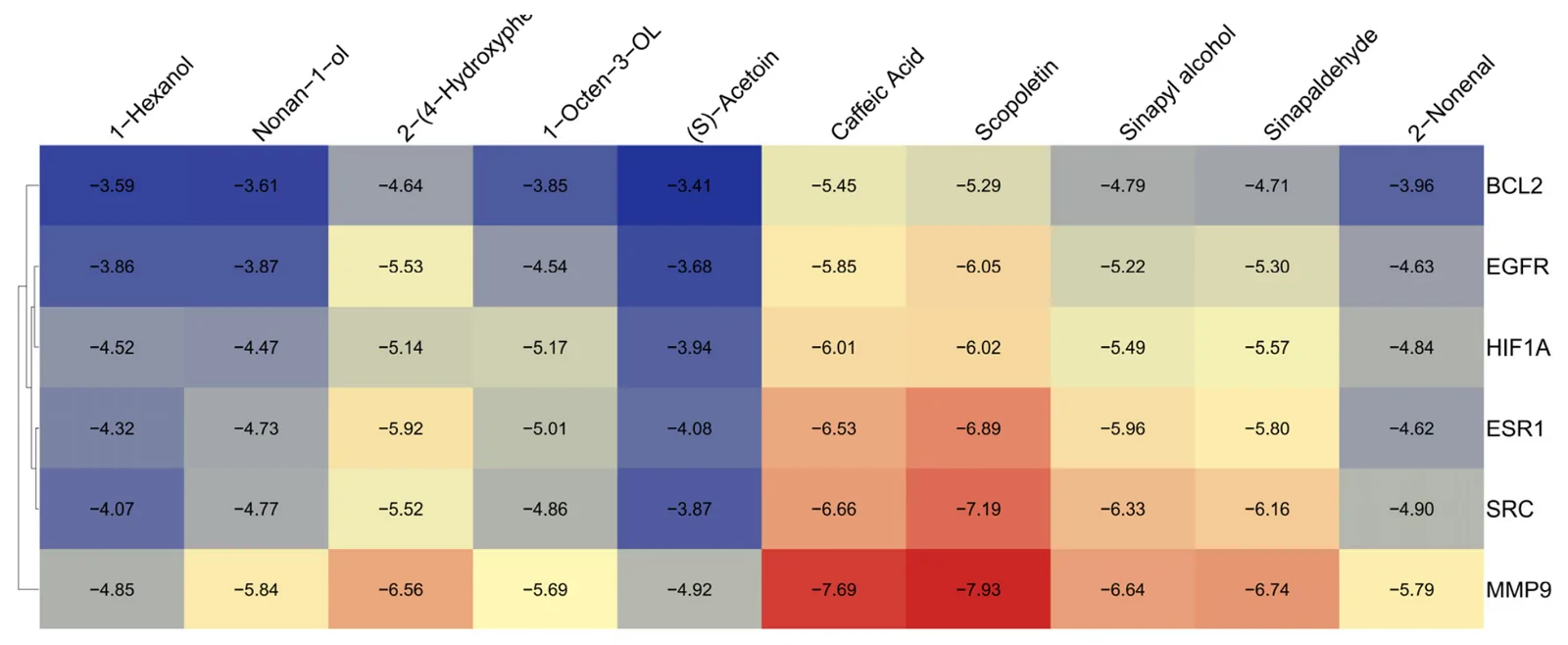
Heatmap of AutoDock Vina scores for 60 ligand–target combinations involving ten candidate constituents and six prioritized chicken-specific core targets.

The six Degree ≥ Q3 high-connectivity targets within the final 13-target core set—BCL2, SRC, HIF1A, MMP9, ESR1, and EGFR—were subjected to molecular docking with the ten candidate constituents contained in the archived docking workflow, yielding 60 ligand–target combinations. AutoDock Vina scores ranged from −7.930 to −3.412 kcal/mol (Figure 9). The lowest scores were observed for MMP9–scopoletin (−7.930 kcal/mol), MMP9–caffeic acid (−7.689 kcal/mol), SRC–scopoletin (−7.192 kcal/mol), ESR1–scopoletin (−6.890 kcal/mol), and MMP9–sinapaldehyde (−6.736 kcal/mol). The complete score distribution across all 60 combinations is presented in Figure 9.

As a separate reproducibility/sensitivity assessment, all 60 combinations were rerun under the fully specified protocol described above. The rerun scores ranged from −7.399 to −3.548 kcal/mol and showed strong concordance with the archived docking matrix (Pearson r = 0.981; Spearman rho = 0.985; mean absolute difference = 0.119 kcal/mol). Caffeic acid-MMP9 and scopoletin-ESR1 remained highly ranked (1/60 and 5/60, respectively). Because this rerun was designed to test reproducibility rather than replace the archived analysis, the original docking figures and original score presentation were retained unchanged; the complete standardized rerun matrix is reported in Supplementary Table S2. AlphaFold confidence was then evaluated at the actual receptor regions sampled by the best-ranked poses. Across the 50 AlphaFold receptor–ligand combinations, 32/50 had a local mean pLDDT ≥ 90 and 49/50 had a local mean pLDDT ≥ 70. Only three poses contained any contact residue with pLDDT < 50, and all three involved BCL2. The two complexes taken forward to MD were located in locally high-confidence regions: caffeic acid/caffeate-MMP9 contacted 13 residues with a mean pLDDT of 94.67 and a minimum of 88.19, whereas scopoletin-ESR1 contacted 11 residues with a mean pLDDT of 97.42 and a minimum of 95.31. These values support the local structural order of the sampled regions but do not validate pocket identity. For SRC, the annotated imatinib site in the independent wild-type chicken SRC structure 2OIQ comprised 15 protein residues. The lowest-scoring scopoletin-SRC mode (−7.227 kcal/mol) did not overlap these annotated site residues, whereas modes 2, 4, and 7 recovered 6, 7, and 6 site residues, respectively. Thus, the receptor-encompassing search sampled both experimentally defined kinase-pocket-compatible and noncanonical regions, and the lowest Vina score alone did not establish active-site identity.

**Figure 10:**
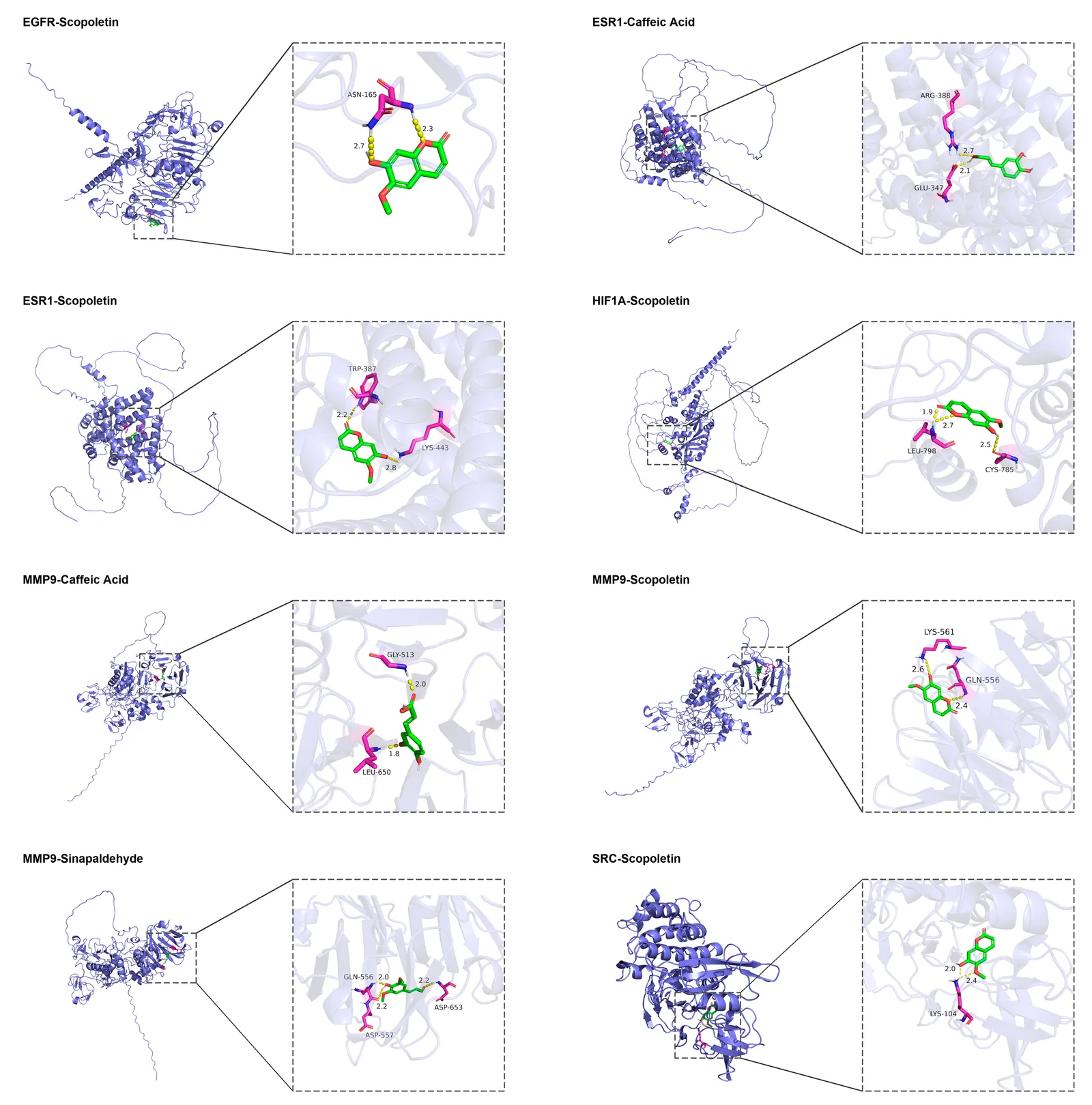
Representative predicted docking poses of prioritized compound–target combinations.

Representative predicted docking poses were visualized for EGFR–scopoletin, ESR1–caffeic acid, ESR1–scopoletin, HIF1A–scopoletin, MMP9–caffeic acid, MMP9–scopoletin, MMP9–sinapaldehyde, and SRC–scopoletin (Figure 10). These docking results were used for comparative computational prioritization and were not interpreted as experimentally measured binding affinities or direct evidence of target modulation.

### 3.7. Molecular Dynamics Simulation Analysis

**Figure 11:**
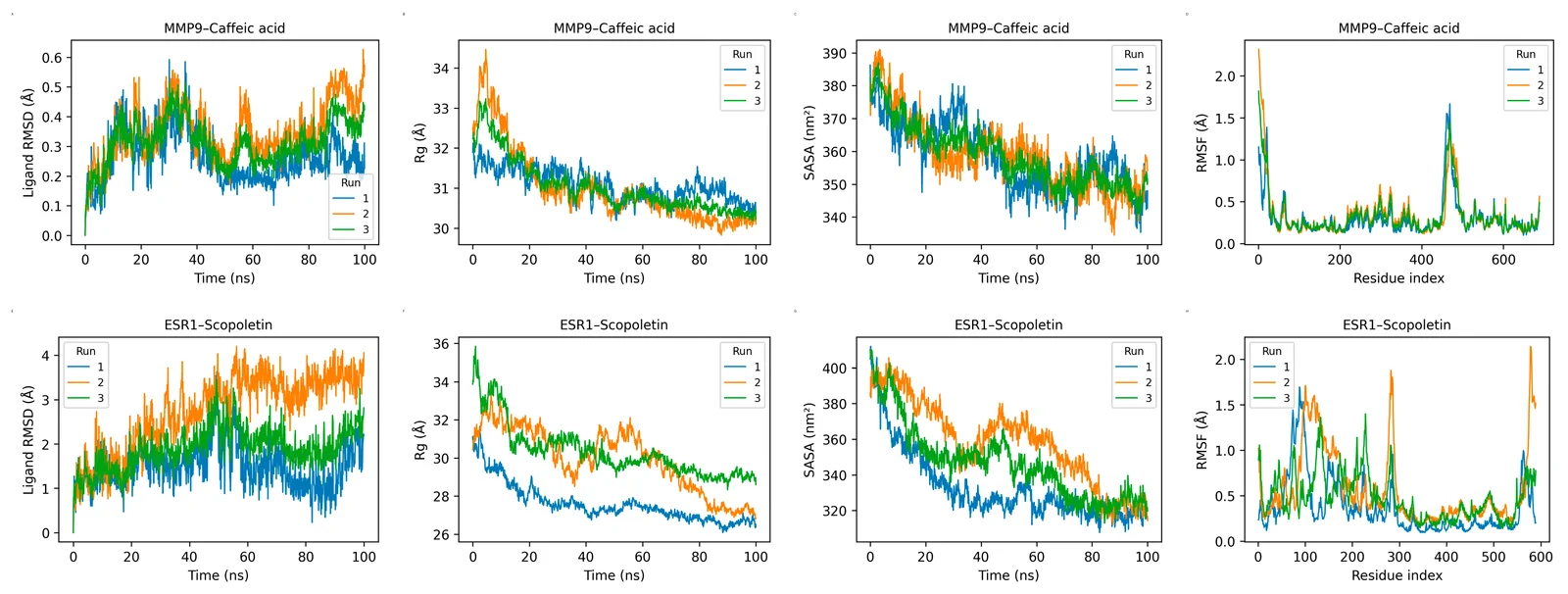
Overlay plots of the three independent 100 ns trajectories for the caffeic acid–MMP9 and scopoletin–ESR1 complexes, showing Rg, SASA, ligand RMSD, and RMSF.

**Figure 12:**
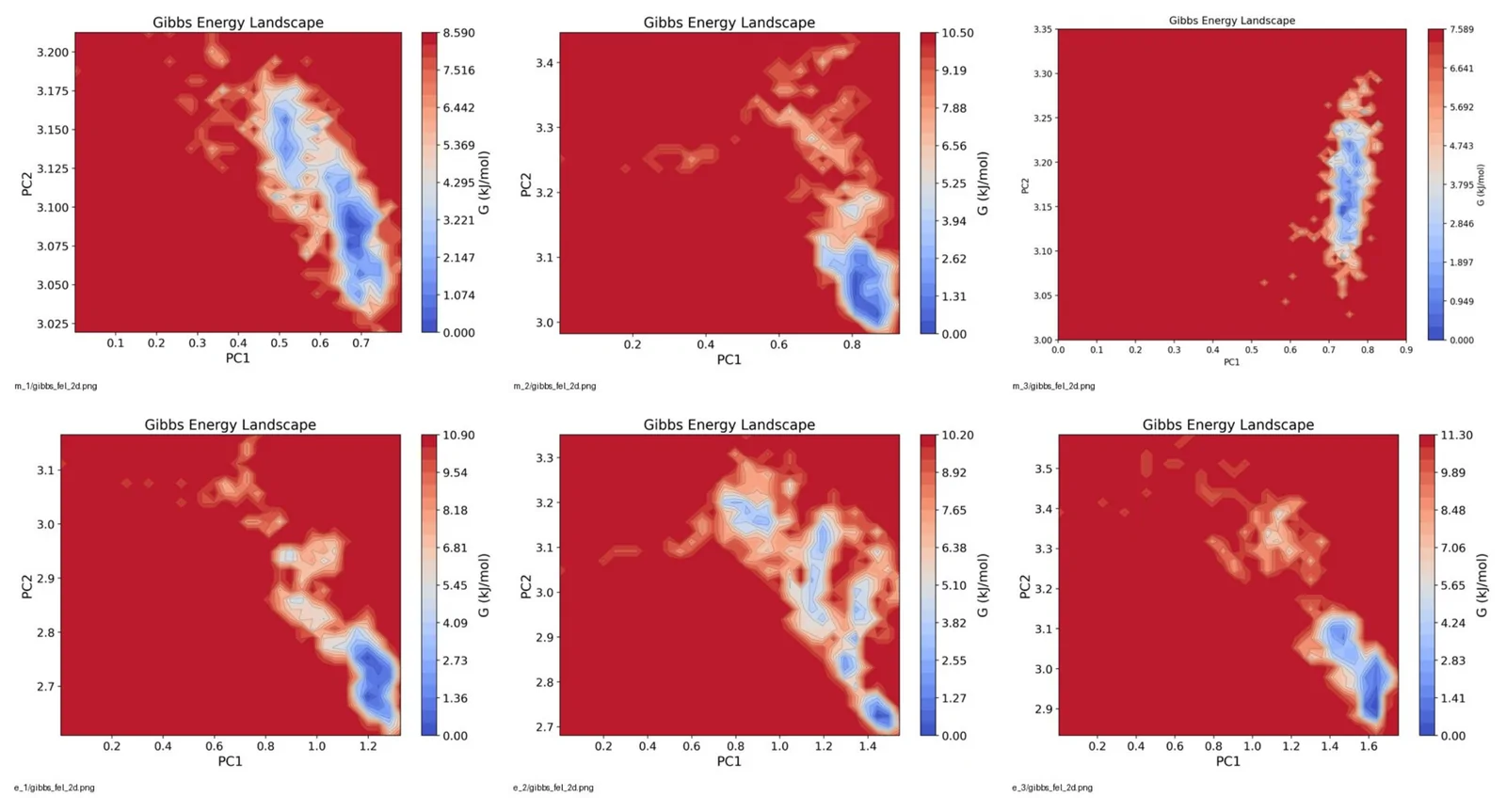
Two-dimensional Gibbs free-energy landscapes of the three independent trajectories for the caffeic acid–MMP9 complex (m_1–m_3) and the scopoletin–ESR1 complex (e_1–e_3).

**Figure 13:**
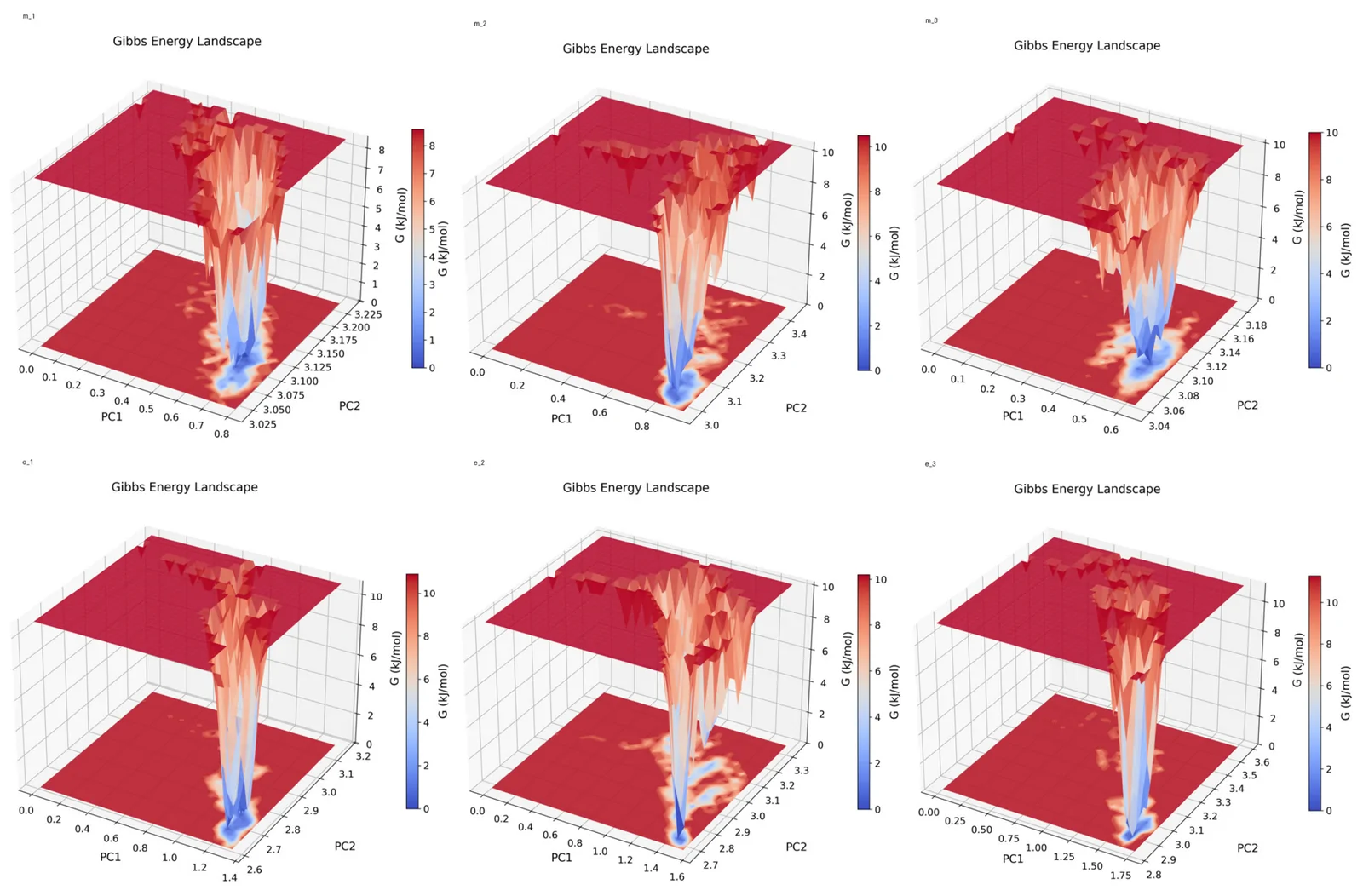
Three-dimensional Gibbs free-energy surfaces of the six independent trajectories, including three simulations for the caffeic acid–MMP9 complex (m_1–m_3) and three simulations for the scopoletin–ESR1 complex (e_1–e_3).

To further evaluate the post-docking conformational behavior of the prioritized ligand–target systems, three independent 100 ns molecular dynamics simulations were performed for the caffeic acid–MMP9 and scopoletin–ESR1 complexes under the same simulation protocol. The resulting trajectories were analyzed using the radius of gyration (Rg), solvent-accessible surface area (SASA), ligand RMSD, residue-level RMSF, Gibbs free-energy landscapes, and representative three-dimensional conformational snapshots. The corresponding results are shown in Figure 11, Figure 12 and Figure 13.

For the caffeic acid–MMP9 complex, the three independent trajectories showed broadly comparable dynamic behavior (Figure 11). The Rg and SASA profiles displayed similar time-dependent trends and converged to comparable late-stage levels. Ligand RMSD remained comparatively low throughout the simulations, and the RMSF curves exhibited similar fluctuation patterns across the three runs, with the major peaks mainly located in terminal and loop-associated regions.

For the scopoletin–ESR1 complex, the three independent trajectories also showed comparable conformational evolution over 100 ns, although moderate replicate-dependent variation was observed (Figure 11). The Rg and SASA curves displayed consistent overall trends among the three runs, whereas ligand RMSD and RMSF indicated reproducible but relatively broader conformational sampling than that observed for the caffeic acid–MMP9 system.

The two-dimensional Gibbs free-energy landscapes showed that both complexes sampled localized low-energy conformational basins rather than exhibiting unrestricted structural drift (Figure 12). The corresponding three-dimensional Gibbs free-energy surfaces of the six independent trajectories are presented together in Figure 13. Taken together, these repeated simulations supported reproducible dynamic accommodation of both prioritized ligand–target systems under the present computational protocol.

### 3.8. MM/GBSA Binding Free-Energy Analysis

**Figure 14:**
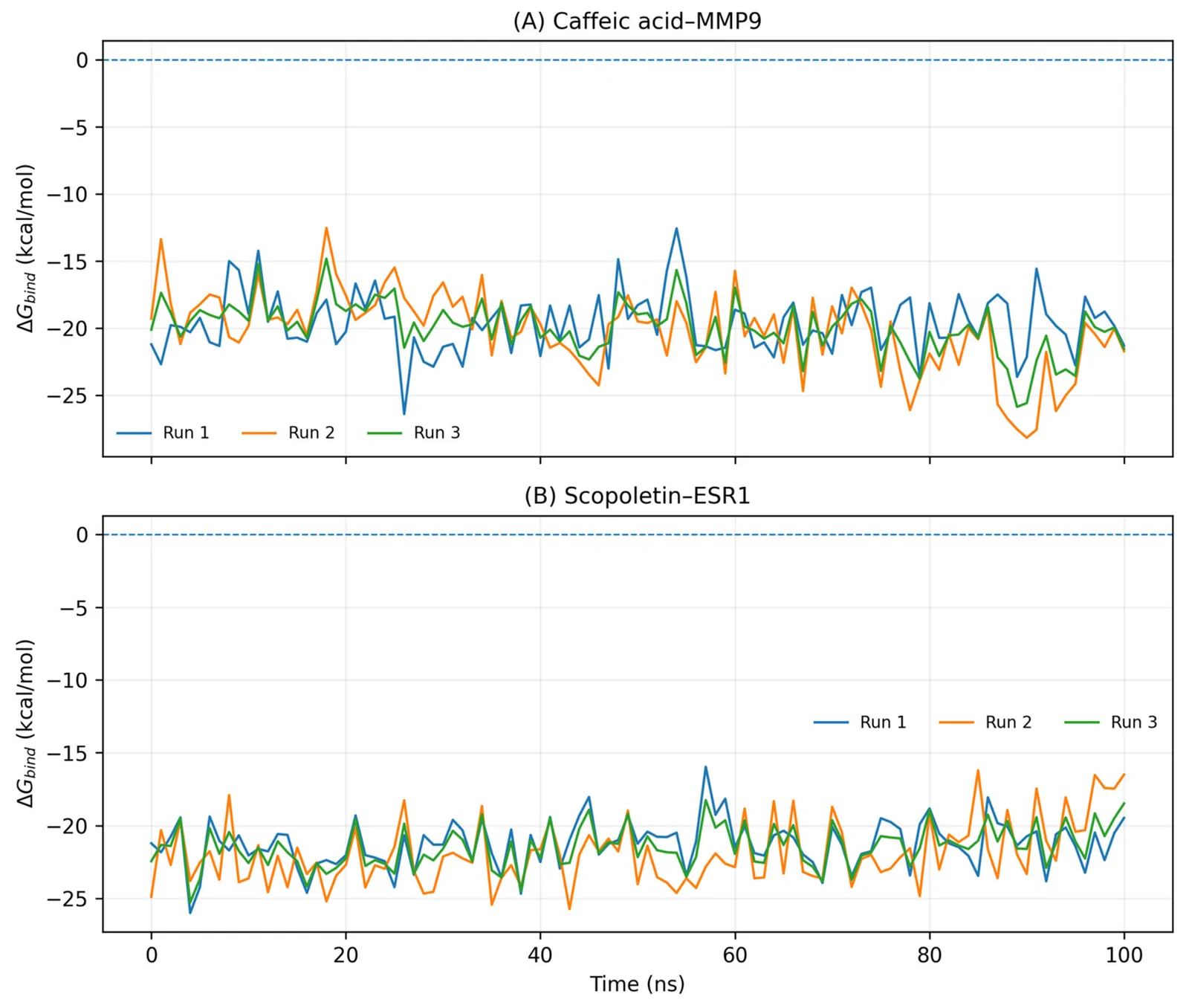
Frame-resolved MM/GBSA ΔG_bind values from the three independent 100 ns trajectories of the caffeic acid–MMP9 (**A**) and scopoletin–ESR1 (**B**) complexes.

MM/GBSA calculations were performed separately for the three independent 100 ns trajectories of each prioritized complex. For caffeic acid–MMP9, the mean ΔG_bind values of Runs 1–3 were −19.61, −20.21, and −19.95 kcal/mol, respectively, corresponding to a replicate-level mean of −19.92 ± 0.30 kcal/mol. For scopoletin–ESR1, the corresponding values were −21.25, −21.88, and −21.46 kcal/mol, with a replicate-level mean of −21.53 ± 0.32 kcal/mol. The frame-resolved ΔG_bind values remained negative throughout the analyzed trajectories for both systems (Figure 14).

**Figure 15:**
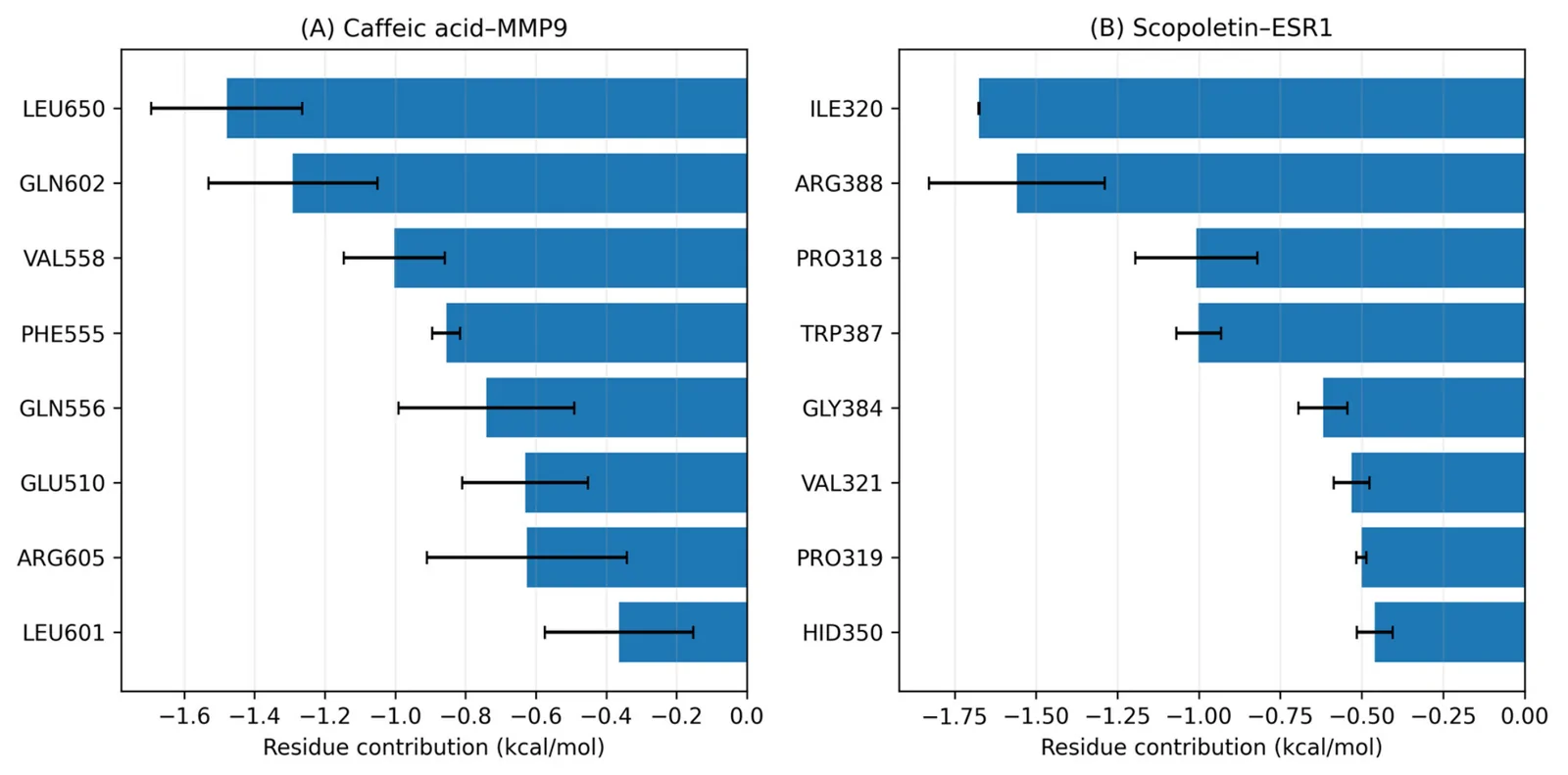
Replicate-level per-residue MM/GBSA energy decomposition for the caffeic acid–MMP9 (**A**) and scopoletin–ESR1 (**B**) complexes. Bars represent mean residue contributions across the three independent trajectories, and error bars indicate the corresponding standard deviations.

Per-residue energy decomposition identified recurrent favorable contributions within each complex (Figure 15). For caffeic acid–MMP9, the largest mean favorable contributions across the three runs were observed for LEU650 (−1.48 ± 0.22 kcal/mol), GLN602 (−1.29 ± 0.24 kcal/mol), VAL558 (−1.00 ± 0.14 kcal/mol), PHE555 (−0.85 ± 0.04 kcal/mol), GLN556 (−0.74 ± 0.25 kcal/mol), GLU510 (−0.63 ± 0.18 kcal/mol), and ARG605 (−0.63 ± 0.28 kcal/mol). For scopoletin–ESR1, ILE320 (−1.67 ± 0.00 kcal/mol), ARG388 (−1.56 ± 0.27 kcal/mol), PRO318 (−1.01 ± 0.19 kcal/mol), TRP387 (−1.00 ± 0.07 kcal/mol), GLY384 (−0.62 ± 0.08 kcal/mol), VAL321 (−0.53 ± 0.06 kcal/mol), PRO319 (−0.50 ± 0.02 kcal/mol), and HID350 (−0.46 ± 0.06 kcal/mol) showed the principal favorable residue-level contributions.

Overall, the three replicate-level MM/GBSA estimates were closely grouped within each ligand–target system, while the scopoletin–ESR1 complex showed a more negative mean ΔG_bind than the caffeic acid–MMP9 complex under the same calculation protocol.

## 4. Discussion

The present study constructed a chicken-oriented computational framework linking 38 reported candidate constituents of Fraxini Cortex with intestinal inflammation-related molecular features. The initial compound-related and disease-related datasets were obtained from predominantly human-oriented resources, yielding 170 shared human targets. These targets were subsequently mapped to 133 nonredundant *Gallus gallus* homologues before chicken-specific PPI and enrichment analyses. This cross-species reconstruction makes the downstream network more relevant to poultry biology and provides a more appropriate basis for interpreting the predicted targets and pathways in laying hens. At the same time, orthologue mapping reflects sequence and annotation correspondence rather than complete equivalence of tissue expression, regulatory context, or biological function. The resulting network is therefore best viewed as a chicken-oriented prediction framework that identifies molecular relationships worthy of subsequent experimental evaluation.

At the constituent level, caffeic acid, sinapyl alcohol, scopoletin, 2,4-dimethylphenol, nonan-1-ol, 1-octen-3-ol, sinapaldehyde, and several other compounds occupied highly connected positions in the compound-target network. Degree in this context describes the number of predicted network connections and is therefore a topological parameter rather than a measure of constituent concentration, intestinal exposure, binding affinity, or pharmacological potency. This distinction is particularly relevant for small volatile compounds such as nonan-1-ol and 1-octen-3-ol, because a highly connected predicted target profile may coexist with uncertain abundance or exposure in an actual botanical preparation. Caffeic acid and scopoletin were subsequently prioritized because they combined favorable network positions with representation in the docking and dynamic analyses. Previous mammalian studies have described anti-inflammatory or intestinal effects of scopoletin, related coumarins, and caffeic acid [25,26,27,28,29], providing biological context for these predicted associations. Poultry, however, differs from mammalian systems in gastrointestinal physiology, microbiota-mediated transformation, absorption, first-pass metabolism, tissue distribution, and clearance. In addition, the present study did not chemically quantify an authenticated Fraxini Cortex batch. Consequently, caffeic acid and scopoletin are more appropriately regarded as computationally prioritized reported constituents, while the potential contributions of volatile compounds and other matrix components remain part of the broader multi-component prediction.

Chicken-specific PPI analysis further refined the predicted target hierarchy. The *Gallus gallus* STRING network contained 117 non-isolated nodes and 683 interactions, and the combined cytoHubba-MCODE strategy retained 13 module-associated core candidate targets: BCL2, SRC, HIF1A, MMP9, ESR1, EGFR, ALB, TLR4, STAT1, PTGS2, SIRT1, JUN, and PPARG. BCL2, SRC, HIF1A, MMP9, ESR1, and EGFR showed the six highest Degree values within this final set and were used for subsequent docking. The fact that STAT3 ranked highly in the global PPI network but was not retained after module-based intersection illustrates the value of integrating several topological criteria rather than relying on one centrality metric. Functionally, the retained targets connect predicted processes involving epithelial survival, receptor-kinase signaling, extracellular-matrix remodeling, innate immune recognition, lipid-mediator biology, and transcriptional regulation. Experimental studies have linked SRC/PI3K signaling with epithelial migration and wound closure [30], BCL2-associated signaling with intestinal barrier responses in broiler chickens [31], MMP9 with epithelial tight-junction permeability [32], and HIF1A, SIRT1, and PPARG with intestinal epithelial adaptation or inflammatory regulation [33,34,35]. These findings provide biological context for the target classes identified computationally and suggest several candidate nodes for chicken-specific validation. Receptor selection for structural prioritization was made reproducible by applying the within-core upper-quartile threshold (Degree ≥ Q3 = 33) after the cytoHubba–MCODE consensus step, which yielded six receptors. This use of Degree is strictly topological and does not confer biological or therapeutic priority by itself.

The enrichment results indicate that the predicted target set is distributed across multiple functional systems rather than concentrated in a single signaling axis. After multiple-testing adjustment, 109 GO terms remained significant, including 78 biological process, 2 cellular component, and 29 molecular function terms. Highly ranked categories involved hormone responses, receptor tyrosine kinase signaling, regulation of kinase activity, defense responses, receptor complexes, and protein kinase-related functions. These annotations are consistent with the predicted involvement of EGFR, SRC, ESR1, HIF1A, SIRT1, and PPARG and suggest coordinated relationships among receptor-associated signaling, transcriptional adaptation, and epithelial homeostasis. KEGG analysis likewise identified 39 adjusted-significant pathways, including ErbB signaling, focal adhesion, C-type lectin receptor signaling, VEGF signaling, MAPK signaling, efferocytosis, FoxO signaling, Toll-like receptor signaling, NOD-like receptor signaling, apoptosis, adherens junction, arachidonic acid metabolism, autophagy, and mTOR signaling. Integration with the 13 final PPI targets showed relatively high core-target representation in NOD-like receptor signaling (4/12, 33.3%), C-type lectin receptor signaling (4/15, 26.7%), efferocytosis (4/16, 25.0%), Toll-like receptor signaling (3/12, 25.0%), ErbB signaling (3/15, 20.0%), and focal adhesion (4/21, 19.0%). Together, these predictions point to an interconnected molecular landscape involving innate immune sensing, epithelial adhesion, cell survival, damaged-cell clearance, and tissue remodeling.

Immune-related enrichment is particularly informative when considered in an avian context. Mammalian studies have linked epithelial NF-kappaB signaling, PI3K-Akt-associated barrier regulation, Th17-related immunity, and C-type lectin recognition with intestinal inflammation [36,37,38,39,40], whereas poultry studies provide species-relevant evidence that some of these biological themes are also active in chickens. In broilers challenged with necrotic enteritis, Astragalus polysaccharide supplementation was associated with changes in the intestinal Th17/Treg balance, inflammatory mediators, and barrier-related indices [41]. Chicken heterophils and peripheral blood mononuclear cells respond to the Dectin-1-selective agonist curdlan [42], indicating a functional beta-glucan-responsive immune phenotype in birds. Intestinal injury in broilers has also been associated with TLR4/TNF-alpha signaling during ammonia exposure and with TLR4/MyD88/NF-kappaB signaling during LPS challenge [43,44]. These poultry observations make the predicted innate-immune and inflammatory pathway relationships biologically plausible while also highlighting the need to interpret chicken immune signaling on its own species-specific basis. Similarly, statistically enriched pathways carrying cancer- or infection-related KEGG names are most appropriately understood through the shared genes that generate the annotation rather than through the disease label itself; their value lies in identifying conserved signaling modules that overlap with the chicken target set.

The integrated network further suggests that inflammatory sensing and epithelial repair-related processes may be connected through shared predicted targets. PTGS2 provides a useful example. Arachidonic acid metabolism was enriched and PTGS2 remained within the final PPI core set, but cyclooxygenase signaling in the intestine can participate in both inflammatory and reparative contexts. Eosinophil-derived COX-2/PGE2 signaling has been linked to an IL-22-associated mucosal repair response in experimental colitis [45]. In the present computational network, PTGS2 is therefore better interpreted as a candidate regulatory node connecting lipid-mediator metabolism with inflammatory and epithelial responses rather than as a simple predicted inhibitory target. The same systems-level interpretation applies to TLR4, STAT1, JUN, PPARG, SRC, EGFR, and MMP9. Their connections with MAPK, Toll-like receptor, C-type lectin receptor, FoxO, focal adhesion, VEGF, ErbB, apoptosis, autophagy, and related pathways suggest a multi-pathway architecture in which immune recognition, cell survival, adhesion, matrix remodeling, and epithelial recovery are computationally linked. This architecture is compatible with the multifactorial nature of intestinal inflammation in poultry and provides a set of testable pathway relationships without requiring a single pathway to dominate the predicted response.

Molecular docking provided a structural layer for prioritizing selected compound-target combinations. Across the 60 ligand-target pairs, AutoDock Vina scores ranged from −7.930 to −3.412 kcal/mol, with MMP9-scopoletin, MMP9-caffeic acid, SRC-scopoletin, ESR1-scopoletin, and MMP9-sinapaldehyde among the lowest-scoring combinations. Docking scores are approximate ranking functions whose relationship with experimental binding affinity varies among targets and protocols [46]. Accordingly, the present scores are most useful for comparing ligand-protein conformations generated under the same computational settings. The predicted poses also depend on receptor structure quality, protonation, pocket definition, grid placement, and the limited treatment of conformational flexibility. These considerations are particularly relevant for chicken proteins represented by predicted structures and for receptor-encompassing search boxes. The docking stage therefore serves as a structural prioritization step within the broader prediction workflow. The selection of caffeic acid-MMP9 and scopoletin-ESR1 for molecular dynamics also ensured representation of two distinct candidate constituents and two distinct core targets, allowing the dynamic analysis to include both caffeic acid and scopoletin rather than focusing on a single chemical class. A separately executed, fully specified rerun of all 60 combinations was used only as a reproducibility/sensitivity assessment and showed high concordance with the archived score matrix (Pearson r = 0.981; Spearman rho = 0.985; mean absolute difference = 0.119 kcal/mol), with caffeic acid-MMP9 and scopoletin-ESR1 remaining highly ranked. This concordance supports the stability of the comparative ranking while avoiding retrospective redefinition of undocumented settings in the original run. The local AlphaFold confidence audit further showed that 49/50 AlphaFold receptor–ligand best poses had a mean local pLDDT ≥ 70; the caffeic acid/caffeate-MMP9 and scopoletin-ESR1 sampled regions had mean local pLDDT values of 94.67 and 97.42, respectively. These confidence data support local structural order but do not establish physiological pocket identity, and docking remains exploratory structural prioritization. The ten-compound ligand panel is therefore described only as the archived docking subset. Because the original compound-selection rule was not preserved and the archived panel does not exactly match the revised network Degree > Q3 tier, we do not retrospectively label these ten ligands as the objectively highest-ranked or biologically most important constituents; omission of the other retained compounds is not interpreted as negative evidence.

The three independent 100 ns MD simulations for each selected complex provide a more informative view of conformational behavior than a single trajectory. For caffeic acid-MMP9, the three runs showed broadly comparable Rg, SASA, ligand RMSD, and RMSF profiles, with late-stage Rg and SASA values approaching similar ranges and the ligand RMSD remaining comparatively limited. Scopoletin-ESR1 also showed reproducible overall conformational evolution, although the three trajectories sampled a somewhat broader range of conformations. Independent biomolecular simulations are expected to explore partially different paths because small differences in starting conditions can redirect sampling across a rugged energy landscape; for this reason, replicate behavior is more informative than visual identity of individual traces [47,48]. The localized low-energy regions observed in the two-dimensional Gibbs free-energy landscapes and the six corresponding three-dimensional free-energy surfaces further indicate that the trajectories repeatedly sampled preferred conformational states. These MD results therefore add dynamic information to the docking predictions and identify both caffeic acid-MMP9 and scopoletin-ESR1 as structurally interesting systems for subsequent experimental binding or target-engagement studies.

The endpoint energy calculations provide an additional comparative descriptor of these two simulated systems. Using the same MM/GBSA protocol for all three independent trajectories, caffeic acid-MMP9 showed a mean predicted binding energy of −19.92 ± 0.30 kcal/mol, whereas scopoletin-ESR1 showed −21.53 ± 0.32 kcal/mol. The close grouping of the three replicate estimates within each system indicates good reproducibility of the endpoint calculation under the selected simulation and solvation settings. Under the same protocol, the more negative value for scopoletin-ESR1 suggests a comparatively favorable predicted energetic state. MM/PBSA and MM/GBSA calculations are widely used for post-processing MD trajectories, but their numerical values depend on conformational sampling, force-field parameters, continuum-solvation treatment, dielectric settings, and entropy handling [49,50]. The gmx_MMPBSA framework enables consistent end-state calculations and residue-level decomposition from GROMACS trajectories [51]. In the present analysis, favorable residue-level contributions were predicted for LEU650, GLN602, VAL558, PHE555, GLN556, GLU510, and ARG605 in the caffeic acid-MMP9 system and for ILE320, ARG388, PRO318, TRP387, GLY384, VAL321, PRO319, and HID350 in the scopoletin-ESR1 system. These residues represent predicted interaction hotspots that can guide future mutational, biochemical, or structural evaluation.

Overall, the combined network, enrichment, docking, MD, and MM/GBSA results suggest that Fraxini Cortex may be associated with a distributed set of molecular relationships involving epithelial survival and repair, receptor and kinase signaling, innate immune recognition, cell adhesion, extracellular-matrix remodeling, and lipid-mediator pathways in the chicken-oriented computational model. Caffeic acid and scopoletin emerge as prioritized reported constituents, while MMP9, ESR1, SRC, EGFR, BCL2, HIF1A, TLR4, PTGS2, SIRT1, JUN, PPARG, STAT1, and related targets define a broader predicted molecular network. The computational nature of these associations is important for their interpretation: database-derived target prediction, orthologue mapping, enrichment, docking, and molecular simulation each contribute a different level of prediction, and their convergence is most useful for selecting hypotheses for experimental testing. A logical next stage would combine authenticated Fraxini Cortex chemical profiling with chicken-specific exposure assessment, followed by intestinal epithelial-cell or organoid experiments examining barrier function, inflammatory mediators, target expression or phosphorylation, and direct target engagement. Chicken intestinal organoids have already been used to study feed-additive effects on innate immune and kinase-related responses [52], making them a particularly suitable intermediate platform before controlled laying-hen studies. Subsequent work should also define effective exposure, metabolism, safety, toxicity, potential egg residues, batch variability, and botanical standardization so that the computational predictions can be evaluated in a physiologically and production-relevant context.

## 5. Conclusions

This entirely in silico study constructed a chicken-oriented computational framework to explore candidate molecular relationships between reported Fraxini Cortex constituents and intestinal inflammation in laying hens. Thirty-eight candidate constituents were retained, among which caffeic acid and scopoletin were computationally prioritized through network and structural analyses. The 170 shared human targets were mapped to 133 nonredundant *Gallus gallus* homologues, and chicken-specific PPI analysis identified 13 module-supported core candidate targets, including BCL2, SRC, HIF1A, MMP9, ESR1, EGFR, ALB, TLR4, STAT1, PTGS2, SIRT1, JUN, and PPARG. GO and KEGG enrichment associated these targets with receptor and kinase signaling, innate immune recognition, epithelial adhesion and survival, extracellular-matrix remodeling, lipid-mediator metabolism, apoptosis, autophagy, and related processes. Molecular docking further prioritized selected ligand–target combinations, while three independent 100 ns molecular dynamics simulations of the caffeic acid–MMP9 and scopoletin–ESR1 complexes showed broadly reproducible conformational behavior across replicate trajectories. MM/GBSA calculations yielded mean endpoint binding-energy estimates of −19.92 ± 0.30 kcal/mol for caffeic acid–MMP9 and −21.53 ± 0.32 kcal/mol for scopoletin–ESR1. These findings identify candidate constituent–target–pathway relationships for further investigation rather than establishing biological efficacy or a mechanism of action in laying hens. Before biological efficacy or mechanistic relevance can be established, these computational predictions require experimental validation using authenticated and chemically characterized Fraxini Cortex material, chicken intestinal cells or organoids, direct target-engagement assays, and appropriately controlled studies in laying hens. Poultry-specific exposure, metabolism, effective dose, safety, toxicity, egg residues, batch variability, and botanical standardization also require further evaluation before practical application can be considered.

## Data Availability

The original contributions presented in this study are included in the article and its Supplementary Materials. Further inquiries can be directed to the corresponding authors.

## Supplementary Materials

The following supporting information can be downloaded at: https://www.mdpi.com/article/10.3390/vetsci13090874/s1.
