# Supplementary material for: Network Pharmacology, Molecular Docking, and Molecular Dynamics Simulation to Explore Predicted Molecular Associations Between Fraxini Cortex Constituents and Intestinal Inflammation in Laying Hens
Source: Vet Sci. 2026 Aug 27;13(9):874. doi: 10.3390/vetsci13090874 (PMC13611608; doi:10.3390/vetsci13090874)
Supplement: Supplementary file 1 [file vetsci-13-00874-s001.zip › Supplementary/Supplementary S1.pdf]

Supplementary Data S1

Complete Screening Audit for 91 Initial Candidate Records, Provenance Information for the Retained Fraxini Cortex Candidate Compounds, and Network-Topology Information

Revised reproducible workflow: 91 merged candidate records were standardized to 85 compounds with valid chemical identities; 50 showed High predicted gastrointestinal (GI) absorption, and 38 met the predefined SwissADME drug-likeness criterion. The retention rule was GI absorption = High AND at least two of the five SwissADME drug-likeness filters (Lipinski, Ghose, Veber, Egan, and Muegge) showing zero violations. SwissTargetPrediction (score > 0.1) subsequently yielded 528 nonredundant potential targets.

Chemical-provenance note: No physical Fraxini Cortex batch, extract, or formulation was chemically analyzed in this study. Evidence/source entries below indicate published analytical or isolation evidence, curated phytochemical records, or archived profile-source evidence only; they do not constitute batch-specific HPLC-MS/GC-MS confirmation or quantitative abundance data for study material.

Expanded reproducibility audit added in this revision: Table S1A now represents all 91 initial candidate records. The 53 newly added records comprise 6 entries excluded before SwissADME because a valid PubChem CID/usable chemical identity was not recovered, 35 compounds excluded because predicted GI absorption was Low, and 12 High-GI compounds excluded because fewer than two of the five drug-likeness filters showed zero violations.

Table S1A. Complete screening audit of all 91 initial Fraxini Cortex candidate records (38 retained records plus 53 excluded/unresolved records).

No.	Compound	Chemical class	PubChem CID	CAS No.	Canonical SMILES	Provenance evidence / source	SwissADME screening profile	Screening decision / provenance interpretation
1	Furfural	Furan aldehyde	7362	98-01-1	O=Cc1ccco1	C: Fraxini Cortex volatile/profile dataset. Volatile/chemical-profile support [S4,S8]; compound-level primary-table match was not independently reverified.	GI High; L/G/V/E/M = 0/3/0/0/1; 3/5 filters with 0 violations	Retained; lower botanical confidence.
2	Steroidal glycoside (archived CID 11968950)	Steroidal glycoside	11968950	Not assigned in archived dataset	CC[C@H](/C=C/C(C)C1CCC2[C@@]1(CCC3C2=CCC4[C@@]3(CC[C@@H](C4)O[C@H]5[C@@H]([C@H]([C@@H]([C@H](O5)CO)O)O)O)C)C)C(C)C	D: Source not independently confirmed. A Fraxini-specific primary source was not confirmed during the revision audit; the exact archived computational input is retained by PubChem CID 11968950.	GI High; L/G/V/E/M = 1/4/0/0/1; 2/5 filters with 0 violations	Retained for workflow reproducibility; provenance requires caution.
3	Palmitic acid	Saturated fatty acid	985	57-10-3	CCCCCCCCCCCCCCCC(=O)O	C: Fraxini Cortex volatile/profile dataset. Volatile/chemical-profile support [S4,S8]; compound-level primary-table match was not independently reverified.	GI High; L/G/V/E/M = 1/0/1/0/1; 2/5 filters with 0 violations	Retained; lower botanical confidence.
4	Fraxetin	Coumarin	5273569	574-84-5	COc1cc2ccc(=O)oc2c(O)c1O	A: Fraxini Cortex / Fraxinus bark. Direct analytical evidence; HPLC-MS studies [S2,S3]	GI High; L/G/V/E/M = 0/0/0/0/0; 5/5 filters with 0 violations	Retained; chemically supported candidate constituent.
5	Benzaldehyde	Aromatic aldehyde	240	100-52-7	O=Cc1ccccc1	C: Fraxini Cortex volatile/profile dataset. Volatile/chemical-profile support [S4,S8]; compound-level primary-table match was not independently reverified.	GI High; L/G/V/E/M = 0/3/0/0/2; 3/5 filters with 0 violations	Retained; lower botanical confidence.
6	N-Phenyl-2-naphthylamine	Aromatic amine	8679	135-88-6	c1ccc(Nc2ccc3ccccc3c2)cc1	A: Fraxinus bark (historical report). Historical phytochemical evidence [S7]	GI High; L/G/V/E/M = 1/0/0/0/1; 3/5 filters with 0 violations	Retained; chemically supported candidate constituent.
7	2-Nonenal	Unsaturated aldehyde	5283335	18829-56-6	CCCCCC/C=C/C=O	C: Fraxini Cortex volatile/profile dataset. Volatile/chemical-profile support [S4,S8]; compound-level primary-table match was not independently reverified.	GI High; L/G/V/E/M = 0/1/0/0/2; 3/5 filters with 0 violations	Retained; lower botanical confidence.
8	Isoamyl alcohol	Aliphatic alcohol	31260	123-51-3	CC(C)CCO	C: Fraxini Cortex volatile/profile dataset. Volatile/chemical-profile support [S4,S8]; compound-level primary-table match was not independently reverified.	GI High; L/G/V/E/M = 0/3/0/0/2; 3/5 filters with 0 violations	Retained; lower botanical confidence.
9	Methyl tetradecanoate	Fatty acid methyl ester	31284	124-10-7	CCCCCCCCCCCCCC(=O)OC	C: Fraxini Cortex volatile/profile dataset. Volatile/chemical-profile support [S4,S8]; compound-level primary-table match was not independently reverified.	GI High; L/G/V/E/M = 0/0/1/0/1; 3/5 filters with 0 violations	Retained; lower botanical confidence.
10	Scopoletin	Coumarin	5280460	92-61-5	COc1cc2ccc(=O)oc2cc1O	A: Fraxini Cortex / Fraxinus bark. Direct analytical evidence; Cortex Fraxini determination [S1,S3]	GI High; L/G/V/E/M = 0/0/0/0/1; 4/5 filters with 0 violations	Retained; chemically supported candidate constituent.
11	Benzyl alcohol	Aromatic alcohol	244	100-51-6	OCc1ccccc1	C: Fraxini Cortex volatile/profile dataset. Volatile/chemical-profile support [S4,S8]; compound-level primary-table match was not independently reverified.	GI High; L/G/V/E/M = 0/3/0/0/2; 3/5 filters with 0 violations	Retained; lower botanical confidence.
12	2,3-Butanediol	Aliphatic diol	262	513-85-9	CC(O)C(C)O	C: Fraxini Cortex volatile/profile dataset. Volatile/chemical-profile support [S4,S8]; compound-level primary-table match was not independently reverified.	GI High; L/G/V/E/M = 0/3/0/0/2; 3/5 filters with 0 violations	Retained; lower botanical confidence.
13	Geranylacetone	Terpenoid ketone	1549778	3796-70-1	CC(=O)CC/C=C(\C)CCC=C(C)C	C: Fraxini Cortex volatile/profile dataset. Volatile/chemical-profile support [S4,S8]; compound-level primary-table match was not independently reverified.	GI High; L/G/V/E/M = 0/0/0/0/2; 4/5 filters with 0 violations	Retained; lower botanical confidence.
14	2,4-Dimethylphenol	Alkylphenol	7771	105-67-9	Cc1ccc(O)c(C)c1	C: Fraxini Cortex volatile/profile dataset. Volatile/chemical-profile support [S4,S8]; compound-level primary-table match was not independently reverified.	GI High; L/G/V/E/M = 0/3/0/0/2; 3/5 filters with 0 violations	Retained; lower botanical confidence.

No.	Compound	Chemical class	PubChem CID	CAS No.	Canonical SMILES	Provenance evidence / source	SwissADME screening profile	Screening decision / provenance interpretation
15	1-Octen-3-ol	Volatile unsaturated alcohol	18827	3391-86-4	C=CC(O)CCCCC	B: Fraxini Cortex database record. Curated Qin Pi/Fraxini Cortex database support [S9]	GI High; L/G/V/E/M = 0/1/0/0/2; 3/5 filters with 0 violations	Retained; database-supported candidate constituent.
16	Methyl pentadecanoate	Fatty acid methyl ester	23518	7132-64-1	CCCCCCCCCCCCCCC(=O)OC	C: Fraxini Cortex volatile/profile dataset. Volatile/chemical-profile support [S4,S8]; compound-level primary-table match was not independently reverified.	GI High; L/G/V/E/M = 1/0/1/0/1; 2/5 filters with 0 violations	Retained; lower botanical confidence.
17	Nonanal	Aliphatic aldehyde	31289	124-19-6	CCCCCCCCC=O	C: Fraxini Cortex volatile/profile dataset. Volatile/chemical-profile support [S4,S8]; compound-level primary-table match was not independently reverified.	GI High; L/G/V/E/M = 0/1/0/0/2; 3/5 filters with 0 violations	Retained; lower botanical confidence.
18	Sinapyl alcohol	Phenylpropanoid	5280507	537-33-7	COc1cc(/C=C/CO)cc(OC)c1O	B: Fraxini Cortex database/literature record. TCMSP / literature-database support [S10]	GI High; L/G/V/E/M = 0/0/0/0/0; 5/5 filters with 0 violations	Retained; database-supported candidate constituent.
19	Nonan-1-ol	Volatile alcohol	8914	143-08-8	CCCCCCCCCO	B: Fraxini Cortex database record. Curated Qin Pi/Fraxini Cortex database support [S9]	GI High; L/G/V/E/M = 0/1/0/0/2; 3/5 filters with 0 violations	Retained; database-supported candidate constituent.
20	Dibenzofuran	Dibenzofuran	568	132-64-9	c1ccc2c(c1)oc1ccccc12	C: Fraxini Cortex volatile/profile dataset. Volatile/chemical-profile support [S4,S8]; compound-level primary-table match was not independently reverified.	GI High; L/G/V/E/M = 0/0/0/0/2; 4/5 filters with 0 violations	Retained; lower botanical confidence.
21	2-Pentadecanone	Aliphatic ketone	61303	2345-28-0	CCCCCCCCCCCCCC(C)=O	C: Fraxini Cortex volatile/profile dataset. Volatile/chemical-profile support [S4,S8]; compound-level primary-table match was not independently reverified.	GI High; L/G/V/E/M = 0/0/1/0/2; 3/5 filters with 0 violations	Retained; lower botanical confidence.
22	2,6-Dimethoxy-1,4-benzoquinone	Benzoquinone	68262	530-55-2	COC1=CC(=O)C=C(OC)C1=O	A: Fraxinus bark (historical report). Historical phytochemical evidence [S7]	GI High; L/G/V/E/M = 0/0/0/0/1; 4/5 filters with 0 violations	Retained; chemically supported candidate constituent.
23	Diacetone alcohol	Hydroxy ketone	31256	123-42-2	CC(=O)CC(C)(C)O	C: Fraxini Cortex volatile/profile dataset. Volatile/chemical-profile support [S4,S8]; compound-level primary-table match was not independently reverified.	GI High; L/G/V/E/M = 0/2/0/0/1; 3/5 filters with 0 violations	Retained; lower botanical confidence.
24	Caffeic acid	Phenolic acid	689043	331-39-5	O=C(O)/C=C/c1ccc(O)c(O)c1	A: Fraxini Cortex / F. chinensis bark. Direct analytical/isolation evidence [S1,S5]	GI High; L/G/V/E/M = 0/0/0/0/1; 4/5 filters with 0 violations	Retained; chemically supported candidate constituent.
25	(S)-Acetoin	Hydroxy ketone	447765	78183-56-9	CC(=O)[C@H](C)O	C: Fraxini Cortex volatile/profile dataset. Volatile/chemical-profile support [S4,S8]; compound-level primary-table match was not independently reverified.	GI High; L/G/V/E/M = 0/3/0/0/2; 3/5 filters with 0 violations	Retained; lower botanical confidence.
26	Plaunotol	Diterpenoid alcohol	5282197	64218-02-6	CC(C)=CCC/C(C)=C/CC/C(=C/CC/C(C)=C/CO)CO	D: Source not independently confirmed. Fraxini-specific primary source was not confirmed during the revision audit; retained because it is present in the recalculated 38-compound source dataset.	GI High; L/G/V/E/M = 0/0/1/0/1; 3/5 filters with 0 violations	Retained for workflow reproducibility; provenance requires caution.
27	Sinapaldehyde	Phenylpropanoid aldehyde	5280802	4206-58-0	COc1cc(/C=C/C=O)cc(OC)c1O	A: F. chinensis bark. Direct isolation evidence [S5]	GI High; L/G/V/E/M = 0/0/0/0/0; 5/5 filters with 0 violations	Retained; chemically supported candidate constituent.
28	2-(4-Hydroxyphenyl)ethanol (tyrosol)	Phenolic alcohol	10393	501-94-0	OCCc1ccc(O)cc1	A: Fraxini Cortex / F. chinensis bark. Direct isolation evidence [S5,S6]	GI High; L/G/V/E/M = 0/2/0/0/1; 3/5 filters with 0 violations	Retained; chemically supported candidate constituent.
29	L-Citrulline (citrulline)	Amino acid	9750	372-75-8	NC(=O)NCCC[C@H](N)C(=O)O	D: Source not independently confirmed. A sufficiently traceable Fraxini-specific primary source was not confirmed during the revision audit.	GI High; L/G/V/E/M = 0/1/0/0/2; 3/5 filters with 0 violations	Retained for workflow reproducibility; provenance requires caution.
30	8-Hydroxypinoresinol	Lignan	3010930	81426-17-7	COc1cc([C@H]2OC[C@]3(O)[C@@H](c4ccc(O)c(OC)c4)OC[C@H]23)ccc1O	A: Authenticated Cortex Fraxini (F. chinensis). Direct isolation evidence [S6]	GI High; L/G/V/E/M = 0/0/0/0/0; 5/5 filters with 0 violations	Retained; chemically supported candidate constituent.
31	1-Hexanol	Volatile alcohol	8103	111-27-3	CCCCCCO	B: Fraxini Cortex database record. Curated Qin Pi/Fraxini Cortex database support [S9]	GI High; L/G/V/E/M = 0/2/0/0/2; 3/5 filters with 0 violations	Retained; database-supported candidate constituent.
32	Gamma-aminobutyric acid	Non-proteinogenic amino acid	119	56-12-2	NCCCC(=O)O	B: Fraxini Cortex database record. Curated Qin Pi/Fraxini Cortex database support [S9]	GI High; L/G/V/E/M = 0/3/0/0/3; 3/5 filters with 0 violations	Retained; database-supported candidate constituent.
33	2-Acetylfuran	Furan ketone	14505	1192-62-7	CC(=O)c1ccco1	C: Fraxini Cortex volatile/profile dataset. Volatile/chemical-profile support [S4,S8]; compound-level primary-table match was not independently reverified.	GI High; L/G/V/E/M = 0/3/0/0/1; 3/5 filters with 0 violations	Retained; lower botanical confidence.

No.	Compound	Chemical class	PubChem CID	CAS No.	Canonical SMILES	Provenance evidence / source	SwissADME screening profile	Screening decision / provenance interpretation
34	Esculetin	Coumarin	5281416	305-01-1	O=c1ccc2cc(O)c(O)cc2o1	A: Fraxini Cortex. Direct analytical evidence [S1,S2,S3]	GI High; L/G/V/E/M = 0/1/0/0/1; 3/5 filters with 0 violations	Retained; chemically supported candidate constituent.
35	Cedrenol	Sesquiterpenoid alcohol	119831	28231-03-0	C=C1C(O)CC23CC1C(C)(C)C2CCC3C	C: Fraxini Cortex volatile/profile dataset. Volatile/chemical-profile support [S4,S8]; compound-level primary-table match was not independently reverified.	GI High; L/G/V/E/M = 0/0/0/0/1; 4/5 filters with 0 violations	Retained; lower botanical confidence.
36	Syringaldehyde	Phenolic aldehyde	8655	134-96-3	COc1cc(C=O)cc(OC)c1O	A: F. chinensis bark. Direct isolation evidence [S5]	GI High; L/G/V/E/M = 0/0/0/0/1; 4/5 filters with 0 violations	Retained; chemically supported candidate constituent.
37	4-Methyldibenzofuran	Methylated dibenzofuran	43489	7320-53-8	Cc1cccc2c1oc1ccccc12	C: Fraxini Cortex volatile/profile dataset. Volatile/chemical-profile support [S4,S8]; compound-level primary-table match was not independently reverified.	GI High; L/G/V/E/M = 0/0/0/0/2; 4/5 filters with 0 violations	Retained; lower botanical confidence.
38	Dibutyl phthalate	Phthalate ester	3026	84-74-2	CCCCOC(=O)c1ccccc1C(=O)OCCCC	D: Botanical provenance not confirmed. Compound identity is valid, but Fraxini-specific botanical provenance was not confirmed; possible exogenous/analytical signal.	GI High; L/G/V/E/M = 0/0/0/0/0; 5/5 filters with 0 violations	Retained for workflow reproducibility; provenance requires caution.
39	Syringin	-	5316860	118-34-3	COC1=CC(=CC(=C1OC2C(C(C(C(O2)CO)O)O)O)OC)C=CCO	Archived record n4; TCMSP Qin Pi (Cortex Fraxini), MOL000347.	GI Low; L/G/V/E/M = 0/1/0/1/0; 3/5 filters with 0 violations.	Excluded at GI-absorption stage: SwissADME predicted gastrointestinal absorption = Low.
40	Sitogluside	-	5742590	474-58-8	CCC(CCC(C)C1CCC2C1(CCC3C2CC=C4C3(CCC(C4)OC5C(C(C(C(O5)CO)O)O)O)C)C)C(C)C	Archived record n5; TCMSP Qin Pi (Cortex Fraxini), MOL000357.	GI Low; L/G/V/E/M = 1/4/0/0/1; 2/5 filters with 0 violations.	Excluded at GI-absorption stage: SwissADME predicted gastrointestinal absorption = Low.
41	Beta-Sitosterol	-	222284	83-46-5	CCC(CCC(C)C1CCC2C1(CCC3C2CC=C4C3(CCC(C4)O)C)C)C(C)C	Archived record n6; TCMSP Qin Pi (Cortex Fraxini), MOL000358.	GI Low; L/G/V/E/M = 1/3/0/1/2; 1/5 filters with 0 violations.	Excluded at GI-absorption stage: SwissADME predicted gastrointestinal absorption = Low.
42	Ursolic Acid	-	64945	77-52-1	CC1CCC2(CCC3(C(=CCC4C3(CCC5C4(CCC(C5(C)C)O)C)C)C2C1C)C)C(=O)O	Archived record n7; TCMSP Qin Pi (Cortex Fraxini), MOL000511.	GI Low; L/G/V/E/M = 1/3/0/1/1; 1/5 filters with 0 violations.	Excluded at GI-absorption stage: SwissADME predicted gastrointestinal absorption = Low.
43	Triacontanoic acid	-	10471	506-50-3	CCCCCCCCCCCCCCCCCCCCCCCCCCCCCC(=O)O	Archived record n8; TCMSP Qin Pi (Cortex Fraxini), MOL000515.	GI Low; L/G/V/E/M = 1/3/1/1/2; 0/5 filters with 0 violations.	Excluded at GI-absorption stage: SwissADME predicted gastrointestinal absorption = Low.
44	Esculin	-	5281417	531-75-9	C1=CC(=O)OC2=CC(=C(C=C21)OC3C(C(C(C(O3)CO)O)O)O)O	Archived record n15; TCMSP Qin Pi (Cortex Fraxini), MOL004456.	GI Low; L/G/V/E/M = 0/1/1/1/1; 1/5 filters with 0 violations.	Excluded at GI-absorption stage: SwissADME predicted gastrointestinal absorption = Low.
45	(2S,3R,4S,5S,6R)-2-[4-[(3R,3aS,6S,6aR)-3a-hydroxy-6-(4-hydroxy-3-methoxyphenyl)-3,4,6,6a-tetrahydro-1H-furo[3,4-c]furan-3-yl]-2-methoxyphenoxy]-6-(hydroxymethyl)oxane-3,4,5-triol	-	14756314	-	COC1=C(C=CC(=C1)C2C3COC(C3(CO2)O)C4=CC(=C(C=C4)OC5C(C(C(C(O5)CO)O)O)O)OC)O	Archived record n18; TCMSP Qin Pi (Cortex Fraxini), MOL006708.	GI Low; L/G/V/E/M = 3/2/1/1/3; 0/5 filters with 0 violations.	Excluded at GI-absorption stage: SwissADME predicted gastrointestinal absorption = Low.
46	Fraxin	-	5273568	524-30-1	COC1=C(C(=C2C(=C1)C=CC(=O)O2)OC3C(C(C(C(O3)CO)O)O)O)O	Archived record n20; TCMSP Qin Pi (Cortex Fraxini), MOL006710.	GI Low; L/G/V/E/M = 0/1/1/1/2; 1/5 filters with 0 violations.	Excluded at GI-absorption stage: SwissADME predicted gastrointestinal absorption = Low.
47	Osmanthuside H	-	192437	149155-70-4	C1C(C(C(O1)OCC2C(C(C(C(O2)OCCC3=CC=C(C=C3)O)O)O)O)O)(CO)O	Archived record n21; TCMSP Qin Pi (Cortex Fraxini), MOL006711.	GI Low; L/G/V/E/M = 2/1/1/1/4; 0/5 filters with 0 violations.	Excluded at GI-absorption stage: SwissADME predicted gastrointestinal absorption = Low.
48	Sinapaldehyde glucoside	-	25791064	154461-65-1	COC1=CC(=CC(=C1OC2C(C(C(C(O2)CO)O)O)O)OC)C=CC=O	Archived record n22; TCMSP Qin Pi (Cortex Fraxini), MOL006712.	GI Low; L/G/V/E/M = 0/1/0/1/0; 3/5 filters with 0 violations.	Excluded at GI-absorption stage: SwissADME predicted gastrointestinal absorption = Low.
49	1,5-Dimethylnaphthalene	-	11306	571-61-9	CC1=C2C=CC=C(C2=CC=C1)C	Archived record n23 in the merged Fraxini Cortex candidate dataset (chems_add/chems_tiqu); source-level botanical provenance was not recoverable from the archived screening files.	GI Low; L/G/V/E/M = 1/1/0/0/2; 2/5 filters with 0 violations.	Excluded at GI-absorption stage: SwissADME predicted gastrointestinal absorption = Low.
50	[(1S,4S,5R,6R,9S,14R)-4,5,6-trihydroxy-3,11,11,14-tetramethyl-15-oxo-7-tetracyclo[7.5.1.01,5.010,12]pentadeca-2,7-dienyl]methyl hexadecanoate	-	5318035	-	CCCCCCCCCCCCCCCC(=O)OCC1=CC2C3C(C3(C)C)CC(C4(C2=O)C=C(C(C4(C1O)O)O)C)C	Archived record n27 in the merged Fraxini Cortex candidate dataset (chems_add/chems_tiqu); source-level botanical provenance was not recoverable from the archived screening files.	GI Low; L/G/V/E/M = 2/4/1/1/2; 0/5 filters with 0 violations.	Excluded at GI-absorption stage: SwissADME predicted gastrointestinal absorption = Low.

No.	Compound	Chemical class	PubChem CID	CAS No.	Canonical SMILES	Provenance evidence / source	SwissADME screening profile	Screening decision / provenance interpretation
51	2-Methylnaphthalene	-	7055	91-57-6	CC1=CC2=CC=CC=C2C=C1	Archived record n30 in the merged Fraxini Cortex candidate dataset (chems_add/chems_tiqu); source-level botanical provenance was not recoverable from the archived screening files.	GI Low; L/G/V/E/M = 1/1/0/0/2; 2/5 filters with 0 violations.	Excluded at GI-absorption stage: SwissADME predicted gastrointestinal absorption = Low.
52	3-Methylphenanthrene	-	13258	832-71-3	CC1=CC2=C(C=C1)C=CC3=CC=CC=C32	Archived record n34 in the merged Fraxini Cortex candidate dataset (chems_add/chems_tiqu); source-level botanical provenance was not recoverable from the archived screening files.	GI Low; L/G/V/E/M = 1/0/0/0/3; 3/5 filters with 0 violations.	Excluded at GI-absorption stage: SwissADME predicted gastrointestinal absorption = Low.
53	Schottenol glucoside	-	124879	61376-86-1	CCC(CCC(C)C1CCC2C1(CCC3C2=CCC4C3(CCC(C4)OC5C(C(C(C(O5)CO)O)O)O)C)C)C(C)C	Archived record n36 in the merged Fraxini Cortex candidate dataset (chems_add/chems_tiqu); source-level botanical provenance was not recoverable from the archived screening files.	GI Low; L/G/V/E/M = 1/4/0/0/1; 2/5 filters with 0 violations.	Excluded at GI-absorption stage: SwissADME predicted gastrointestinal absorption = Low.
54	ethyl (9E,12E)-octadeca-9,12-dienoate	-	5365672	6114-21-2	CCCCCC=CCC=CCCCCCCCC(=O)OCC	Archived record n37 in the merged Fraxini Cortex candidate dataset (chems_add/chems_tiqu); source-level botanical provenance was not recoverable from the archived screening files.	GI High; L/G/V/E/M = 1/1/1/1/2; 0/5 filters with 0 violations.	Excluded at drug-likeness stage: GI = High, but only 0/5 filters showed zero violations (<2/5 required).
55	Acenaphthylene	-	9161	208-96-8	C1=CC2=C3C(=C1)C=CC3=CC=C2	Archived record n38 in the merged Fraxini Cortex candidate dataset (chems_add/chems_tiqu); source-level botanical provenance was not recoverable from the archived screening files.	GI Low; L/G/V/E/M = 1/1/0/0/2; 2/5 filters with 0 violations.	Excluded at GI-absorption stage: SwissADME predicted gastrointestinal absorption = Low.
56	AeculetinAeculetin	-	-	-	-	Archived raw record n40 in the merged Fraxini Cortex candidate dataset; the entry could not be standardized to a discrete PubChem chemical identity.	Not evaluated in SwissADME (no valid PubChem CID/usable standardized chemical structure).	Excluded before SwissADME: unresolved/non-standardized record; no valid PubChem CID or usable chemical identity was recovered.
57	AescuktinAescuktin	-	-	-	-	Archived raw record n41 in the merged Fraxini Cortex candidate dataset; the entry could not be standardized to a discrete PubChem chemical identity.	Not evaluated in SwissADME (no valid PubChem CID/usable standardized chemical structure).	Excluded before SwissADME: unresolved/non-standardized record; no valid PubChem CID or usable chemical identity was recovered.
58	alpha-(Hydroxymethyl)serine	-	439893	17149-11-0	C(C(CO)(C(=O)O)N)O	Archived record n42 in the merged Fraxini Cortex candidate dataset (chems_add/chems_tiqu); source-level botanical provenance was not recoverable from the archived screening files.	GI Low; L/G/V/E/M = 0/4/0/0/3; 3/5 filters with 0 violations.	Excluded at GI-absorption stage: SwissADME predicted gastrointestinal absorption = Low.
59	Dihydromyricetin	-	161557	27200-12-0	C1=C(C=C(C(=C1O)O)O)C2C(C(=O)C3=C(C=C(C=C3O2)O)O)O	Archived record n44 in the merged Fraxini Cortex candidate dataset (chems_add/chems_tiqu); source-level botanical provenance was not recoverable from the archived screening files.	GI Low; L/G/V/E/M = 1/0/1/1/1; 1/5 filters with 0 violations.	Excluded at GI-absorption stage: SwissADME predicted gastrointestinal absorption = Low.
60	(2s,4As,6as,8ar,10s,12as,14as,14br)-10-hydroxy-2,4a,6a,9,9,12a,14a-heptamethyl-1,2,3,4,4a,5,6,6a,7,8,8a,9,10,11,12,12a,13,14,14a,14b-icosahydropicene-2-carboxylic acid	-	159970	24480-45-3	CC1(C2CCC3=C(C2(CCC1O)C)CCC4(C3(CCC5(C4CC(CC5)(C)C(=O)O)C)C)C)C	Archived record n46 in the merged Fraxini Cortex candidate dataset (chems_add/chems_tiqu); source-level botanical provenance was not recoverable from the archived screening files.	GI Low; L/G/V/E/M = 1/3/0/1/1; 1/5 filters with 0 violations.	Excluded at GI-absorption stage: SwissADME predicted gastrointestinal absorption = Low.
61	Cichoriin	-	442101	531-58-8	C1=CC(=O)OC2=CC(=C(C=C21)O)OC3C(C(C(C(O3)CO)O)O)O	Archived record n48 in the merged Fraxini Cortex candidate dataset (chems_add/chems_tiqu); source-level botanical provenance was not recoverable from the archived screening files.	GI Low; L/G/V/E/M = 0/1/1/1/1; 1/5 filters with 0 violations.	Excluded at GI-absorption stage: SwissADME predicted gastrointestinal absorption = Low.
62	(2S,3R,4S,5S,6R)-2-[4-[(1S,2R)-1,3-dihydroxy-2-[4-[(E)-3-hydroxyprop-1-enyl]-2,6-dimethoxyphenoxy]propyl]-2-methoxyphenoxy]-6-(hydroxymethyl)oxane-3,4,5-triol	-	156594363	105279-10-5	COC1=CC(=CC(=C1OC(CO)C(C2=CC(=C(C=C2)OC3C(C(C(C(O3)CO)O)O)O)OC)O)OC)C=CCO	Archived record n50 in the merged Fraxini Cortex candidate dataset (chems_add/chems_tiqu); source-level botanical provenance was not recoverable from the archived screening files.	GI Low; L/G/V/E/M = 3/4/2/1/3; 0/5 filters with 0 violations.	Excluded at GI-absorption stage: SwissADME predicted gastrointestinal absorption = Low.
63	(9R,10S,13R,14R,17R)-17-[(2R,5S)-5-ethyl-6-methylheptan-2-yl]-10,13-dimethyl-1,2,4,5,6,9,11,12,14,15,16,17-dodecahydrocyclopenta[a]phenanthren-3-one	-	3082125	-	CCC(CCC(C)C1CCC2C1(CCC3C2=CCC4C3(CCC(=O)C4)C)C)C(C)C	Archived record n51 in the merged Fraxini Cortex candidate dataset (chems_add/chems_tiqu); source-level botanical provenance was not recoverable from the archived screening files.	GI Low; L/G/V/E/M = 1/3/0/1/2; 1/5 filters with 0 violations.	Excluded at GI-absorption stage: SwissADME predicted gastrointestinal absorption = Low.
64	Linoleic Acid	-	5280450	60-33-3	CCCCCC=CCC=CCCCCCCCC(=O)O	Archived record n54 in the merged Fraxini Cortex candidate dataset (chems_add/chems_tiqu); source-level botanical provenance was not recoverable from the archived screening files.	GI High; L/G/V/E/M = 1/1/1/1/1; 0/5 filters with 0 violations.	Excluded at drug-likeness stage: GI = High, but only 0/5 filters showed zero violations (<2/5 required).

No.	Compound	Chemical class	PubChem CID	CAS No.	Canonical SMILES	Provenance evidence / source	SwissADME screening profile	Screening decision / provenance interpretation
65	Ethyl linolenate	-	5367460	1191-41-9	CCC=CCC=CCC=CCCCCCCCC(=O)OCC	Archived record n55 in the merged Fraxini Cortex candidate dataset (chems_add/chems_tiqu); source-level botanical provenance was not recoverable from the archived screening files.	GI High; L/G/V/E/M = 1/1/1/1/1; 0/5 filters with 0 violations.	Excluded at drug-likeness stage: GI = High, but only 0/5 filters showed zero violations (<2/5 required).
66	Ethyl palmitate	-	12366	628-97-7	CCCCCCCCCCCCCCCC(=O)OCC	Archived record n56 in the merged Fraxini Cortex candidate dataset (chems_add/chems_tiqu); source-level botanical provenance was not recoverable from the archived screening files.	GI High; L/G/V/E/M = 1/1/1/1/2; 0/5 filters with 0 violations.	Excluded at drug-likeness stage: GI = High, but only 0/5 filters showed zero violations (<2/5 required).
67	Ethyl pentadecanoate	-	38762	41114-00-5	CCCCCCCCCCCCCCC(=O)OCC	Archived record n57 in the merged Fraxini Cortex candidate dataset (chems_add/chems_tiqu); source-level botanical provenance was not recoverable from the archived screening files.	GI High; L/G/V/E/M = 1/1/1/0/1; 1/5 filters with 0 violations.	Excluded at drug-likeness stage: GI = High, but only 1/5 filters showed zero violations (<2/5 required).
68	Ethyl stearate	-	8122	111-61-5	CCCCCCCCCCCCCCCCCC(=O)OCC	Archived record n58 in the merged Fraxini Cortex candidate dataset (chems_add/chems_tiqu); source-level botanical provenance was not recoverable from the archived screening files.	GI Low; L/G/V/E/M = 1/1/1/1/2; 0/5 filters with 0 violations.	Excluded at GI-absorption stage: SwissADME predicted gastrointestinal absorption = Low.
69	F. bungeana dc.F. bungeana dc.	-	-	-	-	Archived raw record n60 in the merged Fraxini Cortex candidate dataset; the entry could not be standardized to a discrete PubChem chemical identity.	Not evaluated in SwissADME (no valid PubChem CID/usable standardized chemical structure).	Excluded before SwissADME: unresolved/non-standardized record; no valid PubChem CID or usable chemical identity was recovered.
70	F. japonica blumeF. japonica blume	-	-	-	-	Archived raw record n61 in the merged Fraxini Cortex candidate dataset; the entry could not be standardized to a discrete PubChem chemical identity.	Not evaluated in SwissADME (no valid PubChem CID/usable standardized chemical structure).	Excluded before SwissADME: unresolved/non-standardized record; no valid PubChem CID or usable chemical identity was recovered.
71	F. ornus l.F. ornus l.	-	-	-	-	Archived raw record n62 in the merged Fraxini Cortex candidate dataset; the entry could not be standardized to a discrete PubChem chemical identity.	Not evaluated in SwissADME (no valid PubChem CID/usable standardized chemical structure).	Excluded before SwissADME: unresolved/non-standardized record; no valid PubChem CID or usable chemical identity was recovered.
72	Anthracene	-	8418	120-12-7	C1=CC=C2C=C3C=CC=CC3=CC2=C1	Archived record n66 in the merged Fraxini Cortex candidate dataset (chems_add/chems_tiqu); source-level botanical provenance was not recoverable from the archived screening files.	GI Low; L/G/V/E/M = 1/0/0/0/2; 3/5 filters with 0 violations.	Excluded at GI-absorption stage: SwissADME predicted gastrointestinal absorption = Low.
73	Heptadecane	-	12398	629-78-7	CCCCCCCCCCCCCCCCC	Archived record n68 in the merged Fraxini Cortex candidate dataset (chems_add/chems_tiqu); source-level botanical provenance was not recoverable from the archived screening files.	GI Low; L/G/V/E/M = 1/1/1/1/2; 0/5 filters with 0 violations.	Excluded at GI-absorption stage: SwissADME predicted gastrointestinal absorption = Low.
74	Hexadecane	-	11006	544-76-3	CCCCCCCCCCCCCCCC	Archived record n69 in the merged Fraxini Cortex candidate dataset (chems_add/chems_tiqu); source-level botanical provenance was not recoverable from the archived screening files.	GI Low; L/G/V/E/M = 1/1/1/1/2; 0/5 filters with 0 violations.	Excluded at GI-absorption stage: SwissADME predicted gastrointestinal absorption = Low.
75	6,10,14-Trimethylpentadecan-2-one	-	10408	502-69-2	CC(C)CCCC(C)CCCC(C)CCCC(=O)C	Archived record n71 in the merged Fraxini Cortex candidate dataset (chems_add/chems_tiqu); source-level botanical provenance was not recoverable from the archived screening files.	GI High; L/G/V/E/M = 1/1/1/1/2; 0/5 filters with 0 violations.	Excluded at drug-likeness stage: GI = High, but only 0/5 filters showed zero violations (<2/5 required).
76	Linolenic Acid	-	5280934	463-40-1	CCC=CCC=CCC=CCCCCCCCC(=O)O	Archived record n72 in the merged Fraxini Cortex candidate dataset (chems_add/chems_tiqu); source-level botanical provenance was not recoverable from the archived screening files.	GI High; L/G/V/E/M = 1/1/1/1/2; 0/5 filters with 0 violations.	Excluded at drug-likeness stage: GI = High, but only 0/5 filters showed zero violations (<2/5 required).
77	Methyl palmitate	-	8181	112-39-0	CCCCCCCCCCCCCCCC(=O)OC	Archived record n73 in the merged Fraxini Cortex candidate dataset (chems_add/chems_tiqu); source-level botanical provenance was not recoverable from the archived screening files.	GI High; L/G/V/E/M = 1/1/1/0/1; 1/5 filters with 0 violations.	Excluded at drug-likeness stage: GI = High, but only 1/5 filters showed zero violations (<2/5 required).
78	Methyl linoleate	-	5284421	112-63-0	CCCCCC=CCC=CCCCCCCCC(=O)OC	Archived record n74 in the merged Fraxini Cortex candidate dataset (chems_add/chems_tiqu); source-level botanical provenance was not recoverable from the archived screening files.	GI High; L/G/V/E/M = 1/1/1/1/1; 0/5 filters with 0 violations.	Excluded at drug-likeness stage: GI = High, but only 0/5 filters showed zero violations (<2/5 required).
79	Methyl linolenate	-	5319706	301-00-8	CCC=CCC=CCC=CCCCCCCCC(=O)OC	Archived record n75 in the merged Fraxini Cortex candidate dataset (chems_add/chems_tiqu); source-level botanical provenance was not recoverable from the archived screening files.	GI High; L/G/V/E/M = 1/1/1/0/1; 1/5 filters with 0 violations.	Excluded at drug-likeness stage: GI = High, but only 1/5 filters showed zero violations (<2/5 required).
80	Methyl Stearate	-	8201	112-61-8	CCCCCCCCCCCCCCCCCC(=O)OC	Archived record n77 in the merged Fraxini Cortex candidate dataset (chems_add/chems_tiqu); source-level botanical provenance was not recoverable from the archived screening files.	GI High; L/G/V/E/M = 1/1/1/1/2; 0/5 filters with 0 violations.	Excluded at drug-likeness stage: GI = High, but only 0/5 filters showed zero violations (<2/5 required).
81	Myricetin	-	5281672	529-44-2	C1=C(C=C(C(=C1O)O)O)C2=C(C(=O)C3=C(C=C(C=C3O2)O)O)O	Archived record n79 in the merged Fraxini Cortex candidate dataset (chems_add/chems_tiqu); source-level botanical provenance was not recoverable from the archived screening files.	GI Low; L/G/V/E/M = 1/0/1/1/2; 1/5 filters with 0 violations.	Excluded at GI-absorption stage: SwissADME predicted gastrointestinal absorption = Low.

No.	Compound	Chemical class	PubChem CID	CAS No.	Canonical SMILES	Provenance evidence / source	SwissADME screening profile	Screening decision / provenance interpretation
82	Naphthalene	-	931	91-20-3	C1=CC=C2C=CC=CC2=C1	Archived record n80 in the merged Fraxini Cortex candidate dataset (chems_add/chems_tiqu); source-level botanical provenance was not recoverable from the archived screening files.	GI Low; L/G/V/E/M = 1/2/0/0/2; 2/5 filters with 0 violations.	Excluded at GI-absorption stage: SwissADME predicted gastrointestinal absorption = Low.
83	Octadecane	-	11635	593-45-3	CCCCCCCCCCCCCCCCCC	Archived record n82 in the merged Fraxini Cortex candidate dataset (chems_add/chems_tiqu); source-level botanical provenance was not recoverable from the archived screening files.	GI Low; L/G/V/E/M = 1/1/1/1/2; 0/5 filters with 0 violations.	Excluded at GI-absorption stage: SwissADME predicted gastrointestinal absorption = Low.
84	Phenanthrene	-	995	85-01-8	C1=CC=C2C(=C1)C=CC3=CC=CC=C32	Archived record n83 in the merged Fraxini Cortex candidate dataset (chems_add/chems_tiqu); source-level botanical provenance was not recoverable from the archived screening files.	GI Low; L/G/V/E/M = 1/0/0/0/2; 3/5 filters with 0 violations.	Excluded at GI-absorption stage: SwissADME predicted gastrointestinal absorption = Low.
85	Phytol	-	5280435	150-86-7	CC(C)CCCC(C)CCCC(C)CCCC(=CCO)C	Archived record n84 in the merged Fraxini Cortex candidate dataset (chems_add/chems_tiqu); source-level botanical provenance was not recoverable from the archived screening files.	GI Low; L/G/V/E/M = 1/1/1/1/2; 0/5 filters with 0 violations.	Excluded at GI-absorption stage: SwissADME predicted gastrointestinal absorption = Low.
86	Pyrene	-	31423	129-00-0	C1=CC2=C3C(=C1)C=CC4=CC=CC(=C43)C=C2	Archived record n85 in the merged Fraxini Cortex candidate dataset (chems_add/chems_tiqu); source-level botanical provenance was not recoverable from the archived screening files.	GI Low; L/G/V/E/M = 1/0/0/0/1; 3/5 filters with 0 violations.	Excluded at GI-absorption stage: SwissADME predicted gastrointestinal absorption = Low.
87	Oleyl alcohol	-	5284499	143-28-2	CCCCCCCCC=CCCCCCCCCO	Archived record n86 in the merged Fraxini Cortex candidate dataset (chems_add/chems_tiqu); source-level botanical provenance was not recoverable from the archived screening files.	GI High; L/G/V/E/M = 1/1/1/1/2; 0/5 filters with 0 violations.	Excluded at drug-likeness stage: GI = High, but only 0/5 filters showed zero violations (<2/5 required).
88	Spinoside A	-	5281325	119626-74-3	CC(=O)OC1COC(C(C1O)OC(=O)C)OC2CC3(C4CC=C5C(C4(CCC3(C2C(C)(C(=O)C=CC(C)(C)O)O)C)C)C=C(C(=O)C5(C)C)O)C	Archived record n87 in the merged Fraxini Cortex candidate dataset (chems_add/chems_tiqu); source-level botanical provenance was not recoverable from the archived screening files.	GI Low; L/G/V/E/M = 2/3/1/1/3; 0/5 filters with 0 violations.	Excluded at GI-absorption stage: SwissADME predicted gastrointestinal absorption = Low.
89	TrichosanthinTrichosanthin	-	-	-	-	Archived raw record n88 in the merged Fraxini Cortex candidate dataset; the entry could not be standardized to a discrete PubChem chemical identity.	Not evaluated in SwissADME (no valid PubChem CID/usable standardized chemical structure).	Excluded before SwissADME: unresolved/non-standardized record; no valid PubChem CID or usable chemical identity was recovered.
90	Docosane	-	12405	629-97-0	CCCCCCCCCCCCCCCCCCCCCC	Archived record n89 in the merged Fraxini Cortex candidate dataset (chems_add/chems_tiqu); source-level botanical provenance was not recoverable from the archived screening files.	GI Low; L/G/V/E/M = 1/1/1/1/3; 0/5 filters with 0 violations.	Excluded at GI-absorption stage: SwissADME predicted gastrointestinal absorption = Low.
91	Nonadecane	-	12401	629-92-5	CCCCCCCCCCCCCCCCCCC	Archived record n91 in the merged Fraxini Cortex candidate dataset (chems_add/chems_tiqu); source-level botanical provenance was not recoverable from the archived screening files.	GI Low; L/G/V/E/M = 1/1/1/1/3; 0/5 filters with 0 violations.	Excluded at GI-absorption stage: SwissADME predicted gastrointestinal absorption = Low.

Notes: Evidence grades A-D indicate decreasing strength of Fraxini-specific provenance evidence. L/G/V/E/M denote the numbers of violations for the Lipinski, Ghose, Veber, Egan, and Muegge filters, respectively. The predefined retention criterion was High predicted GI absorption plus zero violations in at least two of these five filters. SwissADME outputs are human-oriented in silico descriptors and do not demonstrate actual absorption or bioavailability in laying hens. No study-specific Fraxini Cortex batch was subjected to HPLC-MS, UHPLC-MS, or GC-MS; therefore, provenance grades do not indicate quantitative abundance. For the steroidal glycoside entry, the exact archived computational input was PubChem CID 11968950; it should not be substituted with the distinct PubChem CID 12960498. For the 53 newly added excluded/unresolved records, provenance is reported only when directly recoverable from the archived files. TCMSP Qin Pi records are identified explicitly; where source-level botanical provenance was not preserved in the screening archive, it is reported as not recoverable rather than inferred. Rows 39-91 are new additions in this revision and are shown in red.

Table S1B. Compound-level topological characteristics in the Fraxini Cortex–compound–target network.

No.	Compound	Degree	Betweenness centrality	Closeness centrality	Connectivity tier
1	Furfural	7	0.002330	0.370690	Low
2	Steroidal glycoside (archived CID 11968950)	7	0.001054	0.370690	Low
3	Palmitic acid	7	0.002265	0.370690	Low
4	Fraxetin	10	0.003076	0.375546	Intermediate
5	Benzaldehyde	24	0.019607	0.400000	Intermediate
6	N-Phenyl-2-naphthylamine	6	0.000716	0.369099	Low
7	2-Nonenal	26	0.013047	0.403756	Intermediate
8	Isoamyl alcohol	24	0.015216	0.400000	Intermediate
9	Methyl tetradecanoate	23	0.011047	0.398148	Intermediate
10	Scopoletin	38	0.038202	0.427861	High
11	Benzyl alcohol	24	0.014550	0.400000	Intermediate
12	2,3-Butanediol	4	0.000408	0.365957	Low

No.	Compound	Degree	Betweenness centrality	Closeness centrality	Connectivity tier
13	Geranylacetone	10	0.003250	0.375546	Intermediate
14	2,4-Dimethylphenol	37	0.027159	0.425743	High
15	1-Octen-3-ol	32	0.015093	0.415459	High
16	Methyl pentadecanoate	21	0.008925	0.394495	Intermediate
17	Nonanal	25	0.015159	0.401869	Intermediate
18	Sinapyl alcohol	40	0.046399	0.432161	High
19	Nonan-1-ol	34	0.019617	0.419512	High
20	Dibenzofuran	5	0.000585	0.367521	Low
21	2-Pentadecanone	11	0.001147	0.377193	Intermediate
22	2,6-Dimethoxy-1,4-benzoquinone	31	0.022179	0.413462	High
23	Diacetone alcohol	21	0.007472	0.394495	Intermediate
24	Caffeic acid	42	0.039389	0.436548	High
25	(S)-Acetoin	30	0.020715	0.411483	High
26	Plaunotol	13	0.002284	0.380531	Intermediate
27	Sinapaldehyde	31	0.023244	0.413462	High
28	2-(4-Hydroxyphenyl)ethanol (tyrosol)	31	0.018066	0.413462	High
29	L-Citrulline (citrulline)	22	0.016517	0.396313	Intermediate
30	8-Hydroxypinoresinol	5	0.011749	0.367521	Low
31	1-Hexanol	29	0.012632	0.409524	Intermediate
32	Gamma-aminobutyric acid	21	0.005632	0.394495	Intermediate
33	2-Acetylfuran	3	0.000135	0.364407	Low
34	Esculetin	14	0.003031	0.382222	Intermediate
35	Cedrenol	19	0.004913	0.390909	Intermediate
36	Syringaldehyde	18	0.006372	0.389140	Intermediate
37	4-Methyldibenzofuran	3	0.000252	0.364407	Low
38	Dibutyl phthalate	2	0.000009	0.362869	Low

Network-topology summary: Degree median = 21; Q1 = 7.75 and Q3 = 29.75 (interquartile range width = 22); range = 2–42. Compounds with Degree > Q3 (integer Degree ≥ 30) were classified as high-connectivity (n = 10), those with Degree < Q1 (≤ 7) as low-connectivity (n = 10), and the remainder as intermediate-connectivity (n = 18). Betweenness and Closeness values were taken from the Cytoscape NetworkAnalyzer node-topology output. These categories describe predicted network position only and do not indicate compound abundance, binding affinity, pharmacological activity, or biological efficacy. Archived docking-panel audit: the ten ligands in the archived docking workflow were not identical to the Degree > Q3 high-connectivity tier. The archived panel included 1-Hexanol (Degree = 29; intermediate tier) and 2-Nonenal (Degree = 26; intermediate tier), whereas 2,4-Dimethylphenol (Degree = 37; high tier) and 2,6-Dimethoxy-1,4-benzoquinone (Degree = 31; high tier) were not part of that archived docking panel. Because the original compound-selection rule was not preserved, the revised manuscript does not retrospectively claim that the archived ten ligands were selected by a Degree threshold. The connectivity tiers remain descriptive network categories only.

Table S1C. Compound-level SwissTargetPrediction audit summary for the 38 retained Fraxini Cortex candidate compounds.

The archived SwissTargetPrediction workflow contained 3,800 raw prediction rows (100 predictions for each of 38 retained compounds). Applying the predefined probability threshold (> 0.1) retained 2,464 rows before target-name deduplication. Among these, 141 rows represented complex or multi-protein targets for which the SwissTargetPrediction Common name field was reported as N/A and therefore could not be assigned a single standardized gene symbol. The remaining 2,323 retained rows contained valid human gene symbols; global deduplication of these standardized symbols yielded the reported 528 nonredundant predicted human targets. The complete row-level audit, including target protein name, Common name, UniProt ID, ChEMBL ID, target class, probability score, retention status, standardized gene symbol, and archived source file, is provided in Supplementary Data S1C (Supplementary_Data_S1C_SwissTargetPrediction_Full_Audit.xlsx).

No.	Compound	PubChem CID	Raw predictions	P > 0.1 rows	Mapped gene-symbol rows	Unique gene symbols	Retained probability range
1	Scopoletin	5280460	100	94	93	93	0.104–1.000
2	Caffeic acid	689043	100	100	97	97	0.217–0.859
3	N-Phenyl-2-naphthylamine	8679	100	13	13	13	0.103–0.909
4	Sinapaldehyde	5280802	100	100	97	97	0.111–1.000
5	Sinapyl alcohol	5280507	100	100	96	96	0.160–0.873
6	2-(4-Hydroxyphenyl)ethanol (tyrosol)	10393	100	100	96	96	0.206–1.000
7	Syringaldehyde	8655	100	63	63	63	0.102–0.890
8	Esculetin	5281416	100	62	61	61	0.108–1.000
9	Fraxetin	5273569	100	24	23	23	0.104–0.938
10	2,6-Dimethoxy-1,4-benzoquinone	68262	100	100	97	97	0.104–1.000
11	8-Hydroxypinoresinol	3010930	100	6	6	6	0.136–0.364
12	1-Hexanol	8103	100	100	90	90	0.135–0.754
13	Nonan-1-ol	8914	100	100	94	94	0.183–0.737
14	1-Octen-3-ol	18827	100	100	89	89	0.154–0.825
15	2,3-Butanediol	262	100	28	28	28	0.102–0.204

No.	Compound	PubChem CID	Raw predictions	P > 0.1 rows	Mapped gene-symbol rows	Unique gene symbols	Retained probability range
16	2,4-Dimethylphenol	7771	100	100	93	93	0.122–0.631
17	2-Nonenal	5283335	100	100	95	95	0.107–0.737
18	2-Pentadecanone	61303	100	33	30	30	0.102–0.945
19	Isoamyl alcohol	31260	100	100	96	96	0.116–0.376
20	4-Methyldibenzofuran	43489	100	8	8	8	0.116–0.521
21	2-Acetylfuran	14505	100	3	3	3	0.108–0.728
22	Steroidal glycoside (archived CID 11968950)	11968950	100	35	31	31	0.103–0.215
23	Benzaldehyde	240	100	88	86	86	0.100–0.484
24	Cedrenol	119831	100	48	45	45	0.106–0.986
25	L-Citrulline (citrulline)	9750	100	79	69	69	0.101–0.624
26	Dibenzofuran	568	100	20	20	20	0.131–0.466
27	Dibutyl phthalate	3026	100	10	10	10	0.111–0.146
28	Plaunotol	5282197	100	29	29	29	0.102–0.986
29	Furfural	7362	100	23	21	21	0.101–0.594
30	Gamma-aminobutyric acid	119	100	48	47	47	0.101–0.584
31	Geranylacetone	1549778	100	62	62	62	0.101–0.433
32	(S)-Acetoin	447765	100	88	79	79	0.102–1.000
33	Palmitic acid	985	100	62	53	53	0.101–1.000
34	Methyl pentadecanoate	23518	100	68	61	61	0.103–0.977
35	Methyl tetradecanoate	31284	100	70	63	63	0.103–0.970
36	Nonanal	31289	100	100	87	87	0.130–0.836
37	Diacetone alcohol	31256	100	100	95	95	0.128–0.484
38	Benzyl alcohol	244	100	100	97	97	0.133–0.614

Note. Full row-level prediction data are supplied in the accompanying workbook Supplementary_Data_S1C_SwissTargetPrediction_Full_Audit.xlsx. The workbook contains a compound-level summary, all 3,800 archived SwissTargetPrediction rows before deduplication, and the final 528 nonredundant standardized human gene symbols. Each raw row retains the compound identity, target protein, Common name, UniProt ID, ChEMBL ID, target class, probability score, threshold-retention status, standardized gene symbol where available, and archived source-file path, allowing direct reconstruction of the 38-compound to 528-target workflow.

Table S1D. Provenance sensitivity analysis after removal of lower-confidence/unconfirmed constituents

For a conservative sensitivity analysis, grades A–B were treated as the sufficiently traceable subset and grades C–D were removed. This sensitivity analysis does not replace the primary 38-compound workflow; it tests whether uncertain botanical provenance materially drives downstream prioritization. Detailed row-level outputs are provided in Supplementary_Data_S1D_Provenance_Sensitivity.xlsx, including compound provenance/scenario membership, human-target and Gallus gallus target membership, conservative A–B compound ranking, core-target and docking-receptor retention, docking-ligand provenance, GO/KEGG contributor-retention robustness, and reconstruction checks.

Metric	Full 38 (A–D)	Exclude D only (A–C)	Conservative A–B
Compounds	38	34	15
Nonredundant predicted human targets	528	506	352
Shared human targets	170	166	130
Mapped Gallus gallus targets	133	129	101
STRING v12.0 non-isolated nodes	117	113	89
STRING v12.0 induced edges	685	675	490
Original 13 core targets retained in input	13/13	13/13	13/13
Original 13 core targets in recalculated four-metric Top20 consensus	12/13*	13/13	13/13
Original 6 docking receptors retained in input	6/6	6/6	6/6
Original 6 docking receptors in recalculated four-metric Top20 consensus	6/6	6/6	6/6

*The fully traceable 685-edge reconstruction is used only as the common sensitivity-network reference. The manuscript primary core-target analysis remains the archived 683-edge network; this distinction is preserved to avoid mixing downstream topology outputs from slightly different reconstructions.

Conservative A–B sensitivity results. The A–B subset contained 15 compounds (A=10; B=5). Caffeic acid (Degree=42), sinapyl alcohol (40), and scopoletin (38) remained the three highest-connectivity compounds within this subset. All 13 primary core targets and all six docking receptors remained represented, and all 13 core targets were present in the recalculated Degree/MCC/Betweenness/Closeness Top20 intersection of the A–B induced PPI network (89 nodes, 490 edges).

Pathway robustness. Among the originally BH-significant terms, 99/109 GO terms retained ≥50% of contributing genes and 78/109 retained ≥75% (median 88.9%); 37/39 KEGG pathways retained ≥50% and 29/39 retained ≥75% (median 80.0%). In a deliberately conservative fixed-background hypergeometric stress test with Bonferroni correction over the reconstructed original hypothesis count, 16/20 of the originally top-ranked KEGG pathways remained significant. The retained set included ErbB, focal adhesion, VEGF, C-type lectin receptor, MAPK, efferocytosis, FoxO, Toll-like receptor, NOD-like receptor, apoptosis, and arachidonic acid metabolism. The stress test is a robustness check of originally enriched terms and is not used to replace the primary clusterProfiler analysis.

Docking-prioritization robustness. Eight of the ten archived docking ligands were grades A–B. The two grade-C ligands removed by the conservative filter were (S)-acetoin and 2-nonenal. Scopoletin, caffeic acid, and sinapaldehyde—the compounds represented in the lowest archived docking-score combinations—were all grade A, and both compounds advanced to MD (caffeic acid and scopoletin) were grade A. Thus, lower-confidence/unconfirmed C–D records broadened the predicted target space but did not determine the principal compound, core-target, receptor, or docking priorities.

Accompanying row-level data files. Supplementary_Data_S1C_SwissTargetPrediction_Full_Audit.xlsx provides the complete 3,800-row SwissTargetPrediction audit and the deduplicated 528-target inventory. Supplementary_Data_S1D_Provenance_Sensitivity.xlsx provides the detailed provenance-sensitivity audit supporting Table S1D. Both workbooks were reconstructed from the archived result package and cross-checked against the numerical summaries reported in this Supplementary Data S1.
