# Supplementary material for: Network Pharmacology, Molecular Docking, and Molecular Dynamics Simulation to Explore Predicted Molecular Associations Between Fraxini Cortex Constituents and Intestinal Inflammation in Laying Hens
Source: Vet Sci. 2026 Aug 27;13(9):874. doi: 10.3390/vetsci13090874 (PMC13611608; doi:10.3390/vetsci13090874)
Supplement: Supplementary file 1 [file vetsci-13-00874-s001.zip › Supplementary/Supplementary S2.pdf]

Supplementary Table S2. Human-to-Gallus gallus orthologue mapping, chicken-specific PPI/core-target audit, GO/KEGG enrichment audit, and standardized molecular-docking reproducibility audit

The intersection of 528 compound-related targets and 1,452 disease-related targets produced 170 shared human targets. The human-to-chicken step was rerun against Ensembl release 116 using complete row-level orthology records. Among the 170 human targets, 138 had at least one Gallus gallus orthology record and 32 were unmapped. Of the 138 mapped targets, 133 had at least one Ensembl high-confidence chicken orthologue. To avoid disproportionate weighting of human targets with one-to-many or many-to-many mappings, one representative high-confidence Gallus gallus orthologue was retained for each of these 133 human targets, yielding a final nonredundant 133-gene chicken input set.

Table S2A provides the complete release-pinned audit trail. Each mapped human-chicken relationship is reported with the human Ensembl gene ID, Gallus gallus Ensembl gene ID, Ensembl homology type, high-confidence flag, and the final representative-selection decision; targets with no recovered chicken orthologue are listed explicitly as unmapped. The final 133-gene chicken set is directly derivable from rows marked "Retained" or "Retained: representative". Among the 133 selected representatives, 120 were ortholog_one2one, 6 ortholog_one2many, and 7 ortholog_many2many.

Table S2A. Complete Ensembl release 116 human-to-Gallus gallus orthology audit for the 170 shared human targets

Input no.	Human target	Human Ensembl gene ID	Gallus target	Gallus Ensembl gene ID	Ensembl homology type	High confidence	Final-set decision
1	DPP4	ENSG00000197635	DPP4	ENSGALG00010021045	ortholog_one2many	Yes	Retained
1	DPP4	ENSG00000197635	APEHL	ENSGALG00010027295	ortholog_one2many	No	Excluded: low confidence
2	MMP9	ENSG00000100985	MMP9	ENSGALG00010015061	ortholog_one2one	Yes	Retained
3	ALOX5	ENSG00000012779	ALOX5	ENSGALG00010023818	ortholog_one2one	Yes	Retained
4	MMP2	ENSG00000087245	MMP2	ENSGALG00010025414	ortholog_one2one	Yes	Retained
5	MMP1	ENSG00000196611	MMP1	ENSGALG00010007571	ortholog_one2one	Yes	Retained
6	CA2	ENSG00000104267	CA2	ENSGALG00010013325	ortholog_one2one	Yes	Retained
7	ESR2	ENSG00000140009	ESR2	ENSGALG00010017591	ortholog_one2one	Yes	Retained
8	TYR	ENSG00000077498	TYR	ENSGALG00010007864	ortholog_one2one	Yes	Retained
9	SNCA	ENSG00000145335	SNCA	ENSGALG00010008368	ortholog_one2one	Yes	Retained
10	ESR1	ENSG00000091831	ESR1	ENSGALG00010005186	ortholog_one2one	Yes	Retained
11	APP	ENSG00000142192	APP	ENSGALG00010010915	ortholog_one2one	Yes	Retained
12	LCK	ENSG00000182866	LCK	ENSGALG00010017442	ortholog_one2one	Yes	Retained
13	TTR	ENSG00000118271	TTRL	ENSGALG00010004315	ortholog_one2many	Yes	Excluded: alternate HC
13	TTR	ENSG00000118271	TTR	ENSGALG00010006069	ortholog_one2many	Yes	Retained: representative
14	PIK3CB	ENSG00000051382	PIK3CB	ENSGALG00010025125	ortholog_one2one	Yes	Retained
15	PIK3CA	ENSG00000121879	PIK3CA	ENSGALG00010018296	ortholog_one2one	Yes	Retained
16	SYK	ENSG00000165025	SYK	ENSGALG00010009340	ortholog_one2one	Yes	Retained
17	PTGS2	ENSG00000073756	PTGS2	ENSGALG00010021038	ortholog_one2one	Yes	Retained
18	PTGS1	ENSG00000095303	PTGS1	ENSGALG00010028372	ortholog_one2one	Yes	Retained
19	EGFR	ENSG00000146648	EGFR	ENSGALG00010007381	ortholog_one2one	Yes	Retained
20	AKR1B10	ENSG00000198074	Not assigned in Ensembl 116	ENSGALG00010013395	ortholog_many2many	No	Excluded: low confidence
20	AKR1B10	ENSG00000198074	Not assigned in Ensembl 116	ENSGALG00010013401	ortholog_many2many	Yes	Excluded: alternate HC
20	AKR1B10	ENSG00000198074	AKR1B10	ENSGALG00010013501	ortholog_many2many	Yes	Retained: representative
21	ELANE	ENSG00000197561; ENSG00000277571	—	—	Unmapped	No	Excluded: no chicken orthologue
22	MIF	ENSG00000240972	MIF	ENSGALG00010026095	ortholog_one2one	Yes	Retained
23	ALOX15	ENSG00000161905	ALOX15B	ENSGALG00010022261	ortholog_one2one	No	Excluded: low confidence
24	BCHE	ENSG00000114200	BCHE	ENSGALG00010001267	ortholog_one2one	Yes	Retained
25	ERBB2	ENSG00000141736	ERBB2	ENSGALG00010024479	ortholog_one2one	Yes	Retained
26	CYP1B1	ENSG00000138061	CYP1B1	ENSGALG00010007388	ortholog_one2one	Yes	Retained
27	XIAP	ENSG00000101966	BIRC8	ENSGALG00010013281	ortholog_one2one	Yes	Retained
28	TRPA1	ENSG00000104321	TRPA1	ENSGALG00010002600	ortholog_one2one	Yes	Retained
29	MET	ENSG00000105976	MET	ENSGALG00010001514	ortholog_one2one	Yes	Retained
30	NFE2L2	ENSG00000116044	NFE2L2	ENSGALG00010024107	ortholog_one2one	Yes	Retained
31	CYP2C19	ENSG00000165841	—	—	Unmapped	No	Excluded: no chicken orthologue
32	CYP2C9	ENSG00000138109	—	—	Unmapped	No	Excluded: no chicken orthologue
33	CYP3A4	ENSG00000160868	CYP3A5	ENSGALG00010017603	ortholog_one2many	Yes	Retained
34	CYP1A2	ENSG00000140505	CYP1A2	ENSGALG00010021117	ortholog_many2many	Yes	Retained
34	CYP1A2	ENSG00000140505	CYP1A1	ENSGALG00010021122	ortholog_many2many	No	Excluded: low confidence
35	MAPK1	ENSG00000100030	MAPK3	ENSGALG00010024358	ortholog_one2one	Yes	Retained

Input no.	Human target	Human Ensembl gene ID	Gallus target	Gallus Ensembl gene ID	Ensembl homology type	High confidence	Final-set decision
36	AKR1C3	ENSG00000196139	Not assigned in Ensembl 116	ENSGALG00010013395	ortholog_many2many	No	Excluded: low confidence
36	AKR1C3	ENSG00000196139	Not assigned in Ensembl 116	ENSGALG00010013401	ortholog_many2many	No	Excluded: low confidence
36	AKR1C3	ENSG00000196139	AKR1B10	ENSGALG00010013501	ortholog_many2many	No	Excluded: low confidence
37	STAT3	ENSG00000168610	STAT3	ENSGALG00010026098	ortholog_one2one	Yes	Retained
38	MAPT	ENSG00000186868	MAPT	ENSGALG00010024833	ortholog_one2one	Yes	Retained
39	CYP1A1	ENSG00000140465	CYP1A2	ENSGALG00010021117	ortholog_many2many	No	Excluded: low confidence
39	CYP1A1	ENSG00000140465	CYP1A1	ENSGALG00010021122	ortholog_many2many	Yes	Retained
40	PRKCQ	ENSG00000065675	PRKCQ	ENSGALG00010005409	ortholog_one2one	Yes	Retained
41	IDO1	ENSG00000131203	—	—	Unmapped	No	Excluded: no chicken orthologue
42	HSD11B1	ENSG00000117594	HSD11B1A	ENSGALG00010027203	ortholog_one2many	Yes	Retained: representative
42	HSD11B1	ENSG00000117594	HSD11B1B	ENSGALG00010027206	ortholog_one2many	Yes	Excluded: alternate HC
43	ABCB1	ENSG00000085563	ABCB1LA	ENSGALG00010005222	ortholog_one2one	Yes	Retained
44	ABCG2	ENSG00000118777	—	—	Unmapped	No	Excluded: no chicken orthologue
45	AHR	ENSG00000106546	AHR1A	ENSGALG00010004433	ortholog_one2one	Yes	Retained
46	RELA	ENSG00000173039	Not assigned in Ensembl 116	ENSGALG00010001277	ortholog_one2one	No	Excluded: low confidence
47	GSK3B	ENSG00000082701	GSK3A	ENSGALG00010025103	ortholog_one2one	Yes	Retained
48	EP300	ENSG00000100393	EP300	ENSGALG00010012865	ortholog_one2one	Yes	Retained
49	TLR9	ENSG00000239732	—	—	Unmapped	No	Excluded: no chicken orthologue
50	NFKB1	ENSG00000109320	NFKB1	ENSGALG00010005476	ortholog_one2one	Yes	Retained
51	JUN	ENSG00000177606	JUN	ENSGALG00010026886	ortholog_one2one	Yes	Retained
52	ABL1	ENSG00000097007	ABL1	ENSGALG00010027607	ortholog_one2one	Yes	Retained
53	CXCL12	ENSG00000107562	CXCL12	ENSGALG00010021867	ortholog_one2one	Yes	Retained
54	HDAC2	ENSG00000196591	HDAC2	ENSGALG00010013392	ortholog_one2one	Yes	Retained
55	F3	ENSG00000117525	F3	ENSGALG00010021018	ortholog_one2one	Yes	Retained
56	NOS2	ENSG00000007171	NOS2	ENSGALG00010029552	ortholog_one2one	Yes	Retained
57	ACHE	ENSG00000087085	ACHE	ENSGALG00010000453	ortholog_one2one	Yes	Retained
58	MAPK14	ENSG00000112062	MAPK14	ENSGALG00010027041	ortholog_one2one	Yes	Retained
59	XDH	ENSG00000158125	XDH	ENSGALG00010018628	ortholog_one2one	Yes	Retained
60	MGAM	ENSG00000257335	SI	ENSGALG00010001273	ortholog_many2many	Yes	Retained
60	MGAM	ENSG00000257335	MGAM	ENSGALG00010002126	ortholog_many2many	No	Excluded: low confidence
60	MGAM	ENSG00000257335	Not assigned in Ensembl 116	ENSGALG00010022789	ortholog_many2many	No	Excluded: low confidence
61	G6PD	ENSG00000160211	—	—	Unmapped	No	Excluded: no chicken orthologue
62	GPR35	ENSG00000178623	Not assigned in Ensembl 116	ENSGALG00010025648	ortholog_one2many	No	Excluded: low confidence
62	GPR35	ENSG00000178623	Not assigned in Ensembl 116	ENSGALG00010025657	ortholog_one2many	No	Excluded: low confidence
63	PDGFRB	ENSG00000113721	PDGFRB	ENSGALG00010016785	ortholog_one2one	Yes	Retained
64	KDR	ENSG00000128052	KDR	ENSGALG00010013498	ortholog_one2one	Yes	Retained
65	SRC	ENSG00000197122	SRC	ENSGALG00010021638	ortholog_one2one	Yes	Retained
66	IGF1R	ENSG00000140443	IGF1R	ENSGALG00010018730	ortholog_one2one	Yes	Retained
67	AKT1	ENSG00000142208	AKT1	ENSGALG00010017364	ortholog_one2one	Yes	Retained
68	IKBKB	ENSG00000104365	IKBKB	ENSGALG00010017955	ortholog_one2one	Yes	Retained
69	SIRT1	ENSG00000096717	SIRT1	ENSGALG00010009252	ortholog_one2one	Yes	Retained
70	AGER	ENSG00000204305; ENSG00000206320; ENSG00000229058; ENSG00000230514; ENSG00000231268; ENSG00000234729; ENSG00000237405	—	—	Unmapped	No	Excluded: no chicken orthologue
71	BRAF	ENSG00000157764	BRAF	ENSGALG00010013466	ortholog_one2one	Yes	Retained
72	FLT4	ENSG00000037280	FLT4	ENSGALG00010016867	ortholog_one2one	Yes	Retained
73	TEK	ENSG00000120156	TEK	ENSGALG00010006538	ortholog_one2one	Yes	Retained
74	KCNA1	ENSG00000111262	KCNA1	ENSGALG00010024400	ortholog_one2one	Yes	Retained
75	NTRK1	ENSG00000198400	NTRK1	ENSGALG00010028736	ortholog_one2one	Yes	Retained
76	MALT1	ENSG00000172175	PCASP1	ENSGALG00010010352	ortholog_one2one	Yes	Retained
77	ODC1	ENSG00000115758	ODC	ENSGALG00010003604	ortholog_one2one	Yes	Retained
78	PPARA	ENSG00000186951	PPARA	ENSGALG00010023058	ortholog_one2one	Yes	Retained

Input no.	Human target	Human Ensembl gene ID	Gallus target	Gallus Ensembl gene ID	Ensembl homology type	High confidence	Final-set decision
79	TRPV1	ENSG00000196689	TRPV1	ENSGALG00010029746	ortholog_one2one	Yes	Retained
80	CD274	ENSG00000120217	—	—	Unmapped	No	Excluded: no chicken orthologue
81	ALOX12	ENSG00000108839	—	—	Unmapped	No	Excluded: no chicken orthologue
82	PTPRC	ENSG00000081237	PTPRC	ENSGALG00010012352	ortholog_one2one	Yes	Retained
83	KDM6B	ENSG00000132510	—	—	Unmapped	No	Excluded: no chicken orthologue
84	SLC6A4	ENSG00000108576	SLC6A4	ENSGALG00010029709	ortholog_one2one	Yes	Retained
85	ALB	ENSG00000163631	ALB	ENSGALG00010005070	ortholog_one2one	Yes	Retained
86	BCL2	ENSG00000171791	BCL2	ENSGALG00010011843	ortholog_one2one	Yes	Retained
87	LTB4R	ENSG00000213903	LTB4R	ENSGALG00010003212	ortholog_one2one	Yes	Retained
88	CES2	ENSG00000172831	—	—	Unmapped	No	Excluded: no chicken orthologue
89	NR1H4	ENSG00000012504	NR1H4	ENSGALG00010011566	ortholog_one2one	Yes	Retained
90	BCL2L1	ENSG00000171552	BCL2L1	ENSGALG00010014066	ortholog_one2one	Yes	Retained
91	PTPN11	ENSG00000179295	PTPN11	ENSGALG00010020701	ortholog_one2one	Yes	Retained
92	F2	ENSG00000180210	—	—	Unmapped	No	Excluded: no chicken orthologue
93	SERPINE1	ENSG00000106366	—	—	Unmapped	No	Excluded: no chicken orthologue
94	HIF1A	ENSG00000100644	HIF1A	ENSGALG00010017810	ortholog_one2one	Yes	Retained
95	SOAT2	ENSG00000167780	—	—	Unmapped	No	Excluded: no chicken orthologue
96	SLC5A1	ENSG00000100170	SLC5A1	ENSGALG00010026633	ortholog_one2one	Yes	Retained
97	ALK	ENSG00000171094	ALK	ENSGALG00010017563	ortholog_one2one	Yes	Retained
98	AXL	ENSG00000167601	—	—	Unmapped	No	Excluded: no chicken orthologue
99	PTK2	ENSG00000169398	PTK2	ENSGALG00010009159	ortholog_one2one	Yes	Retained
100	NT5E	ENSG00000135318	NT5E	ENSGALG00010004224	ortholog_one2one	Yes	Retained
101	PLA2G2A	ENSG00000188257	PLA2G5	ENSGALG00010020717	ortholog_one2one	Yes	Retained
102	NR1I3	ENSG00000143257	NR1I3	ENSGALG00010027496	ortholog_one2one	Yes	Retained
103	CYP8B1	ENSG00000180432	ACKR2	ENSGALG00010027403	ortholog_one2one	Yes	Retained
104	GPR84	ENSG00000139572	—	—	Unmapped	No	Excluded: no chicken orthologue
105	SLC10A1	ENSG00000100652	SLC10A1	ENSGALG00010021491	ortholog_one2one	Yes	Retained
106	NR3C2	ENSG00000151623	NR3C2	ENSGALG00010006735	ortholog_one2one	Yes	Retained
107	NR3C1	ENSG00000113580	NR3C1	ENSGALG00010016758	ortholog_one2one	Yes	Retained
108	PPARD	ENSG00000112033	PPARD	ENSGALG00010027545	ortholog_one2one	Yes	Retained
109	PPARG	ENSG00000132170	PPARG	ENSGALG00010027917	ortholog_one2one	Yes	Retained
110	NR1H3	ENSG00000025434	NR1H3	ENSGALG00010022650	ortholog_one2one	Yes	Retained
111	HSD11B2	ENSG00000176387	HSD11B2	ENSGALG00010021624	ortholog_one2one	Yes	Retained
112	NPC1L1	ENSG00000015520	—	—	Unmapped	No	Excluded: no chicken orthologue
113	TLR4	ENSG00000136869	TLR4	ENSGALG00010028914	ortholog_one2one	Yes	Retained
114	GPBAR1	ENSG00000179921	—	—	Unmapped	No	Excluded: no chicken orthologue
115	F2RL1	ENSG00000164251	—	—	Unmapped	No	Excluded: no chicken orthologue
116	STAT1	ENSG00000115415	STAT1	ENSGALG00010013934	ortholog_one2one	Yes	Retained
117	RORC	ENSG00000143365	Not assigned in Ensembl 116	ENSGALG00010028006	ortholog_one2many	Yes	Excluded: alternate HC
117	RORC	ENSG00000143365	RORC	ENSGALG00010028041	ortholog_one2many	Yes	Retained: representative
118	TERT	ENSG00000164362	TERT	ENSGALG00010008150	ortholog_one2one	Yes	Retained
119	NR1H2	ENSG00000131408	—	—	Unmapped	No	Excluded: no chicken orthologue
120	MAPK3	ENSG00000102882	—	—	Unmapped	No	Excluded: no chicken orthologue
121	PTGER4	ENSG00000171522	PTGER4	ENSGALG00010014350	ortholog_one2one	Yes	Retained
122	PRKCA	ENSG00000154229	PRKCA	ENSGALG00010029209	ortholog_one2one	Yes	Retained
123	VDR	ENSG00000111424	VDR	ENSGALG00010024416	ortholog_one2one	Yes	Retained
124	TBXAS1	ENSG00000059377	TBXAS1	ENSGALG00010013152	ortholog_one2one	Yes	Retained
125	PTPN2	ENSG00000175354	PTPN2	ENSGALG00010001185	ortholog_one2one	Yes	Retained
126	SELE	ENSG00000007908	—	—	Unmapped	No	Excluded: no chicken orthologue
127	ICAM1	ENSG00000090339	—	—	Unmapped	No	Excluded: no chicken orthologue
128	LTA4H	ENSG00000111144	LTA4H	ENSGALG00010011903	ortholog_one2one	Yes	Retained
129	GBA1	ENSG00000177628	Not assigned in Ensembl 116	ENSGALG00010027949	ortholog_one2many	Yes	Retained: representative

Input no.	Human target	Human Ensembl gene ID	Gallus target	Gallus Ensembl gene ID	Ensembl homology type	High confidence	Final-set decision
129	GBA1	ENSG00000177628	Not assigned in Ensembl 116	ENSGALG00010027955	ortholog_one2many	Yes	Excluded: alternate HC
130	HRH4	ENSG00000134489	—	—	Unmapped	No	Excluded: no chicken orthologue
131	NR1I2	ENSG00000144852	—	—	Unmapped	No	Excluded: no chicken orthologue
132	CCR5	ENSG00000160791	CCR5	ENSGALG00010017278	ortholog_many2many	Yes	Retained: representative
132	CCR5	ENSG00000160791	CCR2	ENSGALG00010017351	ortholog_many2many	Yes	Excluded: alternate HC
133	F13A1	ENSG00000124491	F13A1	ENSGALG00010010923	ortholog_one2one	Yes	Retained
134	PLA2G4A	ENSG00000116711	PLA2G4A	ENSGALG00010021027	ortholog_one2one	Yes	Retained
135	TNF	ENSG00000204490; ENSG00000206439; ENSG00000223952; ENSG00000228321; ENSG00000228849; ENSG00000228978; ENSG00000230108; ENSG00000232810	—	—	Unmapped	No	Excluded: no chicken orthologue
136	JAK2	ENSG00000096968	JAK2	ENSGALG00010012833	ortholog_one2one	Yes	Retained
137	GSR	ENSG00000104687	GSR	ENSGALG00010008016	ortholog_one2one	Yes	Retained
138	TLR8	ENSG00000101916	—	—	Unmapped	No	Excluded: no chicken orthologue
139	COMT	ENSG00000093010	COMT	ENSGALG00010024592	ortholog_one2one	Yes	Retained
140	LRRK2	ENSG00000188906	LRRK2	ENSGALG00010009361	ortholog_one2one	Yes	Retained
141	JAK3	ENSG00000105639	JAK3	ENSGALG00010027619	ortholog_one2one	Yes	Retained
142	JAK1	ENSG00000162434	JAK1	ENSGALG00010024493	ortholog_one2one	Yes	Retained
143	CREBBP	ENSG00000005339	CREBBP	ENSGALG00010016508	ortholog_one2one	Yes	Retained
144	PCSK9	ENSG00000169174	PCSK9	ENSGALG00010022791	ortholog_one2one	Yes	Retained
145	CSF1R	ENSG00000182578	CSF1R	ENSGALG00010016804	ortholog_one2one	Yes	Retained
146	TGFBR1	ENSG00000106799	TGFBR1	ENSGALG00010007951	ortholog_one2one	Yes	Retained
147	PLAUR	ENSG00000011422	—	—	Unmapped	No	Excluded: no chicken orthologue
148	CTNNB1	ENSG00000168036	CTNNB1	ENSGALG00010016258	ortholog_one2one	Yes	Retained
149	NOS1	ENSG00000089250	NOS1	ENSGALG00010000576	ortholog_one2one	Yes	Retained
150	MAP2K1	ENSG00000169032	MAP2K1	ENSGALG00010018000	ortholog_one2one	Yes	Retained
151	HMOX1	ENSG00000100292	HMOX1	ENSGALG00010015155	ortholog_one2one	Yes	Retained
152	PLAU	ENSG00000122861	—	—	Unmapped	No	Excluded: no chicken orthologue
153	MT-CO2	ENSG00000198712	COX2	ENSGALG00010000020	ortholog_one2one	Yes	Retained
154	NOS3	ENSG00000164867	NOS3	ENSGALG00010027175	ortholog_one2one	Yes	Retained
155	ACE	ENSG00000159640	—	—	Unmapped	No	Excluded: no chicken orthologue
156	FOLH1	ENSG00000086205	FOLH1	ENSGALG00010007404	ortholog_one2one	Yes	Retained
157	SLC1A1	ENSG00000106688	SLC1A1	ENSGALG00010011937	ortholog_one2one	Yes	Retained
158	ECE1	ENSG00000117298	ECE1	ENSGALG00010021126	ortholog_one2one	Yes	Retained
159	MEP1B	ENSG00000141434	MEP1B	ENSGALG00010004061	ortholog_one2one	Yes	Retained
160	PRSS1	ENSG00000204983	Not assigned in Ensembl 116	ENSGALG00010023495	ortholog_many2many	Yes	Excluded: alternate HC
160	PRSS1	ENSG00000204983	Not assigned in Ensembl 116	ENSGALG00010023501	ortholog_many2many	Yes	Excluded: alternate HC
160	PRSS1	ENSG00000204983	PRSS2	ENSGALG00010023541	ortholog_many2many	Yes	Retained: representative
160	PRSS1	ENSG00000204983	Not assigned in Ensembl 116	ENSGALG00010026260	ortholog_many2many	Yes	Excluded: alternate HC
161	PRKCD	ENSG00000163932	PRKCD	ENSGALG00010028105	ortholog_one2one	Yes	Retained
162	CYP4F2	ENSG00000186115	CYP4B7	ENSGALG00010022917	ortholog_one2many	No	Excluded: low confidence
163	PIK3CG	ENSG00000105851	PIK3CG	ENSGALG00010009050	ortholog_one2one	Yes	Retained
164	MTOR	ENSG00000198793	MTOR	ENSGALG00010021746	ortholog_one2one	Yes	Retained
165	FABP2	ENSG00000145384	FABP2	ENSGALG00010005505	ortholog_one2one	Yes	Retained
166	PTGER2	ENSG00000125384	PTGER2	ENSGALG00010017954	ortholog_one2one	Yes	Retained
167	PLG	ENSG00000122194	Not assigned in Ensembl 116	ENSGALG00010006269	ortholog_many2many	No	Excluded: low confidence
167	PLG	ENSG00000122194	PLG	ENSGALG00010007226	ortholog_many2many	Yes	Retained
168	GLI1	ENSG00000111087	GLI1	ENSGALG00010025727	ortholog_one2one	Yes	Retained
169	VCAM1	ENSG00000162692	—	—	Unmapped	No	Excluded: no chicken orthologue
170	HTR3A	ENSG00000166736	HTR3A	ENSGALG00010024453	ortholog_one2one	Yes	Retained

Table S2B. Final Ensembl release 116 representative high-confidence Gallus gallus orthologue set used for downstream analyses (n = 133)

The final set contains 133 unique Gallus gallus Ensembl gene IDs, representing one high-confidence chicken orthologue for each retained human target. One selected release-116 chicken gene (the representative for human GBA1) does not have an assigned Gallus gallus gene symbol and is therefore retained by its Ensembl gene ID rather than being discarded or assigned an inferred symbol.

No.	Gallus target	Gallus Ensembl gene ID	Source human	Homology type	No.	Gallus target	Gallus Ensembl gene ID	Source human	Homology type
1	ABCB1LA	ENSGALG00010005222	ABCB1	ortholog_one2one	68	MIF	ENSGALG00010026095	MIF	ortholog_one2one
2	ABL1	ENSGALG00010027607	ABL1	ortholog_one2one	69	MMP1	ENSGALG00010007571	MMP1	ortholog_one2one
3	ACHE	ENSGALG00010000453	ACHE	ortholog_one2one	70	MMP2	ENSGALG00010025414	MMP2	ortholog_one2one
4	ACKR2	ENSGALG00010027403	CYP8B1	ortholog_one2one	71	MMP9	ENSGALG00010015061	MMP9	ortholog_one2one
5	AHR1A	ENSGALG00010004433	AHR	ortholog_one2one	72	MTOR	ENSGALG00010021746	MTOR	ortholog_one2one
6	AKR1B10	ENSGALG00010013501	AKR1B10	ortholog_many2many	73	NFE2L2	ENSGALG00010024107	NFE2L2	ortholog_one2one
7	AKT1	ENSGALG00010017364	AKT1	ortholog_one2one	74	NFKB1	ENSGALG00010005476	NFKB1	ortholog_one2one
8	ALB	ENSGALG00010005070	ALB	ortholog_one2one	75	NOS1	ENSGALG00010000576	NOS1	ortholog_one2one
9	ALK	ENSGALG00010017563	ALK	ortholog_one2one	76	NOS2	ENSGALG00010029552	NOS2	ortholog_one2one
10	ALOX5	ENSGALG00010023818	ALOX5	ortholog_one2one	77	NOS3	ENSGALG00010027175	NOS3	ortholog_one2one
11	APP	ENSGALG00010010915	APP	ortholog_one2one	78	NR1H3	ENSGALG00010022650	NR1H3	ortholog_one2one
12	BCHE	ENSGALG00010001267	BCHE	ortholog_one2one	79	NR1H4	ENSGALG00010011566	NR1H4	ortholog_one2one
13	BCL2	ENSGALG00010011843	BCL2	ortholog_one2one	80	NR1I3	ENSGALG00010027496	NR1I3	ortholog_one2one
14	BCL2L1	ENSGALG00010014066	BCL2L1	ortholog_one2one	81	NR3C1	ENSGALG00010016758	NR3C1	ortholog_one2one
15	BIRC8	ENSGALG00010013281	XIAP	ortholog_one2one	82	NR3C2	ENSGALG00010006735	NR3C2	ortholog_one2one
16	BRAF	ENSGALG00010013466	BRAF	ortholog_one2one	83	NT5E	ENSGALG00010004224	NT5E	ortholog_one2one
17	CA2	ENSGALG00010013325	CA2	ortholog_one2one	84	NTRK1	ENSGALG00010028736	NTRK1	ortholog_one2one
18	CCR5	ENSGALG00010017278	CCR5	ortholog_many2many	85	ODC	ENSGALG00010003604	ODC1	ortholog_one2one
19	COMT	ENSGALG00010024592	COMT	ortholog_one2one	86	PCASP1	ENSGALG00010010352	MALT1	ortholog_one2one
20	COX2	ENSGALG00010000020	MT-CO2	ortholog_one2one	87	PCSK9	ENSGALG00010022791	PCSK9	ortholog_one2one
21	CREBBP	ENSGALG00010016508	CREBBP	ortholog_one2one	88	PDGFRB	ENSGALG00010016785	PDGFRB	ortholog_one2one
22	CSF1R	ENSGALG00010016804	CSF1R	ortholog_one2one	89	PIK3CA	ENSGALG00010018296	PIK3CA	ortholog_one2one
23	CTNNB1	ENSGALG00010016258	CTNNB1	ortholog_one2one	90	PIK3CB	ENSGALG00010025125	PIK3CB	ortholog_one2one
24	CXCL12	ENSGALG00010021867	CXCL12	ortholog_one2one	91	PIK3CG	ENSGALG00010009050	PIK3CG	ortholog_one2one
25	CYP1A1	ENSGALG00010021122	CYP1A1	ortholog_many2many	92	PLA2G4A	ENSGALG00010021027	PLA2G4A	ortholog_one2one
26	CYP1A2	ENSGALG00010021117	CYP1A2	ortholog_many2many	93	PLA2G5	ENSGALG00010020717	PLA2G2A	ortholog_one2one
27	CYP1B1	ENSGALG00010007388	CYP1B1	ortholog_one2one	94	PLG	ENSGALG00010007226	PLG	ortholog_many2many
28	CYP3A5	ENSGALG00010017603	CYP3A4	ortholog_one2many	95	PPARA	ENSGALG00010023058	PPARA	ortholog_one2one
29	DPP4	ENSGALG00010021045	DPP4	ortholog_one2many	96	PPARD	ENSGALG00010027545	PPARD	ortholog_one2one
30	ECE1	ENSGALG00010021126	ECE1	ortholog_one2one	97	PPARG	ENSGALG00010027917	PPARG	ortholog_one2one
31	EGFR	ENSGALG00010007381	EGFR	ortholog_one2one	98	PRKCA	ENSGALG00010029209	PRKCA	ortholog_one2one
32	EP300	ENSGALG00010012865	EP300	ortholog_one2one	99	PRKCD	ENSGALG00010028105	PRKCD	ortholog_one2one
33	ERBB2	ENSGALG00010024479	ERBB2	ortholog_one2one	100	PRKCQ	ENSGALG00010005409	PRKCQ	ortholog_one2one
34	ESR1	ENSGALG00010005186	ESR1	ortholog_one2one	101	PRSS2	ENSGALG00010023541	PRSS1	ortholog_many2many
35	ESR2	ENSGALG00010017591	ESR2	ortholog_one2one	102	PTGER2	ENSGALG00010017954	PTGER2	ortholog_one2one
36	F13A1	ENSGALG00010010923	F13A1	ortholog_one2one	103	PTGER4	ENSGALG00010014350	PTGER4	ortholog_one2one
37	F3	ENSGALG00010021018	F3	ortholog_one2one	104	PTGS1	ENSGALG00010028372	PTGS1	ortholog_one2one
38	FABP2	ENSGALG00010005505	FABP2	ortholog_one2one	105	PTGS2	ENSGALG00010021038	PTGS2	ortholog_one2one
39	FLT4	ENSGALG00010016867	FLT4	ortholog_one2one	106	PTK2	ENSGALG00010009159	PTK2	ortholog_one2one
40	FOLH1	ENSGALG00010007404	FOLH1	ortholog_one2one	107	PTPN11	ENSGALG00010020701	PTPN11	ortholog_one2one
41	GLI1	ENSGALG00010025727	GLI1	ortholog_one2one	108	PTPN2	ENSGALG00010001185	PTPN2	ortholog_one2one
42	GSK3A	ENSGALG00010025103	GSK3B	ortholog_one2one	109	PTPRC	ENSGALG00010012352	PTPRC	ortholog_one2one
43	GSR	ENSGALG00010008016	GSR	ortholog_one2one	110	RORC	ENSGALG00010028041	RORC	ortholog_one2many
44	HDAC2	ENSGALG00010013392	HDAC2	ortholog_one2one	111	SI	ENSGALG00010001273	MGAM	ortholog_many2many
45	HIF1A	ENSGALG00010017810	HIF1A	ortholog_one2one	112	SIRT1	ENSGALG00010009252	SIRT1	ortholog_one2one
46	HMOX1	ENSGALG00010015155	HMOX1	ortholog_one2one	113	SLC10A1	ENSGALG00010021491	SLC10A1	ortholog_one2one
47	HSD11B1A	ENSGALG00010027203	HSD11B1	ortholog_one2many	114	SLC1A1	ENSGALG00010011937	SLC1A1	ortholog_one2one
48	HSD11B2	ENSGALG00010021624	HSD11B2	ortholog_one2one	115	SLC5A1	ENSGALG00010026633	SLC5A1	ortholog_one2one
49	HTR3A	ENSGALG00010024453	HTR3A	ortholog_one2one	116	SLC6A4	ENSGALG00010029709	SLC6A4	ortholog_one2one
50	IGF1R	ENSGALG00010018730	IGF1R	ortholog_one2one	117	SNCA	ENSGALG00010008368	SNCA	ortholog_one2one
51	IKBKB	ENSGALG00010017955	IKBKB	ortholog_one2one	118	SRC	ENSGALG00010021638	SRC	ortholog_one2one
52	JAK1	ENSGALG00010024493	JAK1	ortholog_one2one	119	STAT1	ENSGALG00010013934	STAT1	ortholog_one2one

No.	Gallus target	Gallus Ensembl gene ID	Source human	Homology type	No.	Gallus target	Gallus Ensembl gene ID	Source human	Homology type
53	JAK2	ENSGALG00010012833	JAK2	ortholog_one2one	120	STAT3	ENSGALG00010026098	STAT3	ortholog_one2one
54	JAK3	ENSGALG00010027619	JAK3	ortholog_one2one	121	SYK	ENSGALG00010009340	SYK	ortholog_one2one
55	JUN	ENSGALG00010026886	JUN	ortholog_one2one	122	TBXAS1	ENSGALG00010013152	TBXAS1	ortholog_one2one
56	KCNA1	ENSGALG00010024400	KCNA1	ortholog_one2one	123	TEK	ENSGALG00010006538	TEK	ortholog_one2one
57	KDR	ENSGALG00010013498	KDR	ortholog_one2one	124	TERT	ENSGALG00010008150	TERT	ortholog_one2one
58	LCK	ENSGALG00010017442	LCK	ortholog_one2one	125	TGFBR1	ENSGALG00010007951	TGFBR1	ortholog_one2one
59	LRRK2	ENSGALG00010009361	LRRK2	ortholog_one2one	126	TLR4	ENSGALG00010028914	TLR4	ortholog_one2one
60	LTA4H	ENSGALG00010011903	LTA4H	ortholog_one2one	127	TRPA1	ENSGALG00010002600	TRPA1	ortholog_one2one
61	LTB4R	ENSGALG00010003212	LTB4R	ortholog_one2one	128	TRPV1	ENSGALG00010029746	TRPV1	ortholog_one2one
62	MAP2K1	ENSGALG00010018000	MAP2K1	ortholog_one2one	129	TTR	ENSGALG00010006069	TTR	ortholog_one2many
63	MAPK14	ENSGALG00010027041	MAPK14	ortholog_one2one	130	TYR	ENSGALG00010007864	TYR	ortholog_one2one
64	MAPK3	ENSGALG00010024358	MAPK1	ortholog_one2one	131	VDR	ENSGALG00010024416	VDR	ortholog_one2one
65	MAPT	ENSGALG00010024833	MAPT	ortholog_one2one	132	XDH	ENSGALG00010018628	XDH	ortholog_one2one
66	MEP1B	ENSGALG00010004061	MEP1B	ortholog_one2one	133	Not assigned in Ensembl 116	ENSGALG00010027949	GBA1	ortholog_one2many
67	MET	ENSGALG00010001514	MET	ortholog_one2one					

Ensembl release and representative-selection rule. Orthology relationships were rerun against Ensembl release 116 and all pairwise records are preserved in Table S2A. For human targets with more than one high-confidence chicken orthologue, a single representative was selected using a deterministic hierarchy: (1) an exact human/chicken gene-symbol match was preferred when available; (2) an assigned Gallus gallus gene symbol was preferred over an unassigned Ensembl gene; and (3) any remaining tie was resolved by ascending Gallus gallus Ensembl gene ID. This representative selection prevents one human target from contributing multiple chicken inputs and does not imply that excluded paralogous orthologues are biologically inferior.

STRING v12.0 identifier traceability and 133→117 audit. The 133 representative Gallus gallus targets in Table S2B were re-audited against the supplied Gallus gallus STRING v12.0 protein.aliases, protein.info, and protein.links files (NCBI taxon 9031). Exact chicken input-symbol aliases were used for identifier conversion, without human-symbol fallback or inferred remapping. Of the 133 inputs, 125 mapped to STRING proteins and 8 had no exact Gallus gallus alias in STRING v12.0. At a minimum combined interaction score of 0.400 and with no added neighbor nodes, 8 of the 125 mapped proteins were isolated within the input-restricted network, leaving 117 non-isolated PPI nodes. The re-audited network contained 685 undirected interactions. Table S2C-1 provides the complete 133-input traceability chain, and Table S2C-2 retains the PPI topology/core-target record.

Table S2C-1. Complete STRING v12.0 identifier-conversion and 133→117 PPI traceability audit

Input no.	Gallus input	Gallus Ensembl 116 gene ID	Source human	STRING mapping	STRING protein ID	STRING preferred name	Degree	Final PPI status
1	ABCB1LA	ENSGALG00010005222	ABCB1	Mapped	9031.ENSGALP00000050400	ABCB1	2	Retained in 117-node PPI
2	ABL1	ENSGALG00010027607	ABL1	Mapped	9031.ENSGALP00000006154	ABL1	4	Retained in 117-node PPI
3	ACHE	ENSGALG00010000453	ACHE	Mapped	9031.ENSGALP00000074034	ACHE	3	Retained in 117-node PPI
4	ACKR2	ENSGALG00010027403	CYP8B1	Mapped	9031.ENSGALP00000008492	ACKR2	1	Retained in 117-node PPI
5	AHR1A	ENSGALG00010004433	AHR	Unmapped in STRING v12.0	—	—	0	Excluded from PPI
6	AKR1B10	ENSGALG00010013501	AKR1B10	Mapped	9031.ENSGALP00000021315	AKR1B10	0	Mapped but isolated
7	AKT1	ENSGALG00010017364	AKT1	Mapped	9031.ENSGALP00000066725	AKT1	23	Retained in 117-node PPI
8	ALB	ENSGALG00010005070	ALB	Mapped	9031.ENSGALP00000044604	ALB	33	Retained in 117-node PPI
9	ALK	ENSGALG00010017563	ALK	Mapped	9031.ENSGALP00000070314	ALK	6	Retained in 117-node PPI
10	ALOX5	ENSGALG00010023818	ALOX5	Mapped	9031.ENSGALP00000009402	ALOX5	4	Retained in 117-node PPI
11	APP	ENSGALG00010010915	APP	Mapped	9031.ENSGALP00000036014	APP	12	Retained in 117-node PPI
12	BCHE	ENSGALG00010001267	BCHE	Mapped	9031.ENSGALP00000069058	BCHE	3	Retained in 117-node PPI
13	BCL2	ENSGALG00010011843	BCL2	Mapped	9031.ENSGALP00000020984	BCL2	41	Retained in 117-node PPI
14	BCL2L1	ENSGALG00010014066	BCL2L1	Mapped	9031.ENSGALP00000054103	BCL2L1	18	Retained in 117-node PPI
15	BIRC8	ENSGALG00010013281	XIAP	Mapped	9031.ENSGALP00000073811	BIRC8	7	Retained in 117-node PPI
16	BRAF	ENSGALG00010013466	BRAF	Mapped	9031.ENSGALP00000020950	BRAF	20	Retained in 117-node PPI
17	CA2	ENSGALG00010013325	CA2	Mapped	9031.ENSGALP00000056391	CA2	0	Mapped but isolated
18	CCR5	ENSGALG00010017278	CCR5	Mapped	9031.ENSGALP00000053031	CCR5	9	Retained in 117-node PPI
19	COMT	ENSGALG00010024592	COMT	Mapped	9031.ENSGALP00000070206	COMT	6	Retained in 117-node PPI
20	COX2	ENSGALG00010000020	MT-CO2	Mapped	9031.ENSGALP00000058371	MT-CO2	0	Mapped but isolated
21	CREBBP	ENSGALG00010016508	CREBBP	Mapped	9031.ENSGALP00000054729	CREBBP	17	Retained in 117-node PPI
22	CSF1R	ENSGALG00010016804	CSF1R	Mapped	9031.ENSGALP00000009176	CSF1R	8	Retained in 117-node PPI
23	CTNNB1	ENSGALG00010016258	CTNNB1	Mapped	9031.ENSGALP00000045406	CTNNB1	30	Retained in 117-node PPI
24	CXCL12	ENSGALG00010021867	CXCL12	Mapped	9031.ENSGALP00000047558	CXCL12	20	Retained in 117-node PPI
25	CYP1A1	ENSGALG00010021122	CYP1A1	Mapped	9031.ENSGALP00000039219	CYP1A1	8	Retained in 117-node PPI
26	CYP1A2	ENSGALG00010021117	CYP1A2	Mapped	9031.ENSGALP00000002016	CYP1A2	12	Retained in 117-node PPI
27	CYP1B1	ENSGALG00010007388	CYP1B1	Mapped	9031.ENSGALP00000048510	CYP1B1	5	Retained in 117-node PPI
28	CYP3A5	ENSGALG00010017603	CYP3A4	Mapped	9031.ENSGALP00000007068	CYP3A5	9	Retained in 117-node PPI

Input no.	Gallus input	Gallus Ensembl 116 gene ID	Source human	STRING mapping	STRING protein ID	STRING preferred name	Degree	Final PPI status
29	DPP4	ENSGALG00010021045	DPP4	Mapped	9031.ENSGALP00000052897	DPP4	5	Retained in 117-node PPI
30	ECE1	ENSGALG00010021126	ECE1	Mapped	9031.ENSGALP00000043889	ECE1	1	Retained in 117-node PPI
31	EGFR	ENSGALG00010007381	EGFR	Mapped	9031.ENSGALP00000053328	EGFR	33	Retained in 117-node PPI
32	EP300	ENSGALG00010012865	EP300	Mapped	9031.ENSGALP00000019537	EP300	18	Retained in 117-node PPI
33	ERBB2	ENSGALG00010024479	ERBB2	Mapped	9031.ENSGALP00000044764	ERBB2	14	Retained in 117-node PPI
34	ESR1	ENSGALG00010005186	ESR1	Mapped	9031.ENSGALP00000073938	ESR1	34	Retained in 117-node PPI
35	ESR2	ENSGALG00010017591	ESR2	Mapped	9031.ENSGALP00000019234	ESR2	14	Retained in 117-node PPI
36	F13A1	ENSGALG00010010923	F13A1	Mapped	9031.ENSGALP00000020857	F13A1	1	Retained in 117-node PPI
37	F3	ENSGALG00010021018	F3	Mapped	9031.ENSGALP00000009003	F3	3	Retained in 117-node PPI
38	FABP2	ENSGALG00010005505	FABP2	Mapped	9031.ENSGALP00000019528	FABP2	3	Retained in 117-node PPI
39	FLT4	ENSGALG00010016867	FLT4	Mapped	9031.ENSGALP00000009174	FLT4	8	Retained in 117-node PPI
40	FOLH1	ENSGALG00010007404	FOLH1	Mapped	9031.ENSGALP00000060843	FOLH1	0	Mapped but isolated
41	GLI1	ENSGALG00010025727	GLI1	Mapped	9031.ENSGALP00000049322	GLI1	4	Retained in 117-node PPI
42	GSK3A	ENSGALG00010025103	GSK3B	Mapped	9031.ENSGALP00000066184	GSK3B	18	Retained in 117-node PPI
43	GSR	ENSGALG00010008016	GSR	Mapped	9031.ENSGALP00000047092	GSR	7	Retained in 117-node PPI
44	HDAC2	ENSGALG00010013392	HDAC2	Mapped	9031.ENSGALP00000057637	HDAC2	9	Retained in 117-node PPI
45	HIF1A	ENSGALG00010017810	HIF1A	Mapped	9031.ENSGALP00000019338	HIF1A	39	Retained in 117-node PPI
46	HMOX1	ENSGALG00010015155	HMOX1	Mapped	9031.ENSGALP00000036970	HMOX1	19	Retained in 117-node PPI
47	HSD11B1A	ENSGALG00010027203	HSD11B1	Unmapped in STRING v12.0	—	—	0	Excluded from PPI
48	HSD11B2	ENSGALG00010021624	HSD11B2	Mapped	9031.ENSGALP00000042090	HSD11B2	2	Retained in 117-node PPI
49	HTR3A	ENSGALG00010024453	HTR3A	Mapped	9031.ENSGALP00000049060	HTR3A	0	Mapped but isolated
50	IGF1R	ENSGALG00010018730	IGF1R	Mapped	9031.ENSGALP00000056946	IGF1R	19	Retained in 117-node PPI
51	IKBKB	ENSGALG00010017955	IKBKB	Mapped	9031.ENSGALP00000051668	IKBKB	4	Retained in 117-node PPI
52	JAK1	ENSGALG00010024493	JAK1	Mapped	9031.ENSGALP00000017948	JAK1	14	Retained in 117-node PPI
53	JAK2	ENSGALG00010012833	JAK2	Mapped	9031.ENSGALP00000074116	JAK2	23	Retained in 117-node PPI
54	JAK3	ENSGALG00010027619	JAK3	Unmapped in STRING v12.0	—	—	0	Excluded from PPI
55	JUN	ENSGALG00010026886	JUN	Mapped	9031.ENSGALP00000037247	JUN	28	Retained in 117-node PPI
56	KCNA1	ENSGALG00010024400	KCNA1	Unmapped in STRING v12.0	—	—	0	Excluded from PPI
57	KDR	ENSGALG00010013498	KDR	Mapped	9031.ENSGALP00000022522	KDR	21	Retained in 117-node PPI
58	LCK	ENSGALG00010017442	LCK	Mapped	9031.ENSGALP00000000634	LCK	10	Retained in 117-node PPI
59	LRRK2	ENSGALG00010009361	LRRK2	Mapped	9031.ENSGALP00000052474	LRRK2	3	Retained in 117-node PPI
60	LTA4H	ENSGALG00010011903	LTA4H	Mapped	9031.ENSGALP00000049704	LTA4H	5	Retained in 117-node PPI
61	LTB4R	ENSGALG00010003212	LTB4R	Mapped	9031.ENSGALP00000054475	LTB4R	1	Retained in 117-node PPI
62	MAP2K1	ENSGALG00010018000	MAP2K1	Mapped	9031.ENSGALP00000050365	MAP2K1	9	Retained in 117-node PPI
63	MAPK14	ENSGALG00010027041	MAPK14	Mapped	9031.ENSGALP00000052690	MAPK14	10	Retained in 117-node PPI
64	MAPK3	ENSGALG00010024358	MAPK1	Mapped	9031.ENSGALP00000002278	MAPK3	17	Retained in 117-node PPI
65	MAPT	ENSGALG00010024833	MAPT	Mapped	9031.ENSGALP00000072691	MAPT	6	Retained in 117-node PPI
66	MEP1B	ENSGALG00010004061	MEP1B	Mapped	9031.ENSGALP00000060193	MEP1B	1	Retained in 117-node PPI
67	MET	ENSGALG00010001514	MET	Mapped	9031.ENSGALP00000054618	MET	19	Retained in 117-node PPI
68	MIF	ENSGALG00010026095	MIF	Mapped	9031.ENSGALP00000010214	MIF	2	Retained in 117-node PPI
69	MMP1	ENSGALG00010007571	MMP1	Mapped	9031.ENSGALP00000027716	MMP1	5	Retained in 117-node PPI
70	MMP2	ENSGALG00010025414	MMP2	Mapped	9031.ENSGALP00000005656	MMP2	14	Retained in 117-node PPI
71	MMP9	ENSGALG00010015061	MMP9	Mapped	9031.ENSGALP00000068659	MMP9	33	Retained in 117-node PPI
72	MTOR	ENSGALG00010021746	MTOR	Mapped	9031.ENSGALP00000005274	MTOR	15	Retained in 117-node PPI
73	NFE2L2	ENSGALG00010024107	NFE2L2	Mapped	9031.ENSGALP00000074218	NFE2L2	15	Retained in 117-node PPI
74	NFKB1	ENSGALG00010005476	NFKB1	Mapped	9031.ENSGALP00000036814	NFKB1	10	Retained in 117-node PPI
75	NOS1	ENSGALG00010000576	NOS1	Mapped	9031.ENSGALP00000057794	NOS1	3	Retained in 117-node PPI
76	NOS2	ENSGALG00010029552	NOS2	Mapped	9031.ENSGALP00000058328	NOS2	10	Retained in 117-node PPI
77	NOS3	ENSGALG00010027175	NOS3	Mapped	9031.ENSGALP00000073760	NOS3	3	Retained in 117-node PPI
78	NR1H3	ENSGALG00010022650	NR1H3	Mapped	9031.ENSGALP00000073715	NR1H3	4	Retained in 117-node PPI
79	NR1H4	ENSGALG00010011566	NR1H4	Mapped	9031.ENSGALP00000018906	NR1H4	9	Retained in 117-node PPI
80	NR1I3	ENSGALG00010027496	NR1I3	Unmapped in STRING v12.0	—	—	0	Excluded from PPI
81	NR3C1	ENSGALG00010016758	NR3C1	Mapped	9031.ENSGALP00000052544	NR3C1	11	Retained in 117-node PPI
82	NR3C2	ENSGALG00010006735	NR3C2	Mapped	9031.ENSGALP00000058137	NR3C2	2	Retained in 117-node PPI
83	NT5E	ENSGALG00010004224	NT5E	Mapped	9031.ENSGALP00000025482	NT5E	1	Retained in 117-node PPI
84	NTRK1	ENSGALG00010028736	NTRK1	Mapped	9031.ENSGALP00000021606	NTRK1	6	Retained in 117-node PPI
85	ODC	ENSGALG00010003604	ODC1	Mapped	9031.ENSGALP00000026476	ODC1	0	Mapped but isolated
86	PCASP1	ENSGALG00010010352	MALT1	Mapped	9031.ENSGALP00000046622	MALT1	5	Retained in 117-node PPI

Input no.	Gallus input	Gallus Ensembl 116 gene ID	Source human	STRING mapping	STRING protein ID	STRING preferred name	Degree	Final PPI status
87	PCSK9	ENSGALG00010022791	PCSK9	Mapped	9031.ENSGALP00000071049	PCSK9	1	Retained in 117-node PPI
88	PDGFRB	ENSGALG00010016785	PDGFRB	Mapped	9031.ENSGALP00000043875	PDGFRB	12	Retained in 117-node PPI
89	PIK3CA	ENSGALG00010018296	PIK3CA	Mapped	9031.ENSGALP00000014504	PIK3CA	28	Retained in 117-node PPI
90	PIK3CB	ENSGALG00010025125	PIK3CB	Mapped	9031.ENSGALP00000008740	PIK3CB	21	Retained in 117-node PPI
91	PIK3CG	ENSGALG00010009050	PIK3CG	Mapped	9031.ENSGALP00000013101	PIK3CG	5	Retained in 117-node PPI
92	PLA2G4A	ENSGALG00010021027	PLA2G4A	Mapped	9031.ENSGALP00000008107	PLA2G4A	6	Retained in 117-node PPI
93	PLA2G5	ENSGALG00010020717	PLA2G2A	Mapped	9031.ENSGALP00000022885	PLA2G5	5	Retained in 117-node PPI
94	PLG	ENSGALG00010007226	PLG	Mapped	9031.ENSGALP00000006818	PLG	13	Retained in 117-node PPI
95	PPARA	ENSGALG00010023058	PPARA	Mapped	9031.ENSGALP00000053048	PPARA	11	Retained in 117-node PPI
96	PPARD	ENSGALG00010027545	PPARD	Mapped	9031.ENSGALP00000072840	PPARD	3	Retained in 117-node PPI
97	PPARG	ENSGALG00010027917	PPARG	Mapped	9031.ENSGALP00000067699	PPARG	27	Retained in 117-node PPI
98	PRKCA	ENSGALG00010029209	PRKCA	Mapped	9031.ENSGALP00000041561	PRKCA	7	Retained in 117-node PPI
99	PRKCD	ENSGALG00010028105	PRKCD	Mapped	9031.ENSGALP00000003132	PRKCD	8	Retained in 117-node PPI
100	PRKCQ	ENSGALG00010005409	PRKCQ	Unmapped in STRING v12.0	—	—	0	Excluded from PPI
101	PRSS2	ENSGALG00010023541	PRSS1	Mapped	9031.ENSGALP00000055103	PRSS2	6	Retained in 117-node PPI
102	PTGER2	ENSGALG00010017954	PTGER2	Mapped	9031.ENSGALP00000020216	PTGER2	1	Retained in 117-node PPI
103	PTGER4	ENSGALG00010014350	PTGER4	Mapped	9031.ENSGALP00000065250	PTGER4	3	Retained in 117-node PPI
104	PTGS1	ENSGALG00010028372	PTGS1	Mapped	9031.ENSGALP00000053876	PTGS1	6	Retained in 117-node PPI
105	PTGS2	ENSGALG00010021038	PTGS2	Mapped	9031.ENSGALP00000054566	PTGS2	30	Retained in 117-node PPI
106	PTK2	ENSGALG00010009159	PTK2	Mapped	9031.ENSGALP00000071114	PTK2	19	Retained in 117-node PPI
107	PTPN11	ENSGALG00010020701	PTPN11	Mapped	9031.ENSGALP00000007692	PTPN11	19	Retained in 117-node PPI
108	PTPN2	ENSGALG00010001185	PTPN2	Mapped	9031.ENSGALP00000022449	PTPN2	6	Retained in 117-node PPI
109	PTPRC	ENSGALG00010012352	PTPRC	Mapped	9031.ENSGALP00000055398	PTPRC	10	Retained in 117-node PPI
110	RORC	ENSGALG00010028041	RORC	Mapped	9031.ENSGALP00000049570	RORC	1	Retained in 117-node PPI
111	SI	ENSGALG00010001273	MGAM	Mapped	9031.ENSGALP00000015467	SI	0	Mapped but isolated
112	SIRT1	ENSGALG00010009252	SIRT1	Mapped	9031.ENSGALP00000004350	SIRT1	29	Retained in 117-node PPI
113	SLC10A1	ENSGALG00010021491	SLC10A1	Unmapped in STRING v12.0	—	—	0	Excluded from PPI
114	SLC1A1	ENSGALG00010011937	SLC1A1	Mapped	9031.ENSGALP00000016539	SLC1A1	1	Retained in 117-node PPI
115	SLC5A1	ENSGALG00010026633	SLC5A1	Mapped	9031.ENSGALP00000010870	SLC5A1	2	Retained in 117-node PPI
116	SLC6A4	ENSGALG00010029709	SLC6A4	Mapped	9031.ENSGALP00000006742	SLC6A4	2	Retained in 117-node PPI
117	SNCA	ENSGALG00010008368	SNCA	Mapped	9031.ENSGALP00000067652	SNCA	10	Retained in 117-node PPI
118	SRC	ENSGALG00010021638	SRC	Mapped	9031.ENSGALP00000006117	SRC	40	Retained in 117-node PPI
119	STAT1	ENSGALG00010013934	STAT1	Mapped	9031.ENSGALP00000046362	STAT1	31	Retained in 117-node PPI
120	STAT3	ENSGALG00010026098	STAT3	Mapped	9031.ENSGALP00000005161	STAT3	53	Retained in 117-node PPI
121	SYK	ENSGALG00010009340	SYK	Mapped	9031.ENSGALP00000024509	SYK	12	Retained in 117-node PPI
122	TBXAS1	ENSGALG00010013152	TBXAS1	Mapped	9031.ENSGALP00000069343	TBXAS1	3	Retained in 117-node PPI
123	TEK	ENSGALG00010006538	TEK	Mapped	9031.ENSGALP00000072046	TEK	4	Retained in 117-node PPI
124	TERT	ENSGALG00010008150	TERT	Mapped	9031.ENSGALP00000021506	TERT	11	Retained in 117-node PPI
125	TGFBR1	ENSGALG00010007951	TGFBR1	Mapped	9031.ENSGALP00000054311	TGFBR1	2	Retained in 117-node PPI
126	TLR4	ENSGALG00010028914	TLR4	Mapped	9031.ENSGALP00000011319	TLR4	32	Retained in 117-node PPI
127	TRPA1	ENSGALG00010002600	TRPA1	Mapped	9031.ENSGALP00000053618	TRPA1	2	Retained in 117-node PPI
128	TRPV1	ENSGALG00010029746	TRPV1	Mapped	9031.ENSGALP00000007393	TRPV1	4	Retained in 117-node PPI
129	TTR	ENSGALG00010006069	TTR	Mapped	9031.ENSGALP00000024388	TTR	6	Retained in 117-node PPI
130	TYR	ENSGALG00010007864	TYR	Mapped	9031.ENSGALP00000027812	TYR	3	Retained in 117-node PPI
131	VDR	ENSGALG00010024416	VDR	Mapped	9031.ENSGALP00000071161	VDR	0	Mapped but isolated
132	XDH	ENSGALG00010018628	XDH	Mapped	9031.ENSGALP00000014144	XDH	4	Retained in 117-node PPI
133	Not assigned in Ensembl 116	ENSGALG00010027949	GBA1	Unmapped in STRING v12.0	—	—	0	Excluded from PPI

Identifier-normalization note. The reviewer-highlighted symbol differences were verified directly in STRING v12.0: ABCB1LA (ENSGALG00010005222) maps to 9031.ENSGALP00000050400 with STRING preferred name ABCB1; GSK3A (ENSGALG00010025103) maps to 9031.ENSGALP00000066184 with preferred name GSK3B; and PCASP1 (ENSGALG00010010352) maps to 9031.ENSGALP00000046622 with preferred name MALT1. These are STRING preferred-name/alias normalizations rather than substitutions of different biological inputs. The 133-input accounting is: 117 retained non-isolated PPI nodes + 8 mapped-but-isolated inputs + 8 inputs without an exact Gallus gallus STRING v12.0 alias = 133.

Table S2C-2. Chicken-specific STRING PPI topology/core-target record for the 117 non-isolated nodes

No.	PPI gene (STRING preferred name)	STRING protein ID	Degree	Source input no.
1	STAT3	9031.ENSGALP00000005161	53	120
2	BCL2	9031.ENSGALP00000020984	41	13
3	SRC	9031.ENSGALP00000006117	40	118
4	HIF1A	9031.ENSGALP00000019338	39	45
5	ESR1	9031.ENSGALP00000073938	34	34

No.	PPI gene (STRING preferred name)	STRING protein ID	Degree	Source input no.
6	ALB	9031.ENSGALP00000044604	33	8
7	EGFR	9031.ENSGALP00000053328	33	31
8	MMP9	9031.ENSGALP00000068659	33	71
9	TLR4	9031.ENSGALP00000011319	32	126
10	STAT1	9031.ENSGALP00000046362	31	119
11	CTNNB1	9031.ENSGALP00000045406	30	23
12	PTGS2	9031.ENSGALP00000054566	30	105
13	SIRT1	9031.ENSGALP00000004350	29	112
14	JUN	9031.ENSGALP00000037247	28	55
15	PIK3CA	9031.ENSGALP00000014504	28	89
16	PPARG	9031.ENSGALP00000067699	27	97
17	AKT1	9031.ENSGALP00000066725	23	7
18	JAK2	9031.ENSGALP00000074116	23	53
19	KDR	9031.ENSGALP00000022522	21	57
20	PIK3CB	9031.ENSGALP00000008740	21	90
21	BRAF	9031.ENSGALP00000020950	20	16
22	CXCL12	9031.ENSGALP00000047558	20	24
23	HMOX1	9031.ENSGALP00000036970	19	46
24	IGF1R	9031.ENSGALP00000056946	19	50
25	MET	9031.ENSGALP00000054618	19	67
26	PTK2	9031.ENSGALP00000071114	19	106
27	PTPN11	9031.ENSGALP00000007692	19	107
28	BCL2L1	9031.ENSGALP00000054103	18	14
29	EP300	9031.ENSGALP00000019537	18	32
30	GSK3B	9031.ENSGALP00000066184	18	42
31	CREBBP	9031.ENSGALP00000054729	17	21
32	MAPK3	9031.ENSGALP00000002278	17	64
33	MTOR	9031.ENSGALP00000005274	15	72
34	NFE2L2	9031.ENSGALP00000074218	15	73
35	ERBB2	9031.ENSGALP00000044764	14	33
36	ESR2	9031.ENSGALP00000019234	14	35
37	JAK1	9031.ENSGALP00000017948	14	52
38	MMP2	9031.ENSGALP00000005656	14	70
39	PLG	9031.ENSGALP00000006818	13	94
40	APP	9031.ENSGALP00000036014	12	11
41	CYP1A2	9031.ENSGALP00000002016	12	26
42	PDGFRB	9031.ENSGALP00000043875	12	88
43	SYK	9031.ENSGALP00000024509	12	121
44	NR3C1	9031.ENSGALP00000052544	11	81
45	PPARA	9031.ENSGALP00000053048	11	95
46	TERT	9031.ENSGALP00000021506	11	124
47	LCK	9031.ENSGALP00000000634	10	58
48	MAPK14	9031.ENSGALP00000052690	10	63
49	NFKB1	9031.ENSGALP00000036814	10	74
50	NOS2	9031.ENSGALP00000058328	10	76
51	PTPRC	9031.ENSGALP00000055398	10	109
52	SNCA	9031.ENSGALP00000067652	10	117
53	CCR5	9031.ENSGALP00000053031	9	18
54	CYP3A5	9031.ENSGALP00000007068	9	28
55	HDAC2	9031.ENSGALP00000057637	9	44
56	MAP2K1	9031.ENSGALP00000050365	9	62
57	NR1H4	9031.ENSGALP00000018906	9	79
58	CSF1R	9031.ENSGALP00000009176	8	22
59	CYP1A1	9031.ENSGALP00000039219	8	25
60	FLT4	9031.ENSGALP00000009174	8	39
61	PRKCD	9031.ENSGALP00000003132	8	99
62	BIRC8	9031.ENSGALP00000073811	7	15
63	GSR	9031.ENSGALP00000047092	7	43
64	PRKCA	9031.ENSGALP00000041561	7	98
65	ALK	9031.ENSGALP00000070314	6	9
66	COMT	9031.ENSGALP00000070206	6	19
67	MAPT	9031.ENSGALP00000072691	6	65
68	NTRK1	9031.ENSGALP00000021606	6	84
69	PLA2G4A	9031.ENSGALP00000008107	6	92
70	PRSS2	9031.ENSGALP00000055103	6	101
71	PTGS1	9031.ENSGALP00000053876	6	104
72	PTPN2	9031.ENSGALP00000022449	6	108
73	TTR	9031.ENSGALP00000024388	6	129
74	CYP1B1	9031.ENSGALP00000048510	5	27
75	DPP4	9031.ENSGALP00000052897	5	29
76	LTA4H	9031.ENSGALP00000049704	5	60
77	MALT1	9031.ENSGALP00000046622	5	86
78	MMP1	9031.ENSGALP00000027716	5	69
79	PIK3CG	9031.ENSGALP00000013101	5	91
80	PLA2G5	9031.ENSGALP00000022885	5	93
81	ABL1	9031.ENSGALP00000006154	4	2
82	ALOX5	9031.ENSGALP00000009402	4	10
83	GLI1	9031.ENSGALP00000049322	4	41
84	IKBKB	9031.ENSGALP00000051668	4	51
85	NR1H3	9031.ENSGALP00000073715	4	78

No.	PPI gene (STRING preferred name)	STRING protein ID	Degree	Source input no.
86	TEK	9031.ENSGALP00000072046	4	123
87	TRPV1	9031.ENSGALP00000007393	4	128
88	XDH	9031.ENSGALP00000014144	4	132
89	ACHE	9031.ENSGALP00000074034	3	3
90	BCHE	9031.ENSGALP00000069058	3	12
91	F3	9031.ENSGALP00000009003	3	37
92	FABP2	9031.ENSGALP00000019528	3	38
93	LRRK2	9031.ENSGALP00000052474	3	59
94	NOS1	9031.ENSGALP00000057794	3	75
95	NOS3	9031.ENSGALP00000073760	3	77
96	PPARD	9031.ENSGALP00000072840	3	96
97	PTGER4	9031.ENSGALP00000065250	3	103
98	TBXAS1	9031.ENSGALP00000069343	3	122
99	TYR	9031.ENSGALP00000027812	3	130
100	ABCB1	9031.ENSGALP00000050400	2	1
101	HSD11B2	9031.ENSGALP00000042090	2	48
102	MIF	9031.ENSGALP00000010214	2	68
103	NR3C2	9031.ENSGALP00000058137	2	82
104	SLC5A1	9031.ENSGALP00000010870	2	115
105	SLC6A4	9031.ENSGALP00000006742	2	116
106	TGFBR1	9031.ENSGALP00000054311	2	125
107	TRPA1	9031.ENSGALP00000053618	2	127
108	ACKR2	9031.ENSGALP00000008492	1	4
109	ECE1	9031.ENSGALP00000043889	1	30
110	F13A1	9031.ENSGALP00000020857	1	36
111	LTB4R	9031.ENSGALP00000054475	1	61
112	MEP1B	9031.ENSGALP00000060193	1	66
113	NT5E	9031.ENSGALP00000025482	1	83
114	PCSK9	9031.ENSGALP00000071049	1	87
115	PTGER2	9031.ENSGALP00000020216	1	102
116	RORC	9031.ENSGALP00000049570	1	110
117	SLC1A1	9031.ENSGALP00000016539	1	114

The STRING v12.0 re-audit of the 133 representative Gallus gallus inputs yielded 117 non-isolated PPI nodes and 685 undirected interactions at a minimum combined score of 0.400. STRING preferred names may differ from the Ensembl-derived chicken input symbols because of database alias normalization. Table S2C-1 provides the complete 133→117 traceability audit, while Table S2C-2 lists the 117 retained STRING nodes, protein identifiers, and Degree values calculated directly from the re-audited 685-edge network. This 685-edge reconstruction is retained as an identifier-traceability sensitivity audit. The primary PPI/core-target analysis and Figure 4 in the manuscript remain the archived 117-node/683-edge network from which the original cytoHubba and MCODE outputs were generated; the 685-edge traceability re-audit is not retrospectively substituted into that downstream core-target analysis. This distinction prevents mixing topology results from two slightly different network reconstructions.

Table S2D. Archived four-algorithm cytoHubba Top20 ranking and deterministic core-target selection from the primary 117-node/683-edge chicken-specific STRING PPI network

Rank	Degree	MCC	Betweenness	Closeness
1	STAT3	STAT3	PTGS2	ECE1
2	BCL2	BCL2	STAT3	MEP1B
3	SRC	HIF1A	ALB	STAT3
4	HIF1A	MMP9	PPARG	BCL2
5	EGFR	PPARG	SRC	HIF1A
6	ESR1	STAT1	TLR4	SRC
7	MMP9	PTGS2	MMP9	MMP9
8	ALB	ALB	ESR1	ESR1
9	TLR4	SRC	HIF1A	TLR4
10	STAT1	EGFR	CTNNB1	ALB
11	CTNNB1	ESR1	BCL2	PTGS2
12	PTGS2	JUN	AKT1	STAT1
13	SIRT1	BCL2L1	NR3C1	EGFR
14	JUN	TLR4	FABP2	SIRT1
15	PIK3CA	SIRT1	EGFR	JUN
16	PPARG	HMOX1	CXCL12	PPARG
17	AKT1	JAK2	SIRT1	CTNNB1
18	JAK2	CTNNB1	JUN	JAK2
19	KDR	CREBBP	PIK3CA	BCL2L1
20	PIK3CB	MET	STAT1	HMOX1

Four-algorithm Top20 intersection (n = 15): STAT3, BCL2, SRC, HIF1A, EGFR, ESR1, MMP9, ALB, TLR4, STAT1, CTNNB1, PTGS2, SIRT1, JUN, PPARG.

Final core targets after intersection with MCODE core modules (n = 13): BCL2, SRC, HIF1A, EGFR, ESR1, MMP9, ALB, TLR4, STAT1, PTGS2, SIRT1, JUN, PPARG. Deterministic receptor-selection rule: within these 13 final core targets, the Degree distribution was 41, 40, 39, 33, 33, 33, 32, 32, 31, 30, 29, 28, and 27, giving Q3 = 33. Targets with Degree >= Q3 were defined as the high-connectivity core tier and advanced to docking, yielding BCL2, SRC, HIF1A, MMP9, ESR1, and EGFR (n = 6). Degree is used here only as a topological prioritization metric after the multi-algorithm/MCODE consensus step and not as evidence of biological importance or therapeutic relevance.

Table S2E. Chicken receptor structures and AutoDock Vina search-space parameters

The six receptors used for molecular docking were Gallus gallus-specific structures or structure models. Five targets were represented by AlphaFold models, whereas SRC was represented by the experimentally determined PDB structure 2PTK. Target-specific receptor provenance and the original receptor-specific whole-protein search boxes are reported below. To address the reviewer's reproducibility concern without retrospectively redefining undocumented settings of the archived docking run, all 60 ligand-target combinations were additionally rerun under a standardized, fully recorded protocol. This rerun is reported as a supplementary reproducibility/sensitivity assessment; the manuscript's original docking figures and archived score presentation remain the primary presentation of the original analysis. The ligand panel consists of the ten compounds present in the archived docking workflow. The original compound-selection rule was not preserved, and the archived ten-compound panel is not identical to the Degree > Q3 high-connectivity tier of the revised 38-compound network; therefore, the supplementary audit does not retrospectively describe those ten ligands as an objectively top-ranked set. Complete 38-compound topology and the explicit mismatch are documented in Supplementary Table S1B.

Table S2E-1. Receptor provenance

Target	Species	Structure type	Structure/model ID	Chain
BCL2	Gallus gallus	AlphaFold predicted model	AF-Q00709-F1-model_v6	A
EGFR	Gallus gallus	AlphaFold predicted model	AF-P13387-F1-model_v6	A
ESR1	Gallus gallus	AlphaFold predicted model	AF-P06212-F1-model_v6	A
HIF1A	Gallus gallus	AlphaFold predicted model	AF-Q9YIB9-F1-model_v6	A
MMP9	Gallus gallus	AlphaFold predicted model	AF-A0A8V0YXL9-F1-model_v6	A
SRC	Gallus gallus	Experimental PDB structure	2PTK	A

Table S2E-2. Receptor-specific docking grid parameters

Target	Center X	Center Y	Center Z	Size X	Size Y	Size Z	Grid spacing (Å)
BCL2	15.3285	11.1415	2.7155	89.679	52.879	61.975	0.375
EGFR	-4.0060	32.0785	24.9300	96.136	141.353	162.760	0.375
ESR1	-3.8725	-11.4845	7.4985	123.343	103.769	127.173	0.375
HIF1A	1.2120	13.7100	4.4085	131.740	118.896	122.969	0.375
MMP9	-12.4250	1.4320	-1.3750	138.154	97.654	97.620	0.375
SRC	23.9850	2.7560	33.2780	63.304	58.832	64.540	0.375

Standardized reproducibility rerun. All 60 ligand-target combinations were rerun with AutoDock Vina v1.2.7 using scoring = vina, exhaustiveness = 50, random seed = 1000, CPU = 4, num_modes = 9, energy_range = 3 kcal/mol, and grid spacing = 0.375 Å. The six exact archived Gallus gallus receptor PDBQT files and receptor-encompassing grid centers/box dimensions were held fixed. Because the historical upstream receptor-preparation pH was not preserved, no retrospective receptor protonation state was inferred; the fixed receptor PDBQT hydrogen/charge states are therefore the explicit receptor inputs for the reproducibility rerun. Ligand microspecies were standardized at pH 7.4 and partial charges were assigned with the RDKit 2025.09.4 Gasteiger procedure; caffeic acid was represented as monoanionic caffeate (formal charge -1), whereas the other nine ligands were represented as neutral parent species. Archived three-dimensional ligand coordinates and torsion trees were retained, and no additional pre-docking energy minimization was introduced. Protein-only receptor PDBQT inputs were used; no cofactors, metals, or waters were added. The lowest-scoring pose among nine modes was retained for the rerun comparison. These standardized settings apply only to the additional reproducibility rerun and are not retrospectively attributed to the original docking calculation. An independent ligand-input reconstruction check confirmed the expected net formal charge for all 10 standardized ligands and a maximum coordinate-mapping deviation of 0.0008 A relative to the archived ligand coordinates.

Table S2E-3. Receptor structural completeness and preprocessing audit for the standardized docking rerun

Target	Structure/model	Method / resolution	Chain / scope	Missing residues / reconstruction	Cofactor, metal, water, or modified-residue treatment
BCL2	AF-Q00709-F1-model_v6	AlphaFold predicted model	A; Full-length model, residues 1-233	No modeled sequence gaps in supplied AlphaFold structure	Protein-only receptor PDBQT; no water/cofactor/metal added
EGFR	AF-P13387-F1-model_v6	AlphaFold predicted model	A; Supplied model, residues 1-703	No modeled sequence gaps in supplied AlphaFold structure	Protein-only receptor PDBQT; no water/cofactor/metal added
ESR1	AF-P06212-F1-model_v6	AlphaFold predicted model	A; Full-length model, residues 1-589	No modeled sequence gaps in supplied AlphaFold structure	Protein-only receptor PDBQT; no water/cofactor/metal added
HIF1A	AF-Q9YIB9-F1-model_v6	AlphaFold predicted model	A; Full-length model, residues 1-811	No modeled sequence gaps in supplied AlphaFold structure	Protein-only receptor PDBQT; no water/cofactor/metal added
MMP9	AF-A0A8V0YXL9-F1-model_v6	AlphaFold predicted model	A; Full-length model, residues 1-688	No modeled sequence gaps in supplied AlphaFold structure	Protein-only receptor PDBQT; no catalytic metal/cofactor/water was added in this preserved preparation
SRC	PDB 2PTK	X-ray diffraction; 2.35 Å	A; SH3/SH2/kinase/C-terminal-tail fragment; source numbering 81-533	Source PDB missing 81-82, 300-303, 408-423, and 529-533; none reconstructed	Cleaned receptor omits PTR527 and crystallographic waters; no cofactor/metal/water retained

Table S2E-4. Ligand microspecies and charge-preparation audit for the standardized rerun

PubChem CID	Compound	Standardized pH 7.4 microspecies	Formal charge	Partial-charge procedure	Geometry / minimization
8103	1-Hexanol	1-Hexanol	0	RDKit 2025.09.4 Gasteiger; nonpolar-H charge merged into attached heavy atom	Archived 3D coordinates and torsion tree retained; no additional minimization
8914	Nonan-1-ol	Nonan-1-ol	0	RDKit 2025.09.4 Gasteiger; nonpolar-H charge merged into attached heavy atom	Archived 3D coordinates and torsion tree retained; no additional minimization
10393	2-(4-Hydroxyphenyl)ethanol	2-(4-Hydroxyphenyl)ethanol	0	RDKit 2025.09.4 Gasteiger; nonpolar-H charge merged into attached heavy atom	Archived 3D coordinates and torsion tree retained; no additional minimization
18827	1-Octen-3-ol	1-Octen-3-ol	0	RDKit 2025.09.4 Gasteiger; nonpolar-H charge merged into attached heavy atom	Archived 3D coordinates and torsion tree retained; no additional minimization
447765	(S)-Acetoin	(S)-Acetoin	0	RDKit 2025.09.4 Gasteiger; nonpolar-H charge merged into attached heavy atom	Archived 3D coordinates and torsion tree retained; no additional minimization
689043	Caffeic acid	Caffeate (deprotonated caffeic acid at pH 7.4)	-1	RDKit 2025.09.4 Gasteiger; nonpolar-H charge merged into attached heavy atom	Archived 3D coordinates and torsion tree retained; no additional minimization
5280460	Scopoletin	Scopoletin	0	RDKit 2025.09.4 Gasteiger; nonpolar-H charge merged into attached heavy atom	Archived 3D coordinates and torsion tree retained; no additional minimization
5280507	Sinapyl alcohol	Sinapyl alcohol	0	RDKit 2025.09.4 Gasteiger; nonpolar-H charge merged into attached heavy atom	Archived 3D coordinates and torsion tree retained; no additional minimization
5280802	Sinapaldehyde	Sinapaldehyde	0	RDKit 2025.09.4 Gasteiger; nonpolar-H charge merged into attached heavy atom	Archived 3D coordinates and torsion tree retained; no additional minimization
5283335	2-Nonenal	2-Nonenal	0	RDKit 2025.09.4 Gasteiger; nonpolar-H charge merged into attached heavy atom	Archived 3D coordinates and torsion tree retained; no additional minimization

Table S2E-5. Prespecified AutoDock Vina parameters used for all 60 rerun jobs

Parameter	Rerun value / decision
Software	AutoDock Vina v1.2.7
Scoring function	vina
Exhaustiveness	50
Random seed	1000
CPU	4
Number of modes	9
Energy range	3 kcal/mol
Grid spacing	0.375 Å
Binding-site definition	Receptor-encompassing global ("blind-docking") boxes; target-specific centers and dimensions retained from the archived analysis
Receptor coordinates / charges	Exact archived receptor PDBQT files retained unchanged; receptor-preparation pH not retrospectively inferred
Ligand microspecies	pH 7.4; caffeate -1; all other study ligands neutral
Ligand partial charges	RDKit 2025.09.4 Gasteiger
Ligand geometry / minimization	Archived 3D coordinates and torsion trees retained; no additional pre-docking minimization
Cofactors / metals / waters	None added to the retained receptor PDBQT inputs
Pose selection	Lowest Vina score among 9 modes
Post hoc score cutoff	None
Reproducibility record	Receptor/ligand preparation records, rerun parameters, and complete archived-versus-rerun score pairs are reported directly in Tables S2E-1 to S2E-6. Raw standardized-rerun execution logs and corres nine-pose output files were not preserved in the supplied archive and were therefore not retrospectively fabricated.
Analytical role	Additional reproducibility/sensitivity assessment; does not replace the manuscript's original docking figures or archived score presentation
Concordance with archived scores	Pearson r = 0.981; Spearman rho = 0.985; mean absolute difference = 0.119 kcal/mol

Table S2E-6. Complete archived-versus-standardized-rerun AutoDock Vina score matrix (kcal/mol; each receptor cell reports archived score / standardized-rerun score)

CID	Compound	BCL2	EGFR	ESR1	HIF1A	MMP9	SRC
8103	1-Hexanol	-3.593 / -3.690	-3.864 / -3.871	-4.323 / -4.325	-4.517 / -4.538	-4.847 / -4.819	-4.071 / -4.100
8914	Nonan-1-ol	-3.606 / -3.757	-3.869 / -4.013	-4.732 / -4.891	-4.473 / -4.480	-5.839 / -5.232	-4.770 / -4.653
10393	2-(4-Hydroxyphenyl)ethanol	-4.642 / -4.484	-5.525 / -5.511	-5.921 / -5.897	-5.143 / -5.297	-6.558 / -6.542	-5.518 / -5.515
18827	1-Octen-3-ol	-3.851 / -3.940	-4.536 / -4.573	-5.013 / -5.050	-5.174 / -5.032	-5.694 / -5.620	-4.860 / -4.851
447765	(S)-Acetoin	-3.412 / -3.548	-3.685 / -3.811	-4.080 / -4.079	-3.945 / -3.932	-4.916 / -4.926	-3.874 / -3.869
689043	Caffeic acid	-5.448 / -5.383	-5.848 / -5.909	-6.525 / -6.646	-6.007 / -5.783	-7.689 / -7.399	-6.655 / -6.396
5280460	Scopoletin	-5.292 / -5.300	-6.054 / -6.957	-6.890 / -6.893	-6.020 / -6.456	-7.930 / -7.289	-7.192 / -7.227
5280507	Sinapyl alcohol	-4.792 / -4.765	-5.216 / -5.183	-5.965 / -5.585	-5.494 / -5.514	-6.637 / -6.647	-6.333 / -6.370
5280802	Sinapaldehyde	-4.710 / -4.716	-5.304 / -5.350	-5.804 / -5.517	-5.573 / -5.495	-6.736 / -6.705	-6.165 / -6.199
5283335	2-Nonenal	-3.964 / -4.119	-4.632 / -4.688	-4.623 / -4.814	-4.840 / -4.824	-5.793 / -5.816	-4.898 / -4.684

The standardized rerun was configured to generate nine modes for each of the 60 ligand-target combinations. Best-mode scores ranged from -7.399 to -3.548 kcal/mol and showed strong concordance with the archived docking matrix (Pearson r = 0.981; Spearman rho = 0.985; mean absolute difference = 0.119 kcal/mol). Caffeic acid-MMP9 and scopoletin-ESR1 remained highly ranked (1/60 and 5/60, respectively). For compact traceability, Table S2E-6 now reports the archived and standardized-rerun score side by side for every one of the 60 combinations. The standardized rerun is interpreted as a reproducibility/sensitivity check rather than as a replacement for the original docking figures or archived score presentation. Docking scores are within-protocol computational ranking measures rather than experimentally measured affinities. Same-structure co-crystallized-ligand RMSD redocking was not claimed for the six exact study receptor inputs; this remains an explicit validation limitation.

AlphaFold confidence and binding-region audit. Per-residue pLDDT values were extracted directly from the B-factor field of the five supplied AlphaFold PDB files. For each best-ranked docking pose, receptor heavy-atom residues within 4.0 Å of any ligand heavy atom were treated as the predicted local interaction region. Confidence bands were pLDDT >= 90 (very high), 70 to < 90 (confident), 50 to < 70 (low), and < 50 (very low). No PAE files were available, so no PAE-based structural-confidence claim is made. Local pLDDT is used to evaluate confidence in the sampled receptor geometry and not to assert that a global-box pose identifies a physiological binding pocket. The complete model-wide per-residue pLDDT vectors are encoded directly in the B-factor field of the five archived AlphaFold PDB models; to avoid duplicating more than 3,000 model-wide residue rows, the supplement reports the whole-model summaries in Table S2E-7 and exhaustively lists all pose-contact residues with their pLDDT values in Table S2E-8.

Table S2E-7. AlphaFold whole-model and best-pose local pLDDT confidence summary

Target	AlphaFold model	Whole mean	Whole median	Whole <50	Mean local mean	Mean local min	Local mean >=90	Local mean >=70	Any <50 contact

Target	AlphaFold model	Whole mean	Whole median	Whole <50	Mean local mean	Mean local min	Local mean >=90	Local mean >=70	Any <50 contact
BCL2	BCL2_AF-Q00709-F1-model_v6	74.36	81.88	16.7%	84.21	69.84	4/10	9/10	3/10
EGFR	EGFR_AF-P13387-F1-model_v6	87.70	92.62	4.3%	87.71	77.73	2/10	10/10	0/10
ESR1	ESR1_AF-P06212-F1-model_v6	66.90	76.44	45.5%	97.46	95.59	10/10	10/10	0/10
HIF1A	HIF1A_AF-Q9YIB9-F1-model_v6	60.10	51.22	48.6%	93.24	88.02	10/10	10/10	0/10
MMP9	MMP9_AF-A0A8V0YXL9-F1-model_v6	81.05	87.38	12.1%	89.88	81.20	6/10	10/10	0/10

Across all 50 AlphaFold receptor-ligand best poses, 32/50 had a local mean pLDDT >= 90 and 49/50 had a local mean pLDDT >= 70. Three poses contained at least one pLDDT < 50 contact residue, all involving BCL2. The MD-selected caffeic acid/caffeate-MMP9 pose contacted 13 residues (local mean pLDDT 94.67; minimum 88.19), whereas scopoletin-ESR1 contacted 11 residues (local mean 97.42; minimum 95.31).

Table S2E-8. Complete best-pose local-confidence audit for 50 AlphaFold receptor-ligand combinations

Target	CID	Compound	Vina score	Contacts n	Local mean pLDDT	Local min pLDDT	Contact residues with pLDDT
BCL2	8103	1-Hexanol	-3.690	8	93.94	90.00	HIS121(92.1); PHE124(90.8); VAL125(91.6); GLU129(90.0); TRP170(97.6); GLU173(96.9); TYR174(97.2); HIS178(95.4)
BCL2	8914	Nonan-1-ol	-3.757	7	94.96	90.75	PHE124(90.8); VAL125(91.6); TRP170(97.6); GLU173(96.9); TYR174(97.2); ARG177(95.3); HIS178(95.4)
BCL2	10393	2-(4-Hydroxyphenyl)ethanol	-4.484	8	93.94	90.00	HIS121(92.1); PHE124(90.8); VAL125(91.6); GLU129(90.0); TRP170(97.6); GLU173(96.9); TYR174(97.2); HIS178(95.4)
BCL2	18827	1-Octen-3-ol	-3.940	9	94.09	90.00	HIS121(92.1); PHE124(90.8); VAL125(91.6); GLU129(90.0); TRP170(97.6); GLU173(96.9); TYR174(97.2); ARG177(95.3); HIS178(95.4)
BCL2	447765	(S)-Acetoin	-3.548	8	85.75	64.69	ARG12(93.4); LEU16(95.4); ALA32(76.7); GLY33(67.1); GLU34(64.7); ASP165(94.9); ALA168(97.1); THR169(96.8)
BCL2	689043	Caffeic acid	-5.383	6	63.92	41.50	GLY5(41.5); ARG6(52.4); ARG7(52.3); TYR9(72.2); ARG81(71.5); GLN184(93.5)
BCL2	5280460	Scopoletin	-5.300	10	81.95	67.94	LYS22(94.9); GLN25(92.6); ARG26(90.7); SER99(83.8); ARG100(84.8); GLN103(74.8); PHE106(67.9); ALA107(71.8); SER110(68.9); VAL150(89.2)
BCL2	5280507	Sinapyl alcohol	-4.765	9	74.11	49.81	ARG123(86.3); ALA126(86.6); GLU129(90.0); GLU130(84.6); ARG133(89.9); LEU217(65.5); VAL218(60.8); GLY221(53.5); ALA222(49.8)
BCL2	5280802	Sinapaldehyde	-4.716	8	72.12	49.81	ARG123(86.3); ALA126(86.6); GLU130(84.6); ARG133(89.9); LEU217(65.5); VAL218(60.8); GLY221(53.5); ALA222(49.8)
BCL2	5283335	2-Nonenal	-4.119	9	87.30	63.88	ASP10(83.9); ARG12(93.4); GLU13(92.2); LEU16(95.4); LYS17(94.1); HIS20(95.8); TRP30(90.2); ALA32(76.7); ASP35(63.9)
EGFR	8103	1-Hexanol	-3.871	9	86.90	77.69	THR40(86.1); ASN41(83.8); ASN42(81.0); ILE347(90.9); ASP375(93.7); PHE411(95.2); LYS438(88.1); GLN439(85.6); HIS440(77.7)
EGFR	8914	Nonan-1-ol	-4.013	8	90.12	85.31	LEU356(85.3); SER357(92.2); ASN359(94.1); LEU379(93.3); VAL381(90.3); ASP386(88.1); THR389(86.1); THR391(91.7)
EGFR	10393	2-(4-Hydroxyphenyl)ethanol	-5.511	12	86.93	77.69	GLY39(88.8); THR40(86.1); ASN41(83.8); ASN42(81.0); SER69(90.1); ARG316(84.5); ASP375(93.7); PHE411(95.2); THR437(88.6); LYS438(88.1); GLN439(85.6); HIS440(77.7)
EGFR	18827	1-Octen-3-ol	-4.573	9	88.07	81.00	THR40(86.1); ASN41(83.8); ASN42(81.0); ILE347(90.9); LEU356(85.3); ASP375(93.7); PHE411(95.2); THR437(88.6); LYS438(88.1)
EGFR	447765	(S)-Acetoin	-3.811	7	85.92	63.62	ASN42(81.0); LEU47(63.6); LEU356(85.3); SER377(94.9); PHE411(95.2); LEU413(95.8); GLN439(85.6)
EGFR	689043	Caffeic acid	-5.909	9	94.15	88.38	GLU431(96.4); ARG458(92.7); SER459(95.5); LYS461(93.6); PHE528(94.5); CYS530(94.7); CYS533(95.2); LYS540(88.4); CYS542(96.4)
EGFR	5280460	Scopoletin	-6.957	14	86.54	77.69	GLN38(85.2); GLY39(88.8); THR40(86.1); ASN41(83.8); ASN42(81.0); SER69(90.1); ARG316(84.5); GLY348(83.2); ASP375(93.7); PHE411(95.2); THR437(88.6); LYS438(88.1); GLN439(85.6); HIS440(77.7)
EGFR	5280507	Sinapyl alcohol	-5.183	11	80.46	59.66	LEU163(93.4); CYS164(93.2); ASN165(92.8); ASP167(91.1); ALA188(75.2); SER189(64.1); ASN190(59.7); LEU191(67.5); SER192(68.4); CYS194(88.9); PRO195(90.7)
EGFR	5280802	Sinapaldehyde	-5.350	7	88.83	85.31	ILE355(86.3); LEU356(85.3); ASN359(94.1); VAL381(90.3); ASP386(88.1); THR389(86.1); THR391(91.7)
EGFR	5283335	2-Nonenal	-4.688	10	89.22	81.00	THR40(86.1); ASN41(83.8); ASN42(81.0); ILE347(90.9); LEU356(85.3); ASP375(93.7); SER377(94.9); PHE411(95.2); LEU413(95.8); GLN439(85.6)
ESR1	8103	1-Hexanol	-4.325	7	97.53	95.81	LEU340(95.8); LEU343(96.7); GLU347(97.4); LEU381(98.2); LEU385(98.6); ARG388(97.9); PHE398(98.0)
ESR1	8914	Nonan-1-ol	-4.891	10	97.49	95.81	LEU340(95.8); LEU343(96.7); ALA344(97.0); GLU347(97.4); LEU378(97.0); LEU381(98.2); MET382(98.2); LEU385(98.6); ARG388(97.9); PHE398(98.0)
ESR1	10393	2-(4-Hydroxyphenyl)ethanol	-5.897	8	97.46	95.81	LEU340(95.8); LEU343(96.7); ALA344(97.0); GLU347(97.4); LEU381(98.2); LEU385(98.6); ARG388(97.9); PHE398(98.0)
ESR1	18827	1-Octen-3-ol	-5.050	9	97.55	95.81	LEU340(95.8); LEU343(96.7); ALA344(97.0); GLU347(97.4); LEU381(98.2); MET382(98.2); LEU385(98.6); ARG388(97.9); PHE398(98.0)
ESR1	447765	(S)-Acetoin	-4.079	7	97.70	96.69	LEU343(96.7); ALA344(97.0); GLU347(97.4); LEU381(98.2); LEU385(98.6); ARG388(97.9); PHE398(98.0)
ESR1	689043	Caffeic acid	-6.646	8	97.00	94.25	MET337(94.2); LEU340(95.8); THR341(95.5); GLU347(97.4); LEU381(98.2); MET382(98.2); LEU385(98.6); ARG388(97.9)
ESR1	5280460	Scopoletin	-6.893	11	97.42	95.31	PRO318(97.1); PRO319(96.4); ILE320(95.3); VAL321(96.6); GLU347(97.4); MET351(98.1); ILE380(98.1); LEU381(98.2); GLY384(98.4); ARG388(97.9); LYS443(98.0)
ESR1	5280507	Sinapyl alcohol	-5.585	11	97.56	95.31	GLU317(97.9); PRO318(97.1); PRO319(96.4); ILE320(95.3); GLU347(97.4); ILE380(98.1); GLY384(98.4); TRP387(98.3); ARG388(97.9); PHE439(98.4); LYS443(98.0)
ESR1	5280802	Sinapaldehyde	-5.517	10	97.51	95.31	GLU317(97.9); PRO318(97.1); PRO319(96.4); ILE320(95.3); GLU347(97.4); GLY384(98.4); TRP387(98.3); ARG388(97.9); PHE439(98.4); LYS443(98.0)
ESR1	5283335	2-Nonenal	-4.814	7	97.39	95.81	LEU340(95.8); LEU343(96.7); ALA344(97.0); GLU347(97.4); LEU381(98.2); LEU385(98.6); PHE398(98.0)
HIF1A	8103	1-Hexanol	-4.538	8	93.67	89.12	LEU44(92.7); PRO45(90.2); LEU46(89.1); LEU62(95.6); TYR111(95.8); MET112(95.9); GLN124(93.8); THR128(96.3)
HIF1A	8914	Nonan-1-ol	-4.480	11	93.50	89.12	LEU44(92.7); PRO45(90.2); LEU46(89.1); LEU62(95.6); SER65(94.6); TYR66(94.1); ILE110(95.2); TYR111(95.8); GLN124(93.8); PHE125(91.2); THR128(96.3)

Target	CID	Compound	Vina score	Contacts n	Local mean pLDDT	Local min pLDDT	Contact residues with pLDDT
HIF1A	10393	2-(4-Hydroxyphenyl)ethanol	-5.297	10	93.46	89.12	LEU44(92.7); PRO45(90.2); LEU46(89.1); LEU62(95.6); TYR66(94.1); TYR111(95.8); MET112(95.9); GLN124(93.8); PHE125(91.2); THR128(96.3)
HIF1A	18827	1-Octen-3-ol	-5.032	10	93.72	89.12	PRO45(90.2); LEU46(89.1); LEU62(95.6); TYR66(94.1); ILE110(95.2); TYR111(95.8); MET112(95.9); GLN124(93.8); PHE125(91.2); THR128(96.3)
HIF1A	447765	(S)-Acetoin	-3.932	6	94.25	91.19	LEU62(95.6); SER65(94.6); TYR66(94.1); GLN124(93.8); PHE125(91.2); THR128(96.3)
HIF1A	689043	Caffeic acid	-5.783	9	93.96	90.12	PRO45(90.2); LEU62(95.6); SER65(94.6); TYR66(94.1); MET69(90.1); ILE110(95.2); TYR111(95.8); GLN124(93.8); THR128(96.3)
HIF1A	5280460	Scopoletin	-6.456	9	93.15	89.12	LEU44(92.7); PRO45(90.2); LEU46(89.1); LEU62(95.6); SER65(94.6); TYR66(94.1); MET69(90.1); TYR111(95.8); THR128(96.3)
HIF1A	5280507	Sinapyl alcohol	-5.514	10	91.63	82.06	LEU46(89.1); LEU62(95.6); TYR66(94.1); MET69(90.1); ARG70(88.3); LEU73(82.1); TYR111(95.8); GLN124(93.8); PHE125(91.2); THR128(96.3)
HIF1A	5280802	Sinapaldehyde	-5.495	11	91.50	82.06	PRO45(90.2); LEU46(89.1); LEU62(95.6); TYR66(94.1); MET69(90.1); ARG70(88.3); LEU73(82.1); TYR111(95.8); GLN124(93.8); PHE125(91.2); THR128(96.3)
HIF1A	5283335	2-Nonenal	-4.824	10	93.57	89.12	LEU46(89.1); LEU62(95.6); SER65(94.6); TYR66(94.1); MET69(90.1); ILE110(95.2); TYR111(95.8); GLN124(93.8); PHE125(91.2); THR128(96.3)
MMP9	8103	1-Hexanol	-4.819	9	94.06	88.19	ARG100(95.2); CYS101(95.6); LEU398(97.9); VAL399(98.6); LEU419(95.4); PRO422(90.4); MET423(90.9); TYR424(94.2); SER425(88.2)
MMP9	8914	Nonan-1-ol	-5.232	9	83.83	77.31	TYR130(86.4); SER131(87.1); PRO132(79.8); LEU134(83.6); ASP135(82.2); ARG136(83.9); ASN281(85.1); TYR313(77.3); PHE330(89.0)
MMP9	10393	2-(4-Hydroxyphenyl)ethanol	-6.542	11	94.25	88.19	ARG100(95.2); CYS101(95.6); LEU398(97.9); VAL399(98.6); HIS402(98.4); LEU419(95.4); TYR421(91.8); PRO422(90.4); MET423(90.9); TYR424(94.2); SER425(88.2)
MMP9	18827	1-Octen-3-ol	-5.620	12	94.45	88.19	ARG100(95.2); CYS101(95.6); LEU190(96.8); LEU398(97.9); VAL399(98.6); HIS402(98.4); LEU419(95.4); TYR421(91.8); PRO422(90.4); MET423(90.9); TYR424(94.2); SER425(88.2)
MMP9	447765	(S)-Acetoin	-4.926	11	95.18	90.44	ARG100(95.2); CYS101(95.6); LEU398(97.9); VAL399(98.6); HIS402(98.4); GLU403(98.4); LEU419(95.4); TYR421(91.8); PRO422(90.4); MET423(90.9); TYR424(94.2)
MMP9	689043	Caffeic acid	-7.399	13	94.67	88.19	ARG100(95.2); CYS101(95.6); LEU190(96.8); LEU398(97.9); VAL399(98.6); HIS402(98.4); GLU403(98.4); ALA418(89.2); LEU419(95.4); TYR421(91.8); MET423(90.9); TYR424(94.2); SER425(88.2)
MMP9	5280460	Scopoletin	-7.289	9	84.50	77.31	TYR130(86.4); SER131(87.1); PRO132(79.8); LEU134(83.6); ARG136(83.9); ASN281(85.1); TYR313(77.3); PHE330(89.0); CYS331(88.2)
MMP9	5280507	Sinapyl alcohol	-6.647	10	82.24	64.56	TYR130(86.4); PRO132(79.8); ARG136(83.9); ASN281(85.1); ARG309(81.8); ASP311(64.6); TYR313(77.3); ARG314(86.4); PHE330(89.0); CYS331(88.2)
MMP9	5280802	Sinapaldehyde	-6.705	11	82.69	64.56	TYR130(86.4); SER131(87.1); PRO132(79.8); ARG136(83.9); ASN281(85.1); ARG309(81.8); ASP311(64.6); TYR313(77.3); ARG314(86.4); PHE330(89.0); CYS331(88.2)
MMP9	5283335	2-Nonenal	-5.816	13	92.90	85.06	ARG100(95.2); CYS101(95.6); LEU190(96.8); LEU398(97.9); VAL399(98.6); GLU417(85.1); ALA418(89.2); LEU419(95.4); PRO422(90.4); MET423(90.9); TYR424(94.2); SER425(88.2); PHE430(90.2)

Table S2E-9. Same-species experimental SRC binding-site correspondence benchmark using PDB 2OIQ

PDB 2OIQ is a wild-type Gallus gallus SRC kinase-domain X-ray structure with imatinib (STI) at 2.07 Å resolution. Its PDB SITE record annotates the following 15 protein residues around imatinib: VAL281, ALA293, LYS295, GLU310, MET314, ILE336, THR338, TYR340, MET341, VAL383, HIS384, ARG385, ALA403, ASP404, and PHE405. The table below compares all nine standardized-rerun scopoletin-SRC modes with this same-species experimentally annotated site. This is a site-correspondence benchmark, not same-structure ligand RMSD redocking.

Mode	Vina score	2OIQ-site overlap n	Overlapping 2OIQ residues	Study-pose contact residues
1	-7.227	0	None	ARG156; GLU159; ARG160; LEU163; PRO361; GLN362; ASP365; ASN397; LEU398
2	-6.938	6	281; 293; 295; 338; 340; 341	LEU273; VAL281; ALA293; LYS295; THR338; GLU339; TYR340; MET341; GLY344; LEU393
3	-6.784	0	None	LEU89; TYR90; LYS104; GLN144; GLU146; GLU147; TYR149; PHE150; GLY151; THR247
4	-6.686	7	281; 293; 295; 338; 340; 341; 403	VAL281; ALA293; LYS295; VAL323; THR338; GLU339; TYR340; MET341; GLY344; LEU393; ALA403
5	-6.523	0	None	LEU89; TYR90; LYS104; GLN144; GLU147; TYR149; PHE150; THR247
6	-6.520	0	None	PRO250; GLN251; GLN253; ARG318; HIS319; GLU320; LEU322; GLN324; GLU339; LYS401
7	-6.517	6	281; 293; 295; 338; 340; 341	LEU273; VAL281; ALA293; LYS295; THR338; GLU339; TYR340; MET341; GLY344; LEU393
8	-6.493	0	None	LEU89; LYS104; GLN144; GLU147; TYR149; PHE150; THR247
9	-6.423	0	None	LEU89; TYR90; GLN144; ALA145; GLU146; GLU147; TYR149; PHE150; THR247

Interpretation: the lowest-scoring scopoletin-SRC mode showed 0/15 overlap with the 2OIQ imatinib-site residues, whereas modes 2, 4, and 7 recovered 6, 7, and 6 annotated site residues, respectively. Therefore, the receptor-encompassing search sampled the experimentally defined kinase pocket as well as noncanonical regions, but the Vina ranking did not uniquely identify the experimental pocket. Docking is consequently interpreted strictly as exploratory structural prioritization. Complete numeric data for the AlphaFold confidence audit and SRC benchmark are reported directly in Tables S2E-7 to S2E-9.

Table S2F. Chicken-specific GO/KEGG enrichment audit and complete-output inventory

The archived chicken-specific enrichment files were re-audited without pathway-name preselection. GO_analy.csv contains 228 archived GO rows with nominal $P < 0.05$ (165 BP, 7 CC, and 56 MF), of which 109 remain significant at Benjamini-Hochberg (BH)-adjusted $P < 0.05$ (78 BP, 2 CC, and 29 MF). KEGG_analy.csv contains 44 archived Gallus gallus pathways with nominal $P < 0.05$, of which 39 remain significant after BH adjustment. The BH procedure was independently verified by reconstructing the adjusted P values from the archived raw P values; the maximum absolute discrepancies were $< 1.3\times10^{-15}$ for GO and $< 2.8\times10^{-16}$ for KEGG. Complete row-level archived outputs are supplied in the accompanying spreadsheet, including term/pathway ID, description, GeneRatio, BgRatio, contributing genes, Count, raw P, BH-adjusted P, q value, multiple-testing correction method, RichFactor, FoldEnrichment, and z score.

Analysis	Effective annotated input	Effective background	Rows in archived output	BH-adjusted P < 0.05
GO-BP	121	11,696	165	78
GO-CC	95	11,619	7	2
GO-MF	129	12,431	56	29

Analysis	Effective annotated input	Effective background	Rows in archived output	BH-adjusted P < 0.05
KEGG (gga)	109	6,489	44	39

Table S2F-1. Enrichment-analysis parameters and archive-scope notes

Parameter	Audited value / statement
Input set	Final 133 representative high-confidence Gallus gallus orthologues.
Recorded software	R 4.2.1; clusterProfiler 4.6.2.
GO domains	Biological process (BP), cellular component (CC), and molecular function (MF).
KEGG organism	Gallus gallus, organism code gga.
Multiple-testing method	Benjamini-Hochberg (BH), verified independently from archived pvalue/p.adjust pairs.
Significance threshold	BH-adjusted P < 0.05.
Objective ranking for visualization	Adjusted P ascending, followed by GeneRatio descending and enriched-gene Count descending. Biological relevance was used only after statistical ranking for interpretation.
Complete-output fields	ID; Description; GeneRatio; BgRatio; Count; contributing genes; raw P; BH-adjusted P; q value; multiple-testing correction method; RichFactor; FoldEnrichment; z score.
Archive scope	The preserved GO_analy.csv and KEGG_analy.csv contain all rows retained at nominal P < 0.05 (228 GO and 44 KEGG). They are not claimed to contain every nonsignificant term/pathway tested by the original enrichment run.
Annotation/KEGG release	The exact historical annotation-database/KEGG release was not retained in the archived result files and is therefore not retrospectively assigned.
Minimum/maximum gene-set size	minGSSize = 5; maxGSSize was not set (no maximum gene-set-size parameter was specified).

Complete row-level output file. Supplementary_Table_S2_GO_KEGG_complete_archived.xlsx contains four sheets: GO_complete, KEGG_complete, Parameters, and README. GO_complete preserves all 228 rows in the archived GO_analy.csv, and KEGG_complete preserves all 44 rows in the archived KEGG_analy.csv. The Parameters sheet records the enrichment settings and archive-scope limitations; the README explains that these sheets represent the complete preserved archived outputs rather than a reconstruction of nonsignificant terms/pathways that were not saved by the original run.
